# Supplementary material for: Efficacy and safety of antifibrotic drugs for interstitial lung diseases other than IPF: A systematic review, meta-analysis and trial sequential analysis
Source: PLoS One. 2025 Feb 7;20(2):e0318877. doi: 10.1371/journal.pone.0318877 (PMC11805349; doi:10.1371/journal.pone.0318877)
Supplement: S1 File — (DOC) [file pone.0318877.s004.doc]

**SUPPORTING INFORMATION**

**Efficacy and safety of antifibrotic drugs for interstitial lung diseases other than IPF: a systematic review, meta-analysis and trial sequential analysis**

*Mei Yang, Yuying Tan, Ting Yang, Dan Xu, Mei Chen, Lei Chen*

**CONTENT**

[S1. Study protocol specifications 3](#__RefHeading___Toc265641860)

[S2. PRISMA checklist 4](#__RefHeading___Toc24238907)

[S3. Search strategy 8](#__RefHeading___Toc1508900666)

[S4. Assessment of random errors in trail sequential analysis (TSA) 10](#__RefHeading___Toc459106039)

[S5. Diagnosis and key eligibility criteria of patients across included studies 11](#__RefHeading___Toc286453802)

[S6. Analyses of primary outcomes 15](#__RefHeading___Toc1918197287)

[S7. Analyses of secondary outcomes 24](#__RefHeading___Toc1117293845)

[S8. Analyses of exploratory outcome 38](#__RefHeading___Toc760643547)

[S9. Subgroup analyses 39](#__RefHeading___Toc165943838)

[Table 1. Risk-of-bias assessment 54](#__RefHeading___Toc1584311460)

[Table 2. Overview of subgroup analyses in all participants 61](#__RefHeading___Toc872969067)

[Table 3. Overview of subgroup analyses in patients with a progressive fibrosing phenotype 68](#__RefHeading___Toc382832765)

[Table 4. Sensitivity analyses for all outcomes 70](#__RefHeading___Toc409274943)

[Table 5. GRADE evaluation of evidence in patients with a progressive fibrosing phenotype 73](#__RefHeading___Toc293845660)

[Figure 1. Funnel plot for all-cause mortality 74](#__RefHeading___Toc1599103167)

[References 75](#__RefHeading___Toc369085564)

**S1. Study protocol specifications**

|  | **Specifications** |
| --- | --- |
| **Study design** | This meta-analysis and trial sequential analysis (TSA) included studies regarding efficacy and safety of antifibrotic drug (pirfenidone or nintedanib) vs other intervention (placebo, conventional treatment or no intervention) in patients with ILDs other than IPF. |
| **Outcome measures** | 1. We planned to analyze a series of outcome measures including three primary outcomes, 14 secondary outcomes and one exploratory outcome in all participants with non-IPF ILDs and patients with a progressive fibrosing phenotype, respectively. 2. All the outcome measures were assessed from baseline to the latest time point within 6 to 12 months, unless otherwise stated. |
| **Subgroup analyses** | We planned to conduct subgroup analyses concerning:   1. Risk of bias (RoB) (low RoB vs some concerns or high risk), assessed by the Cochrane Collaboration tool of RoB2 or Newcastle-Ottawa Scale (NOS); 2. Antifibrotic drugs (pirfenidone vs nintedanib); 3. Duration of follow up (＜12 months vs ≥ 12 months); 4. ILD subtype; 5. HRCT pattern (UIP vs non-UIP); 6. Taking or not taking mycophenolate at baseline. |
| **RoB assessment** | 1. The quality of observational studies was assessed by the NOS, with a total score of 9. Considering the potential RoB of a non-RCT design, study with a total score of ＞5 was considered some concerns, whereas a score of ≤ 5 was considered high risk. 2. RoB of RCT was evaluated using the Cochrane Collaboration tool of RoB2. 3. The study of Li et al^1^ was a prospective study with matched retrospective controls and the study of wang et al^2^ applied a prospective cohort design, thus we considered both studies as observational studies. |
| **TSA and GRADE** | TSA and GRADE were conducted for trials with low RoB only. |

**S2. PRISMA checklist**

| **Section and Topic** | **Item #** | **Checklist item** | **Location where item is reported** |
| --- | --- | --- | --- |
| **TITLE** | | |  |
| Title | 1 | Identify the report as a systematic review. | Page 1 |
| **ABSTRACT** | | |  |
| Abstract | 2 | See the PRISMA 2020 for Abstracts checklist. | Page 2, 3 |
| **INTRODUCTION** | | |  |
| Rationale | 3 | Describe the rationale for the review in the context of existing knowledge. | Page 3, 4 |
| Objectives | 4 | Provide an explicit statement of the objective(s) or question(s) the review addresses. | Page 4 |
| **METHODS** | | |  |
| Eligibility criteria | 5 | Specify the inclusion and exclusion criteria for the review and how studies were grouped for the syntheses. | Page 5 |
| Information sources | 6 | Specify all databases, registers, websites, organisations, reference lists and other sources searched or consulted to identify studies. Specify the date when each source was last searched or consulted. | Page 5, 6 |
| Search strategy | 7 | Present the full search strategies for all databases, registers and websites, including any filters and limits used. | Page 6; S1 File |
| Selection process | 8 | Specify the methods used to decide whether a study met the inclusion criteria of the review, including how many reviewers screened each record and each report retrieved, whether they worked independently, and if applicable, details of automation tools used in the process. | Page 6 |
| Data collection process | 9 | Specify the methods used to collect data from reports, including how many reviewers collected data from each report, whether they worked independently, any processes for obtaining or confirming data from study investigators, and if applicable, details of automation tools used in the process. | Page 6, 7 |
| Data items | 10a | List and define all outcomes for which data were sought. Specify whether all results that were compatible with each outcome domain in each study were sought (e.g. for all measures, time points, analyses), and if not, the methods used to decide which results to collect. | Page 6 |
|  | 10b | List and define all other variables for which data were sought (e.g. participant and intervention characteristics, funding sources). Describe any assumptions made about any missing or unclear information. | Page 5, 6 |
| Study risk of bias assessment | 11 | Specify the methods used to assess risk of bias in the included studies, including details of the tool(s) used, how many reviewers assessed each study and whether they worked independently, and if applicable, details of automation tools used in the process. | Page 6, 7 |
| Effect measures | 12 | Specify for each outcome the effect measure(s) (e.g. risk ratio, mean difference) used in the synthesis or presentation of results. | Page 7 |
| Synthesis methods | 13a | Describe the processes used to decide which studies were eligible for each synthesis (e.g. tabulating the study intervention characteristics and comparing against the planned groups for each synthesis (item #5)). | Page 7 |
|  | 13b | Describe any methods required to prepare the data for presentation or synthesis, such as handling of missing summary statistics, or data conversions. | Page 7, 8 |
|  | 13c | Describe any methods used to tabulate or visually display results of individual studies and syntheses. | Page 7, 8 |
|  | 13d | Describe any methods used to synthesize results and provide a rationale for the choice(s). If meta-analysis was performed, describe the model(s), method(s) to identify the presence and extent of statistical heterogeneity, and software package(s) used. | Page 7, 8 |
|  | 13e | Describe any methods used to explore possible causes of heterogeneity among study results (e.g. subgroup analysis, meta-regression). | Page 8 |
|  | 13f | Describe any sensitivity analyses conducted to assess robustness of the synthesized results. | Page 8, 9 |
| Reporting bias assessment | 14 | Describe any methods used to assess risk of bias due to missing results in a synthesis (arising from reporting biases). | Page 6-9 |
| Certainty assessment | 15 | Describe any methods used to assess certainty (or confidence) in the body of evidence for an outcome. | Page 9 |
| **RESULTS** | | |  |
| Study selection | 16a | Describe the results of the search and selection process, from the number of records identified in the search to the number of studies included in the review, ideally using a flow diagram. | Page 9, Fig 1 |
|  | 16b | Cite studies that might appear to meet the inclusion criteria, but which were excluded, and explain why they were excluded. | Page 9, Fig 1 |
| Study characteristics | 17 | Cite each included study and present its characteristics. | Page 9, 10; Table 1, S1 File |
| Risk of bias in studies | 18 | Present assessments of risk of bias for each included study. | Page 10, S1 File |
| Results of individual studies | 19 | For all outcomes, present, for each study: (a) summary statistics for each group (where appropriate) and (b) an effect estimate and its precision (e.g. confidence/credible interval), ideally using structured tables or plots. | Figs 2-4;  S1 File |
| Results of syntheses | 20a | For each synthesis, briefly summarise the characteristics and risk of bias among contributing studies. | Page 10-16; S1 File |
|  | 20b | Present results of all statistical syntheses conducted. If meta-analysis was done, present for each the summary estimate and its precision (e.g. confidence/credible interval) and measures of statistical heterogeneity. If comparing groups, describe the direction of the effect. | Page 10-16,  S1 File |
|  | 20c | Present results of all investigations of possible causes of heterogeneity among study results. | Page 16,  S1 File |
|  | 20d | Present results of all sensitivity analyses conducted to assess the robustness of the synthesized results. | Page 16, S1 File |
| Reporting biases | 21 | Present assessments of risk of bias due to missing results (arising from reporting biases) for each synthesis assessed. | S1 File |
| Certainty of evidence | 22 | Present assessments of certainty (or confidence) in the body of evidence for each outcome assessed. | Page 10-16, Table 2, S1 File |
| **DISCUSSION** | | |  |
| Discussion | 23a | Provide a general interpretation of the results in the context of other evidence. | Page 22 |
|  | 23b | Discuss any limitations of the evidence included in the review. | Page 21, 22 |
|  | 23c | Discuss any limitations of the review processes used. | Page 21, 22 |
|  | 23d | Discuss implications of the results for practice, policy, and future research. | Page 16-22 |
| **OTHER INFORMATION** | | |  |
| Registration and protocol | 24a | Provide registration information for the review, including register name and registration number, or state that the review was not registered. | Page 4 |
|  | 24b | Indicate where the review protocol can be accessed, or state that a protocol was not prepared. | Page 4, S1 File |
|  | 24c | Describe and explain any amendments to information provided at registration or in the protocol. | Not applicable |
| Support | 25 | Describe sources of financial or non-financial support for the review, and the role of the funders or sponsors in the review. | Cover letter |
| Competing interests | 26 | Declare any competing interests of review authors. | The submission system |
| Availability of data, code and other materials | 27 | Report which of the following are publicly available and where they can be found: template data collection forms; data extracted from included studies; data used for all analyses; analytic code; any other materials used in the review. | The submission system |

**S3. Search strategy**

**PubMed**

Searched November 9^th^, 2023 Records identified: 4780

Updated search May 20^th^, 2024 Records identified: 5114

1. (("Lung Diseases, Interstitial/drug therapy"[Majr]) NOT "Idiopathic Pulmonary Fibrosis"[Mesh]) OR ((("interstitial lung diseases"[Title/Abstract] OR "pulmonary fibrosis"[Title/Abstract] OR "non-IPF"[Title/Abstract] OR (("lung"[MeSH Terms] OR "lung"[All Fields]) AND "fbrosis"[Title/Abstract]) OR "CTD-ILD"[Title/Abstract] OR "connective tissue disease"[Title/Abstract] OR "fibrosing interstitial lung disease"[Title/Abstract] OR "hypersensitivity pneumonia"[Title/Abstract]) AND ("drugs"[Title/Abstract] OR "pirfenidone"[Title/Abstract] OR "Nintedanib"[Title/Abstract])) NOT ("idiopathic pulmonary fibrosis"[MeSH Terms] OR ("idiopathic"[All Fields] AND "pulmonary"[All Fields] AND "fibrosis"[All Fields]) OR "idiopathic pulmonary fibrosis"[All Fields]))
2. Filters applied: English

**Ovid EMBASE**

Searched November 9^th^, 2023 Records identified: 972

Updated search May 20^th^, 2024 Records identified: 1216

1. (((interstitial lung diseases) OR (pulmonary fibrosis) OR (non-IPF) OR (lung fibrosis)) AND (drugs OR pirfenidone OR Nintedanib OR Antifibrotic)) NOT (idiopathic pulmonary fibrosis) {No Related Terms}

2. Limit to English language

**Cochrane Library**

Searched November 9^th^, 2023 Records identified: 285

Updated search May 20^th^, 2024 Records identified: 514

#1 (interstitial lung diseases):ti,ab,kw in Trials

#2 (pulmonary fibrosis):ti,ab,kw in Trials

#3 (non-IPF):ti,ab,kw in Trials

#4 (lung fibrosis):ti,ab,kw in Trials

#5 #1 or #2 or #3 or #4

#6 (drugs):ti,ab,kw in Trials

#7 (pirfenidone):ti,ab,kw in Trials

#8 (Nintedanib):ti,ab,kw in Trials

#9 (Antifibrotic):ti,ab,kw in Trials

#10 #6 or #7 or #8 or #9

#11 English:la in Trials

#12 #5 AND #10 AND #11

**ClinicalTrials.gov**

Searched November 9^th^, 2023 Records identified: 152

Updated search May 20^th^, 2024 Records identified: 156

#1 Condition/disease: Interstitial Lung Disease

#2 Other terms: Pulmonary Fibrosis

#3 Intervention/treatment: anti fibrotic

#4 #1 AND #2 AND #3

#5 Intervention/treatment: Pirfenidone

#6 #1 AND #2 AND #5

#7 Intervention/treatment: Nintedanib

#8 #1 AND #2 AND #7

#9 #4 or #6 or #8

**S4. Assessment of random errors in trail sequential analysis (TSA)**

In TSA, we quantified the trial sequential monitoring boundaries using a β of 10% (power of 90%) and a family-wise error rate of 5%. Since the Bonferoni corrections may be too conservative, we applied a pragmatic approach suggested by Jakobsen et al.^3^ We divided the pre-specified family-wise error rate of 5% with the value halfway between 1 (no adjustment) and the number of outcome comparisons (Bonferroni adjustment) for primary, secondary and exploratory outcomes respectively.

For the three primary outcomes, the type I error (α) = 0.05 / 2

For the 14 secondary outcomes, the type I error (α) = 0.05 / 7

For the one exploratory outcome, the type I error (α) = 0.05 / 1

**S5. Diagnosis and key eligibility criteria of patients across included studies**

| **Study** | **Diagnosis and key inclusion criteria** |
| --- | --- |
| Distler et al, 2019^4^  (SSc-ILD) | 1. ≥ 18 years; 2. Having SSc according to the ACR 2013 classification criteria^5^, with an onset of the first non-Raynaud’ s symptom within 7 years before screening; 3. Fibrosis affecting at least 10% of the lungs on HRCT; 4. FVC ≥ 40% predicted and DLCO between 30%-89% predicted. |
| Flaherty et al, 2019^6^  (Progressive fibrosing ILDs) | 1. ≥ 18 years; 2. Fibrosing lung disease affecting more than 10% of lung volume on HRCT; 3. FVC ≥ 45% predicted and DLCO between 30%-80% predicted; 4. Meeting at least one of the following criteria for progression of ILD within the 24 months before screening, despite standard treatment with an agent other than nintedanib or pirfenidone: 5. a relative decline in FVC ≥ 10% predicted; 6. a relative decline in FVC between 5%-10% predicted, with worsening of respiratory symptoms or an increased extent of fibrosis on HRCT; 7. worsening of respiratory symptoms and an increased extent of fibrosis. |
| Acharya et al, 2020^7^  (SSc-ILD) | 1. Having SSc according to the ACR 2013 classification criteria^5^, with a disease duration of less than 7 years since the onset of the first non-Raynaud’ s symptom; 2. No new immunosuppressive treatment administered in the previous 6 months; 3. Confirmed ILD on HRCT; 4. FVC between 50%-80% predicted and DLCO＞30% predicted. |
| Maher et al, 2020^8^  (Unclassifiable progressive fibrosing ILD) | 1. Between 18-85 years; 2. Having fibrosing ILD that could not be classified with moderate or high confidence to any category of ILD after multidisciplinary team discussion at each centre; 3. More than 10% fibrosis on HRCT, which conducted within the previous 12 months; 4. FVC ≥ 45% predicted and DLCO ≥ 30% predicted; 5. FEV_1_ / FVC ratio ≥ 0.7 and 6MWD ≥ 150m; 6. Progressive fibrosis was defined as either (1) a more than 5% absolute decline in FVC% predicted or (2) significant symptomatic worsening not due to cardiac, pulmonary (except worsening of underlying unclassifiable ILD), vascular, or other causes (as determined by the investigator) within the previous 6 months. |
| Mateos-Toledo et al, 2020^9^  (cHP) | 1. Having HP confirmed by a multidisciplinary team, based on a history of exposure to organic antigens and/or laboratory proof of exposure (serum specific IgG), BAL lymphocytosis (performed in most of the patients), characteristic HRCT findings including the presence of micronodules, ground glass attenuation and mosaic attenuation, and when available, histopathological features compatible with HP; 2. cHP was defined as: 3. more than 12 months of symptoms before diagnosis; 4. HRCT displaying in addition to nodules and ground glass attenuation, fibrotic lesions (defined as the presence of reticulation, traction bronchiectasis and/or honeycombing); 5. when available, presence of fibrosis and architectural distortion in the histopathological evaluation of the lung biopsy affecting more than 10%. 6. Not receiving corticosteroids or immunosuppressive drugs at the time of diagnosis. |
| Behr et al, 2021^10^  (Progressive fibrosing ILDs) | 1. Between 18-80 years; 2. FVC between 40%-90% predicted; 3. DLCO between 25%-75% predicted, further extended to 10%-90% as part of a protocol amendment to increase the enrolment rate on July 7, 2016; 4. Progressive fibrosis (despite conventional therapy) was defined as an annual FVC decline ≥ 5% predicted, based on at least 3 measurements within 6-24 months before enrolment. |
| Shebl et al, 2021^11^  (Progressive fibrosing cHP) | 1. ≥ 18 years old with a diagnosis of progressive fibrosing cHP; 2. > 10% extent of fibrosis (e.g., reticulation) on HRCT; 3. Progressive fibrosis was defined as an absolute decline in FVC > 5% predicted within the previous 6 months despite conventional treatment. |
| Fernández Pérez et al, 2023^12^  (Fibrotic HP) | 1. Between 18-80 years; 2. Having a diagnosis of fibrotic HP or with a provisional high-confidence diagnosis based on multidisciplinary consensus discussion and integration of the clinical, imaging, bronchoscopic and, when available, surgical lung biopsy data; 3. FVC ≥ 40% predicted and DLCO ≥ 30% predicted; 4. Having fibrotic abnormalities affecting ≥5% of the lungs by visual assessment (reticular abnormality and/or, traction bronchiectasis and/or, architectural distortion, and/or honeycombing and with no evidence or suspicion of an alternate diagnosis), performed within 4 months of enrolment as confirmed by a blinded thoracic radiologist; 5. Progressive fibrosis was defined as worsening respiratory symptoms and (1) either an increase in the extent of fibrosis on HRCT or (2) relative decline in prebronchodilator FVC ≥ 5% within the 24 months before screening. |
| Rimner et al, 2023^13^  (RP) | 1. ≥ 18 years; 2. Newly diagnosed with clinical G2+ RP according to CTCAE (version 4.0) and history of thoracic malignancy or lung metastasis treated with definitive intent; 3. Receiving prior thoracic radiation therapy between 4 weeks and 9 months before enrollment and Karnofsky Performance Status score ≥70. |
| Solomon et al, 2023^14^  (RA-ILD) | 1. 18-85 years; 2. Diagnosed with RA according to the 2010 American College of Rheumatology-European League Against Rheumatism criteria^15^ and ILD based on HRCT presentation and when available, surgical lung biopsy; 3. Fibrotic abnormality affecting more than 10% of the lung parenchyma, with or without traction bronchiectasis or honeycombing on HRCT, and with no evidence or suspicion of an alternate diagnosis, as confirmed by a centrally adjudicated expert read; 4. FVC ≥ 40% predicted and DLCO ≥ 30% predicted; 5. Having a relative change in prebronchodilator FVC less than 10% between the screening visit (visit one) and baseline study visit (visit two). |
| Li et al, 2016^1^  (CADM-RPILD) | 1. All patients fulfilled the provisional diagnosis of CADM according to the modified Sontheimer’ s criteria;^5,16-18^ 2. RPILD was defined as disease exacerbation within 6 months of the onset of ILD, presenting as increased levels of dyspnea and worsening of fibrosis on pulmonary HRCT with >10% increase of the HRCT score and/or a reduction in %FVC by >10% of the absolute value. |
| Wang et al, 2022^2^  CTD-ILD | 1. ≥ 18 years; 2. Meeting the international classification standard of CTD and undifferentiated CTD (UCTD);^19^ 3. The ILD diagnosis conformed to the criteria of HRCT and progressive fibrosis formulated by the American Thoracic Association and the European Respiratory Association in 2002.^20^ |

The key inclusion criteria of five post-hoc analyses were consistent with the original RCTs, thus data were not shown.

ACR = the classification criteria of the American College of Rheumatology and European League Against Rheumatism; CADM-RPILD = rapidly progressive interstitial lung disease associated with clinically amyopathic dermatomyositis; cHP = chronic hypersensitivity pneumonitis; CTCAE = the National Cancer Institute Common Terminology Criteria for Adverse Events; CTD = connective tissue disease; FHP = fibrotic hypersensitivity pneumonitis; ILD = interstitial lung disease; RA = rheumatoid arthritis; RP = radiation pneumonitis; SSc = systemic sclerosis.

**S6. Analyses of primary outcomes**

**1. Absolute change in FVC (ml)**

**1.1 For all participants**

**1.1.1 Meta-analysis of absolute change in FVC (antifibrotic drugs vs. control)**


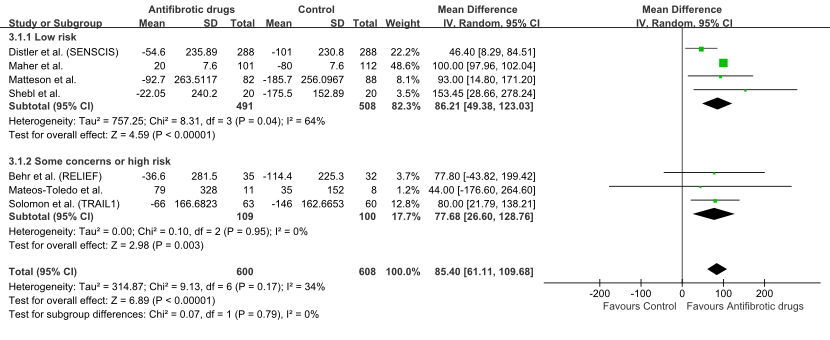


**1.1.2 TSA for absolute change in FVC (trials with low RoB)**


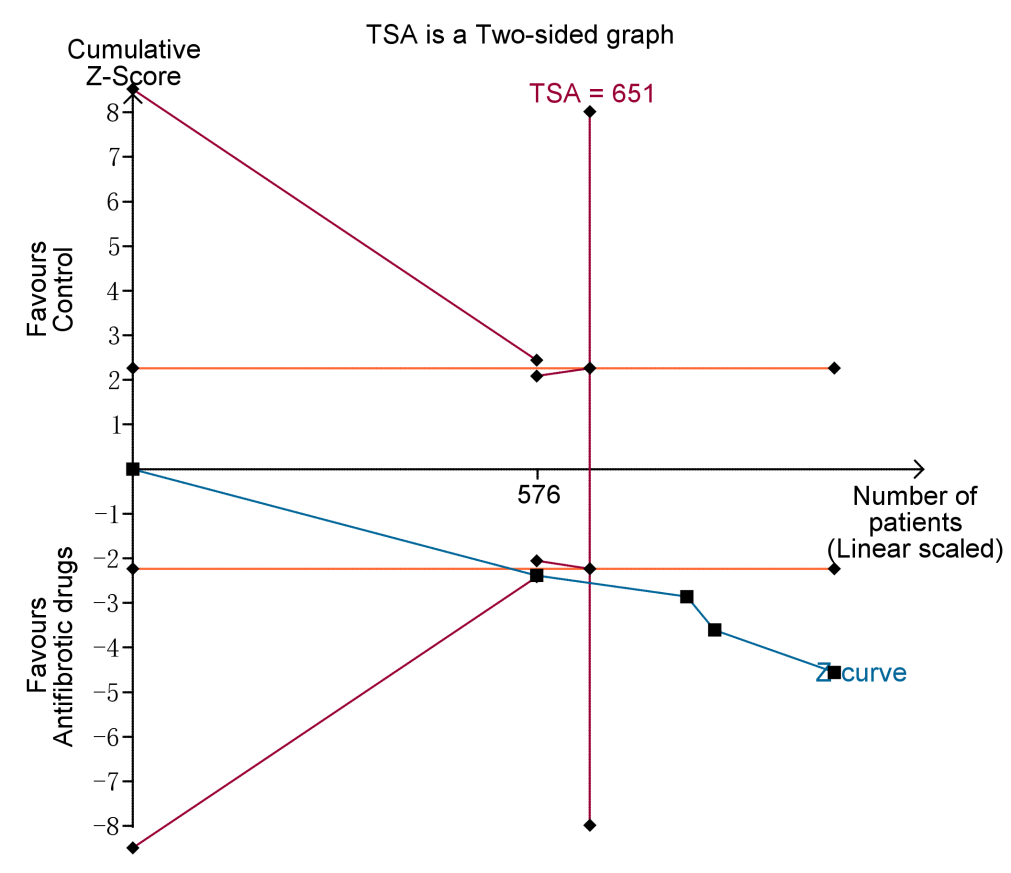


TSA for four trials with low RoB. The required information size (RIS) was calculated based on mean difference (Empirical), variance (Empirical), α of 2.5% and β of 10%. The cumulative z curve crossed both the conventional and trial sequential monitoring boundaries for benefit, with more than the RIS of 615 patients accrued. Thus the TSA is conclusive, with TSA-adjusted CI of 40.86 to 131.56 (random effects model) and a diversity D^2^ of 100%.

**1.2 For participants with a progressive fibrosing phenotype**

**1.2.1 Meta-analysis of absolute change in FVC (antifibrotic drugs vs. control)**

**
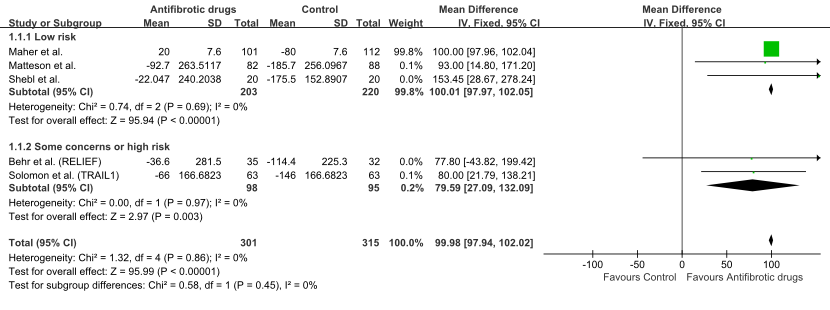
**

**1.2.2 TSA for absolute change in FVC (trials with low RoB)**


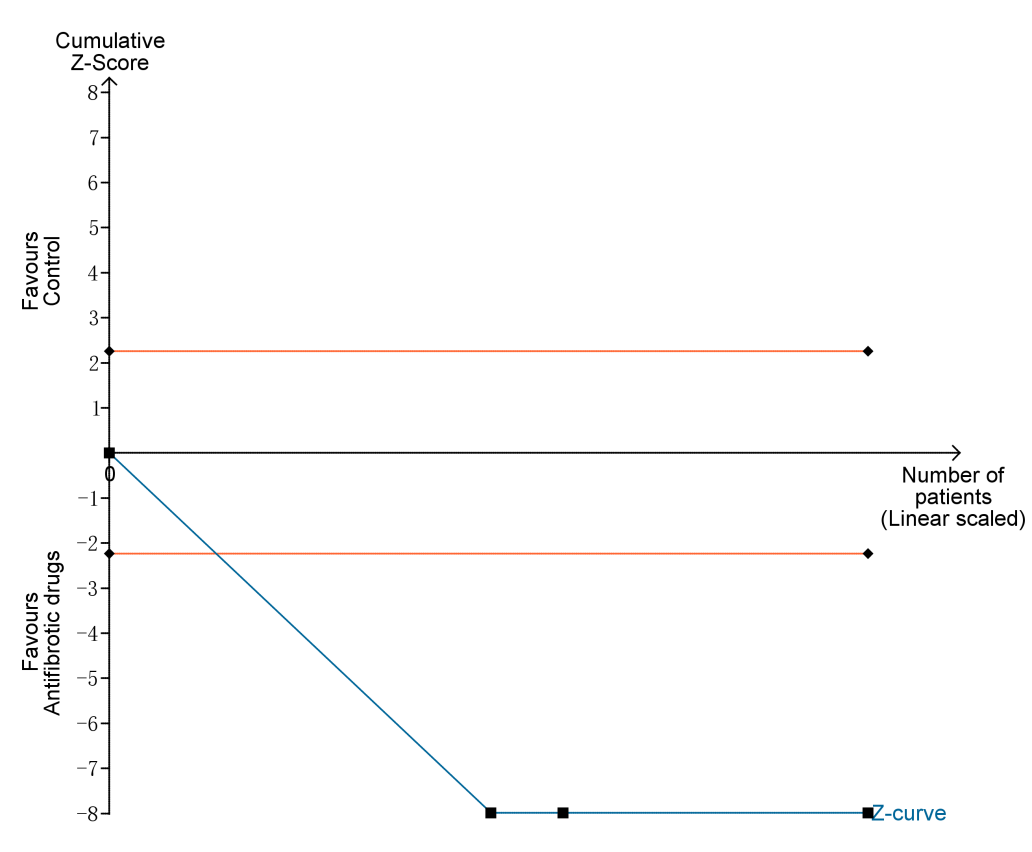


TSA for three trials with low RoB. The RIS was calculated based on mean difference (Empirical), variance (Empirical), α of 2.5% and β of 10%. TSA suggested that the RIS was reached within the first trial. Thus the TSA is conclusive, with a diversity D^2^ of 0%.

**2. All-cause mortality**

**2.1 For all participants**

**2.1.1 Meta-analysis of all-cause mortality (antifibrotic drugs vs. control)**


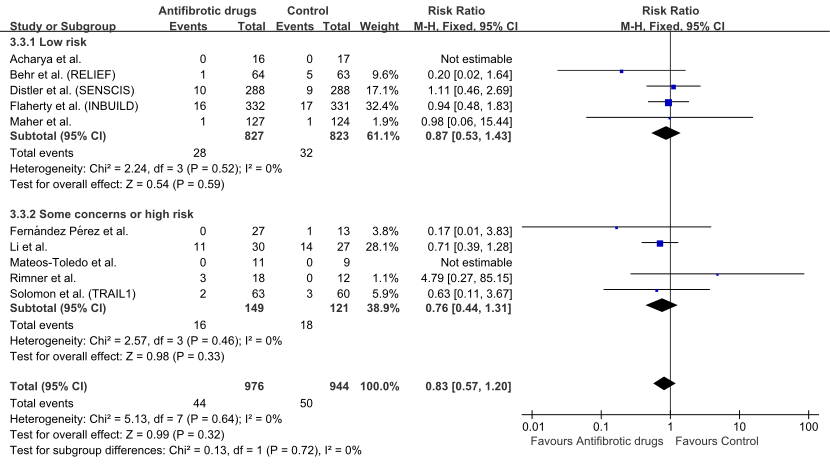


**2.1.2 TSA for all-cause mortality (trials with low RoB)**

(1) Applying a relative risk reduction (RRR) based on trials with low RoB


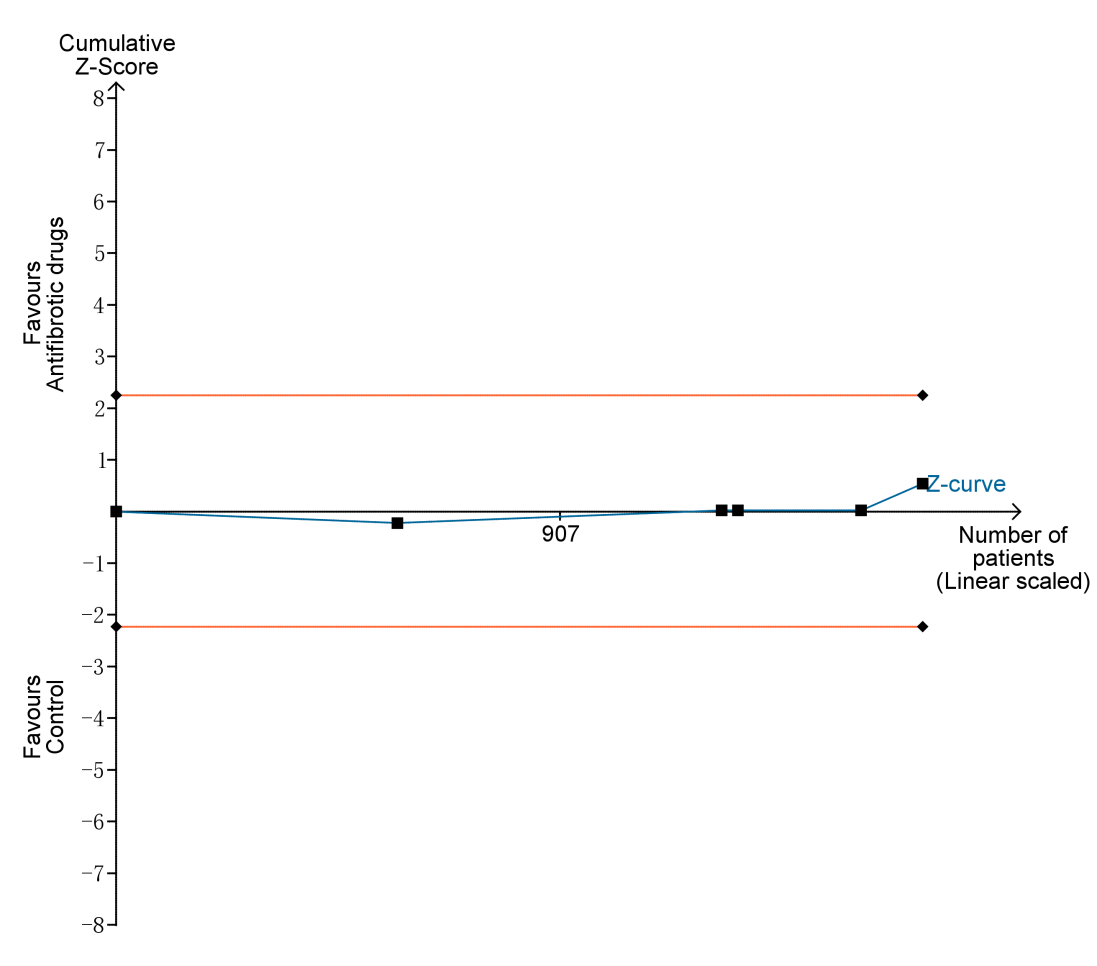


TSA for five trials with low RoB. The RIS was calculated according to α of 2.5%, β of 10%, control event rate of 5.3% (based on all included studies), RRR based on trials with low RoB, model variance-based heterogeneity adjustment. The cumulative z curve neither crossed the conventional not the TSA boundary for benefit, harm or futility, with only 3.2% of the RIS of 52,183 patients accrued. Thus the TSA is inconclusive, with a diversity D^2^ of 0%.

(2) Applying a RRR of 20%


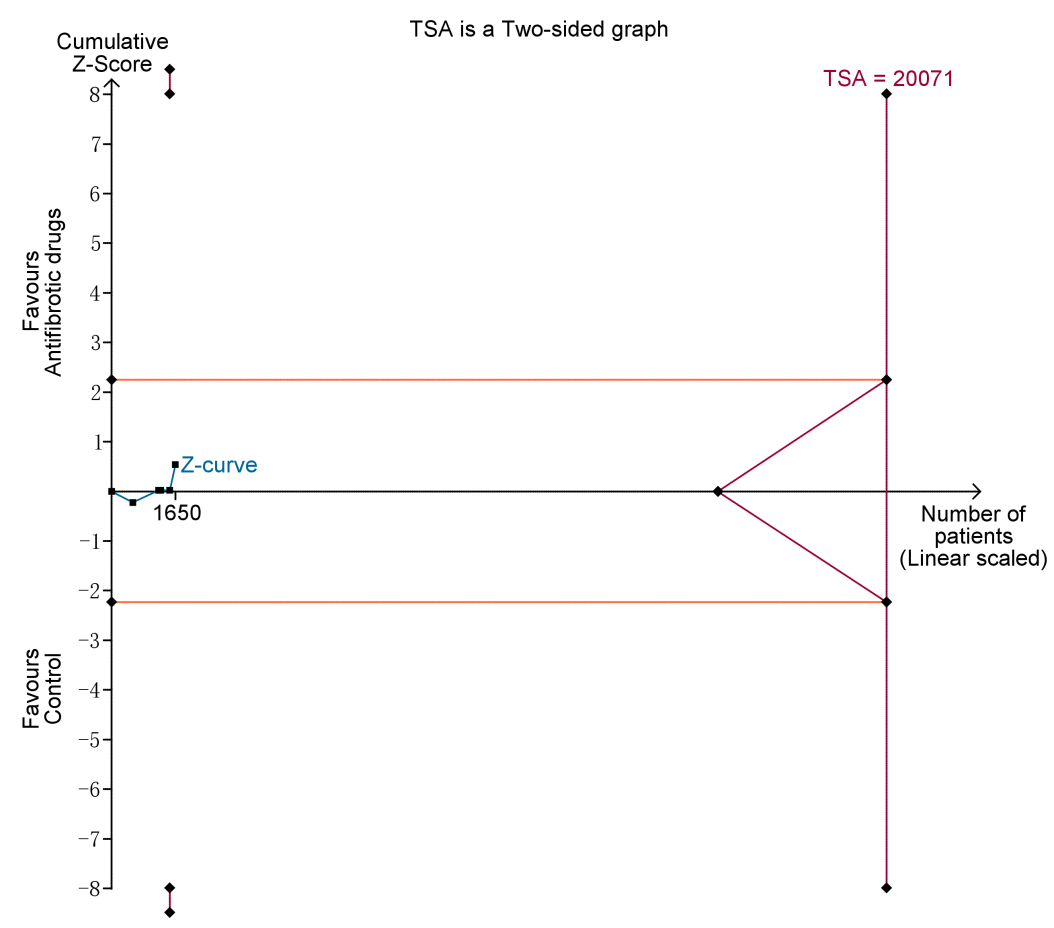


TSA for five trials with low RoB. The RIS was calculated according to α of 2.5%, β of 10%, control event rate of 5.3% (based on all included studies), RRR of 20%, model variance-based heterogeneity adjustment. The cumulative z curve crossed neither the conventional nor the TSA boundary for benefit, harm or futility, with 8.2% of the RIS of 20,071 patients accrued. Thus the TSA is inconclusive, with TSA-adjusted CI of 0.12 to 6.53 (fixed effect model) and a diversity D^2^ of 0%.

**2.2 For participants with a progressive fibrosing phenotype**

**2.2.1 Meta-analysis of all-cause mortality (antifibrotic drugs vs. control)**

**
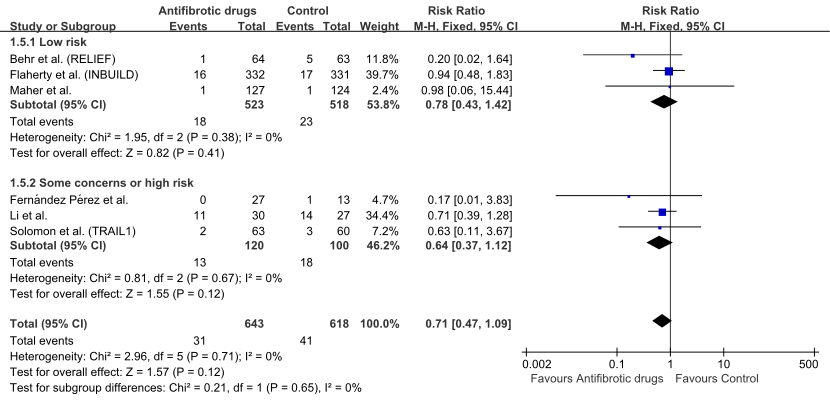
**

**2.2.2 TSA for all-cause mortality (trials with low RoB)**


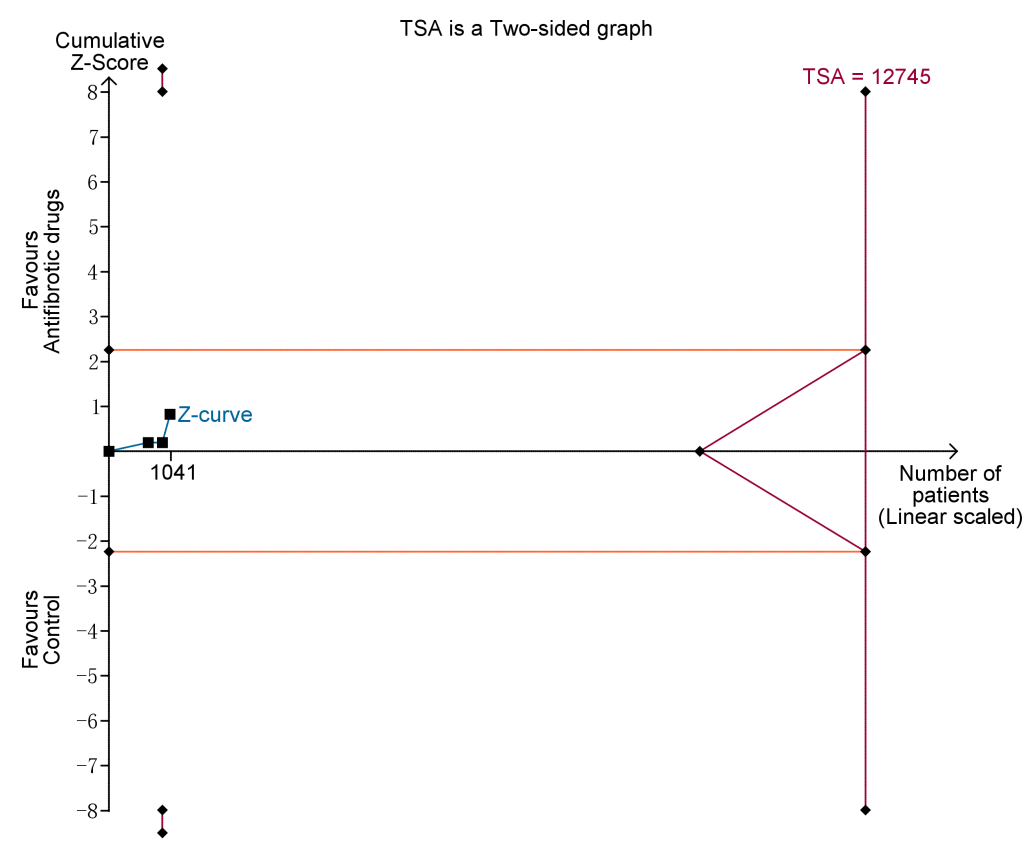


TSA for three trials with low RoB. The RIS was calculated according to α of 2.5%, β of 10%, control event rate of 6.6% (based on all included studies), RRR based on trials with low RoB, model variance-based heterogeneity adjustment. The cumulative z curve crossed neither the conventional nor the TSA boundary for benefit, harm or futility, with 8.2% of the RIS of 12,745 patients accrued. Thus the TSA is inconclusive, with TSA-adjusted CI of 0.07 to 9.11 (fixed effect model) and a diversity D^2^ of 0%.

**3. Serious adverse events (SAEs)**

**3.1 For all participants**

**3.1.1 Meta-analysis of SAEs (antifibrotic drugs vs. control)**


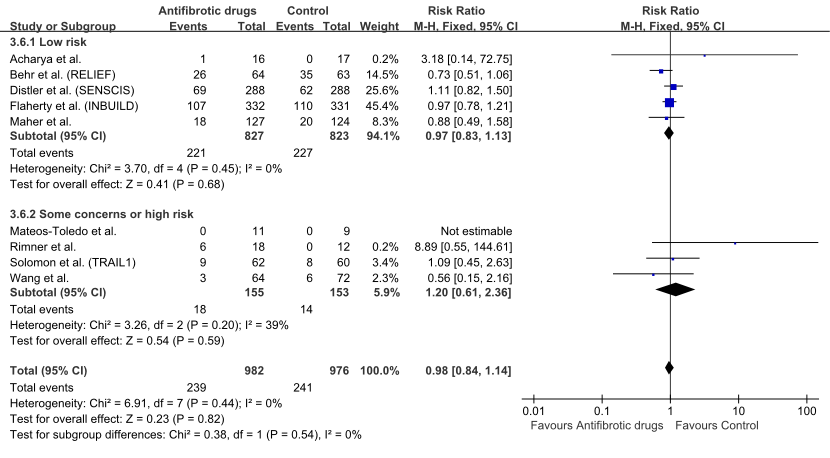


**3.1.2 TSA for SAEs (trials with low RoB)**

(1) Applying a RRR based on trials with low RoB


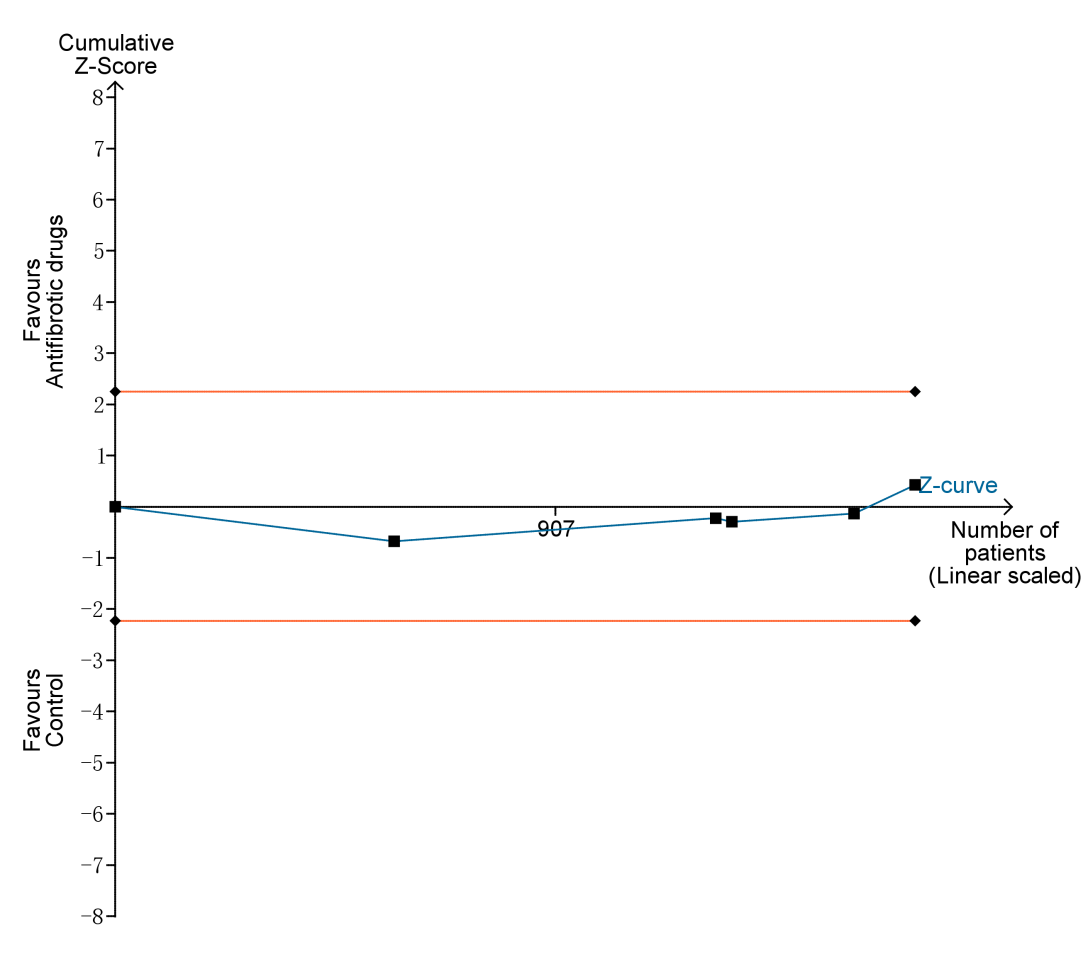


TSA for five trials with low RoB. The RIS was calculated according to α of 2.5%, β of 10%, control event rate of 24.92% (based on all included studies), RRR based on trials with low RoB, model variance-based heterogeneity adjustment. The cumulative z curve neither crossed the conventional nor the TSA boundary for benefit, harm or futility, with only 1.1% of the RIS of 147,243 patients accrued. Thus the TSA is inconclusive, with TSA-adjusted CI of 0.83 to 1.13 (fixed effect model) and a diversity D^2^ of 0%.

(2) Applying a RRR of 20%


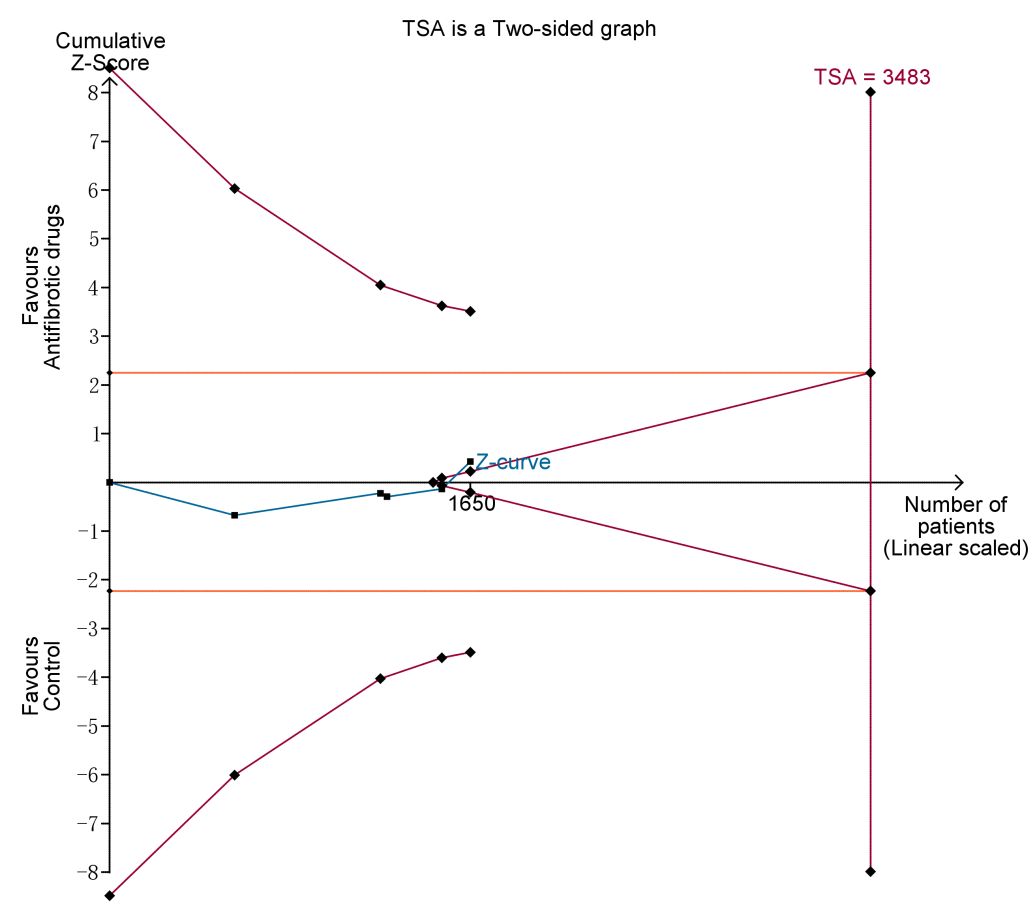


TSA for five trials with low RoB. The RIS was calculated according to α of 2.5%, β of 10%, control event rate of 24.92% (based on all included studies), RRR of 20%, model variance-based heterogeneity adjustment. The cumulative z curve neither crossed the conventional nor the TSA boundary for benefit, harm or futility, with 47.4% of the RIS of 3,483 patients accrued. Thus the TSA is inconclusive, with TSA-adjusted CI of 0.74 to 1.28 (fixed effect model) and a diversity D^2^ of 0%.

**3.1.3 Meta-analysis of fatal AEs, for all participants (antifibrotic drugs vs. control)**

**
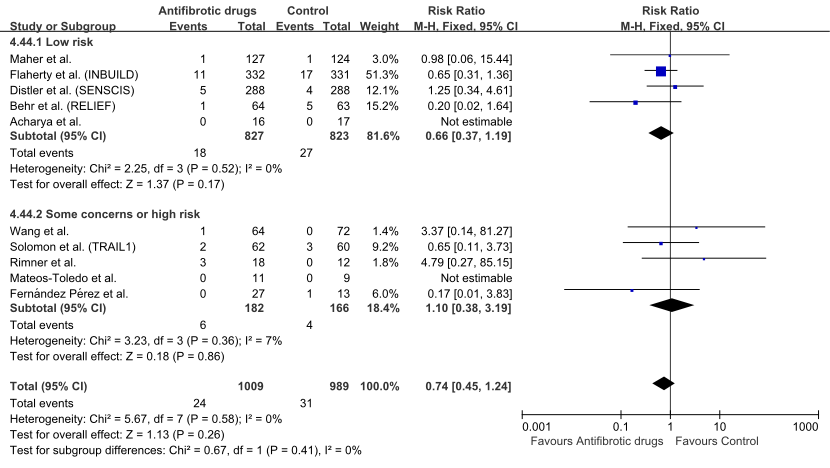
**

**3.1.4 TSA for fatal AEs (trials with low RoB)**

**
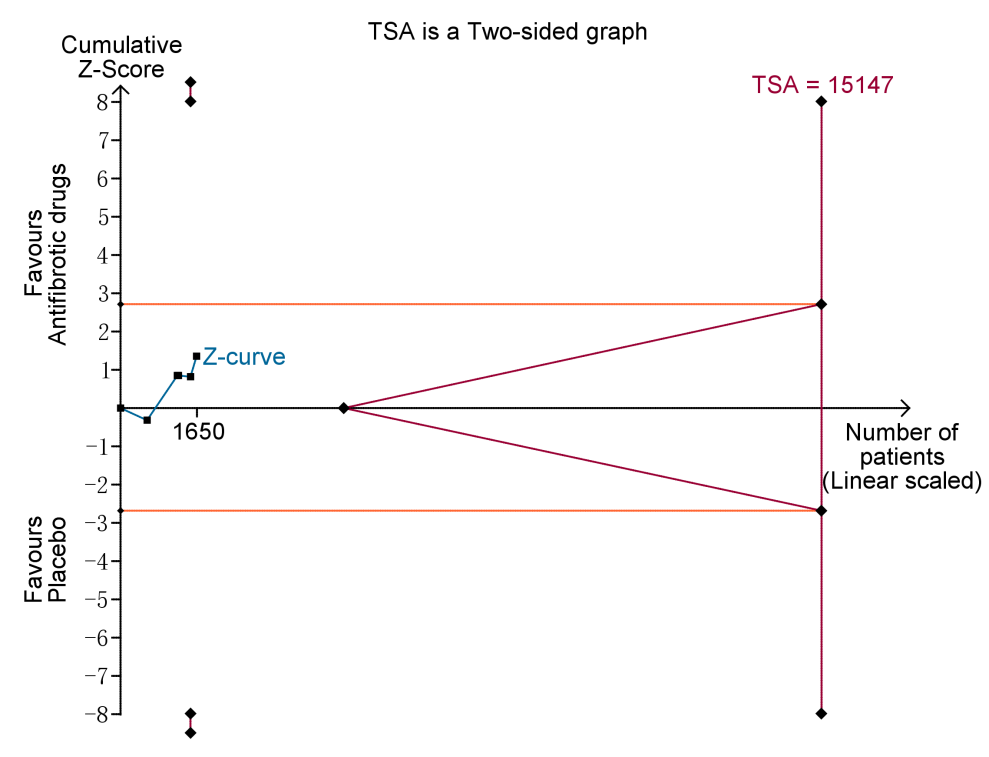
**

TSA for five trials with low RoB. The RIS was calculated according to α of 0.71%, β of 10%, control event rate of 3.1% (based on all included studies), RRR based on trials with low RoB, model variance-based heterogeneity adjustment. The cumulative z curve neither crossed the conventional nor the TSA boundary for benefit, harm, or futility, with 10.9% of the RIS of 15,147 patients accrued. Thus the TSA is inconclusive, with TSA-adjusted CI of 0.06 to 7.14 (fixed effect model) and a diversity D^2^ of 0%.

**3.2 For participants with a progressive fibrosing phenotype**

**3.2.1 Meta-analysis of SAEs (antifibrotic drugs vs. control)**

**
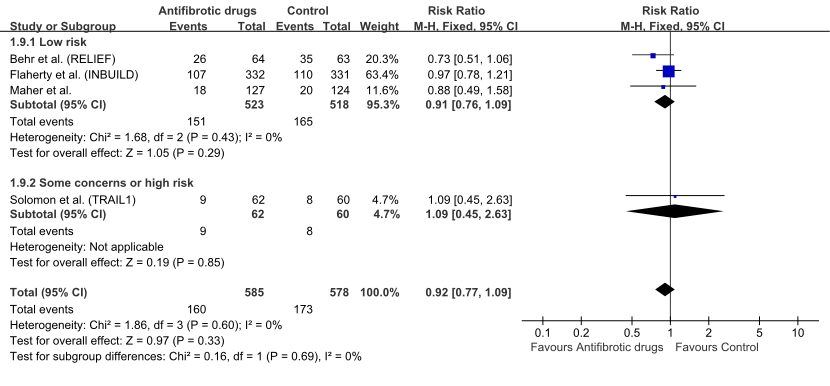
**

**3.2.2 TSA for SAEs (trials with low RoB)**


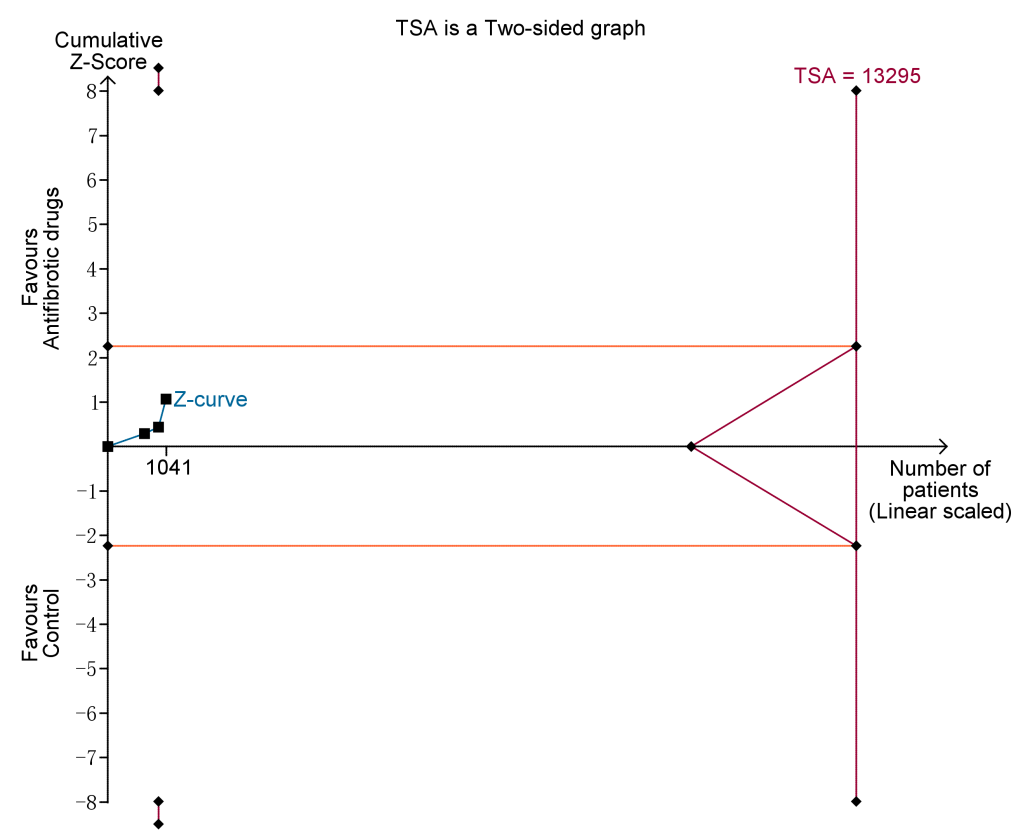


TSA for three trials with low RoB. The RIS was calculated according to α of 2.5%, β of 10%, control event rate of 29.9% (based on all included studies), RRR based on trials with low RoB, model variance-based heterogeneity adjustment. The cumulative z curve neither crossed the conventional nor the TSA boundary for benefit, harm or futility, with 7.8% of the RIS of 13,295 patients accrued. Thus the TSA is inconclusive, with TSA-adjusted CI of 0.44 to 1.89 (fixed effect model) and a diversity D^2^ of 0%.

**S7. Analyses of secondary outcomes**

Based on limited data, we could only analyze the outcome of absolute change in FVC% predicted in patients with a progressive fibrosing phenotype.

**1. Absolute change in FVC% predicted**

**1.1 For all participants**

**1.1.1 Meta-analysis of absolute change in FVC% predicted (antifibrotic drugs vs. control)**


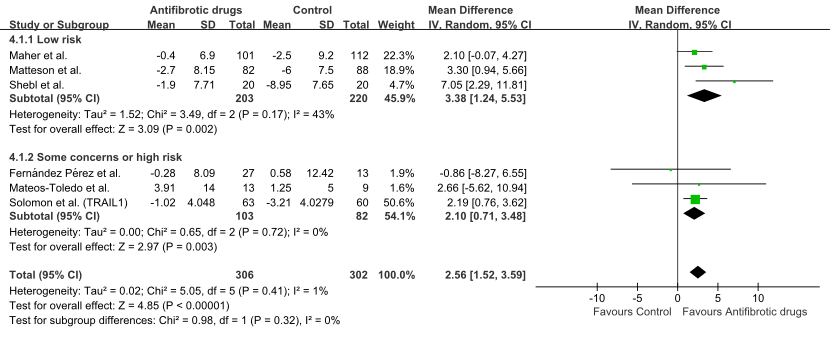


**1.1.2 TSA for absolute change in FVC% predicted (trials with low RoB)**


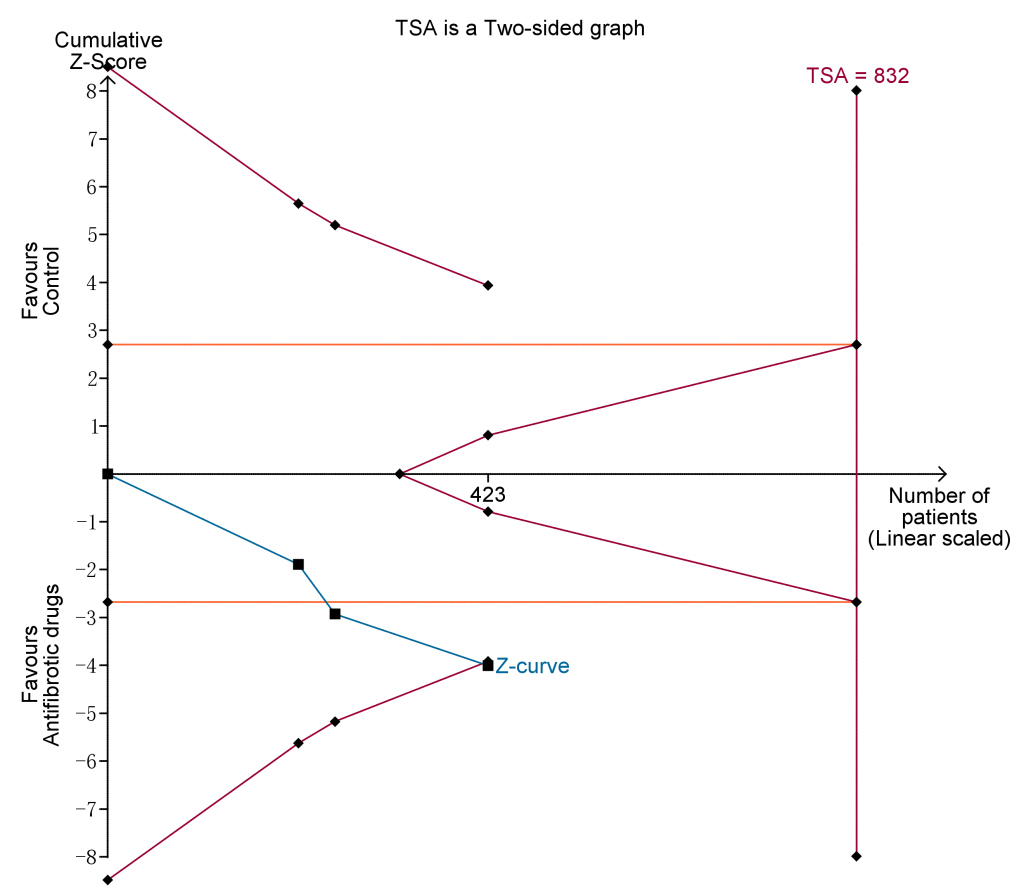


TSA for three trials with low RoB. The RIS was calculated based on mean difference (Empirical), variance (Empirical), α of 0.71% and β of 10%. The cumulative z curve crossed the TSA boundary for benefit, with 50.8% of the RIS of 832 patients accrued. Thus the TSA is conclusive, with TSA-adjusted CI of 0.34 to 5.86 (random-effects model) and a diversity D^2^ of 50%.

**1.2 For participants with a progressive fibrosing phenotype**

**1.2.1 Meta-analysis of absolute change in FVC% predicted (antifibrotic drugs vs. control)**


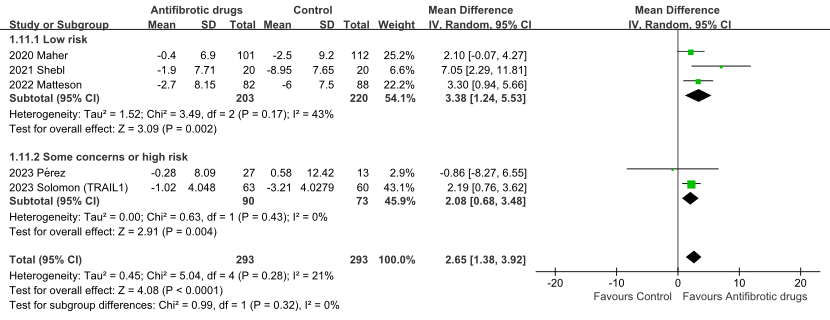
**1.2.2 TSA for absolute change in FVC% predicted (trials with low RoB)**


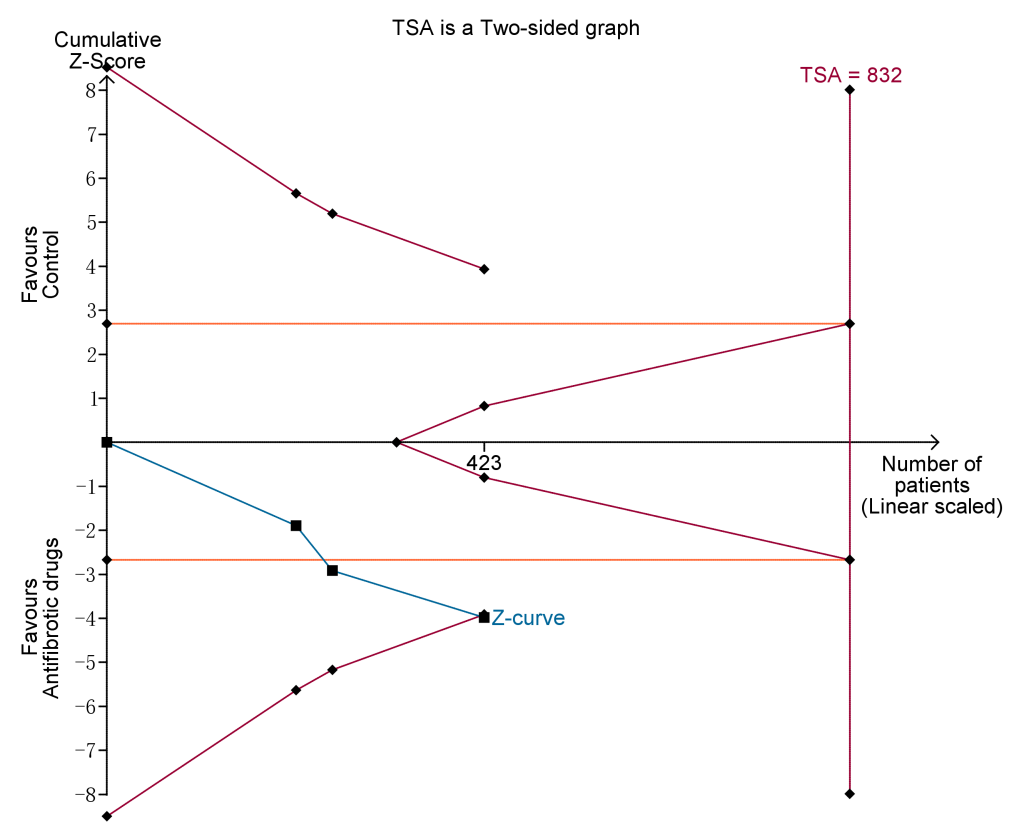


TSA for three trials with low RoB. The RIS was calculated based on mean difference (Empirical), variance (Empirical), α of 0.71% and β of 10%. The cumulative z curve crossed the TSA boundary for benefit, with 50.8% of the RIS of 832 patients accrued. Thus the TSA is conclusive, with TSA-adjusted CI of 0.34 to 5.86 (random-effects model) and a diversity D^2^ of 50%.

**2. Absolute decline in FVC ≥ 10% predicted, for all participants**

**2.1 Meta-analysis of absolute decline in FVC ≥ 10% predicted (antifibrotic drugs vs. control)**


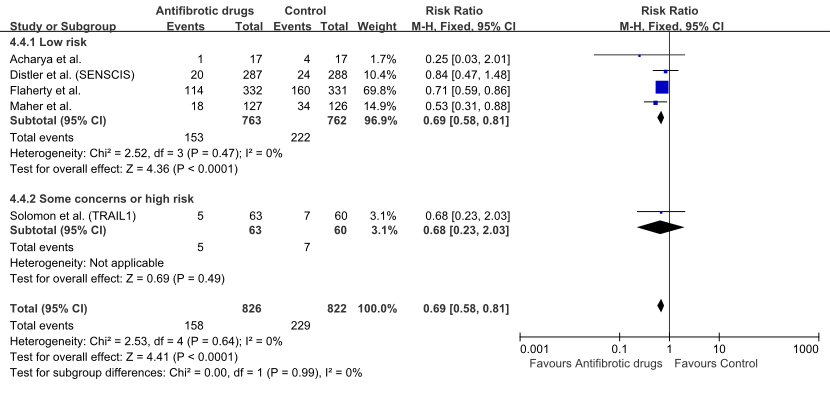


**2.2 TSA for absolute decline in FVC ≥ 10% predicted (trials with low RoB)**


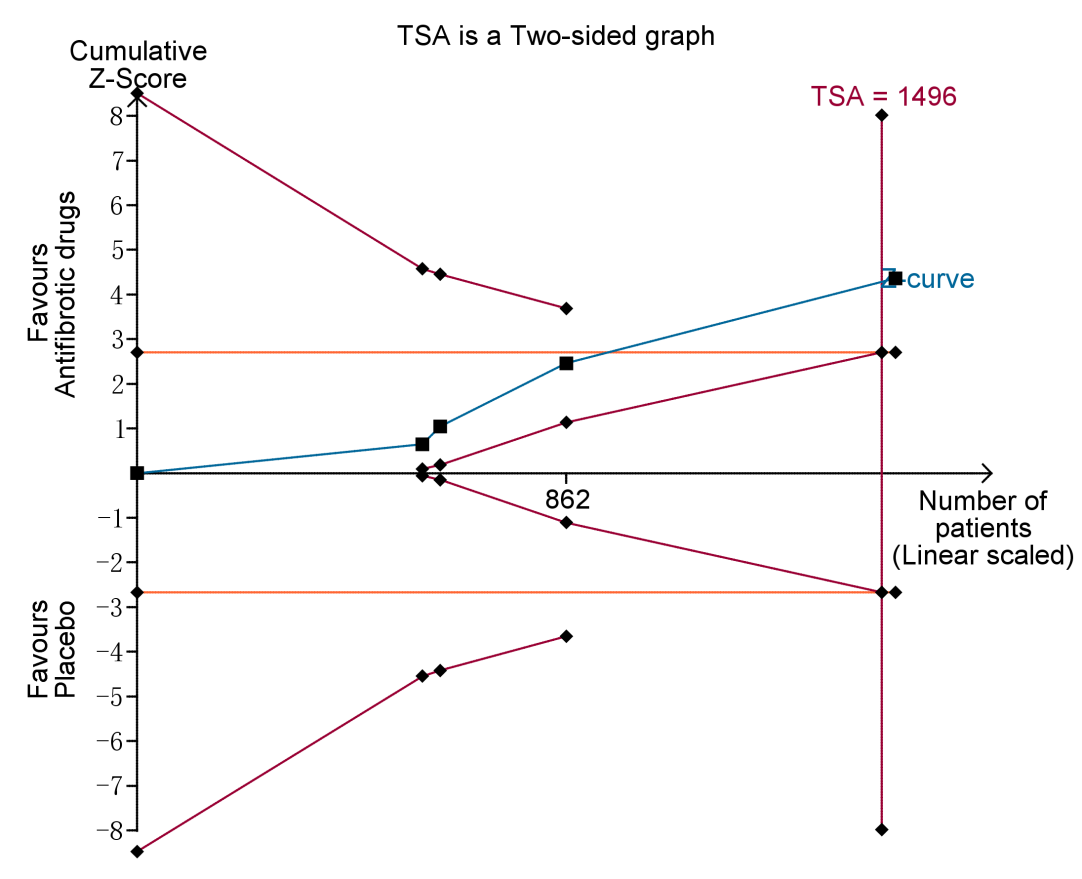


TSA for four trials with low RoB. The RIS was calculated according to α of 0.71%, β of 10%, control event rate of 27.86% (based on all included studies), RRR based on trials with low RoB, model variance-based heterogeneity adjustment. The cumulative z curve did not cross the TSA boundary, but the number of participants reached the RIS of 1,496 patients. Thus the TSA is conclusive, with TSA-adjusted CI of 0.50 to 0.94 (fixed effect model) and a diversity D^2^ of 0%.

**3. Annual rate of decline in FVC (ml/yr), for all participants**

**3.1 Meta-analyses of annual rate of decline in FVC** **(antifibrotic drugs vs. control)**

**
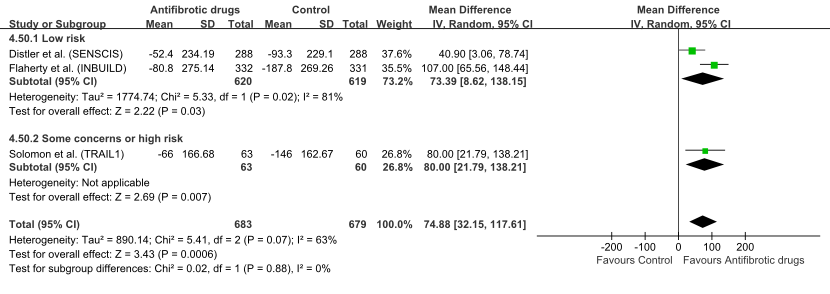
3.2 TSA for annual rate of decline in FVC (trials with low RoB)**


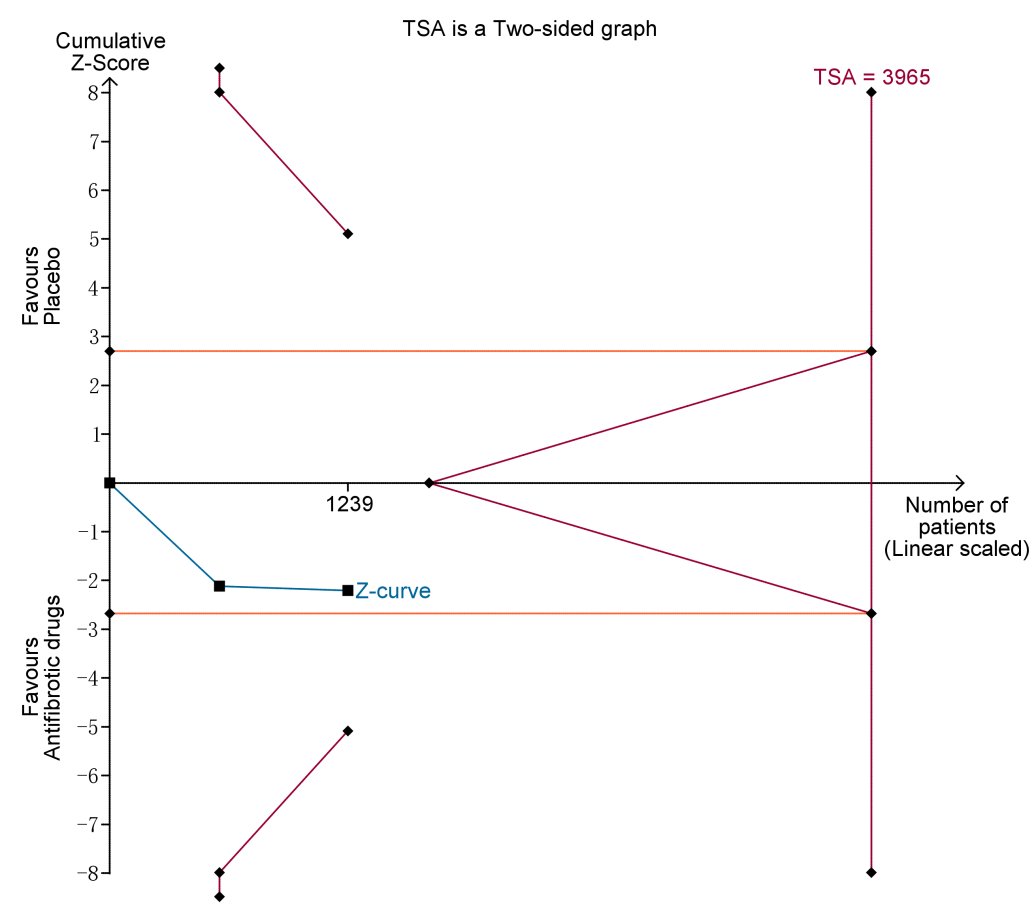


TSA for two trials with low RoB. The RIS was calculated based on mean difference (Empirical), variance (Empirical), α of 0.71% and β of 10%. The cumulative z curve neither crossed the conventional nor the TSA boundary for benefit, harm or futility, with 31.2% of the RIS of 3,965 patients accrued. Thus the TSA is inconclusive, with TSA-adjusted CI of -95.04 to 241.81 (random-effects model) and a diversity D^2^ of 81%.

**4. Annual rate of decline in FVC% predicted, for all participants**

**4.1 Meta-analysis of annual rate of decline in FVC% predicted (antifibrotic drugs vs. control)**

**
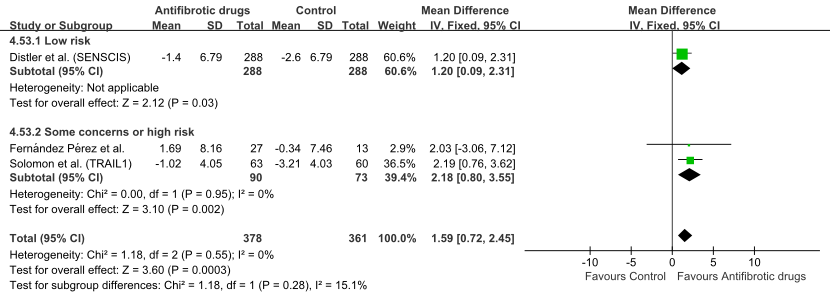
**

**4.2 TSA for annual rate of decline in FVC% predicted (trials with low RoB)**

**
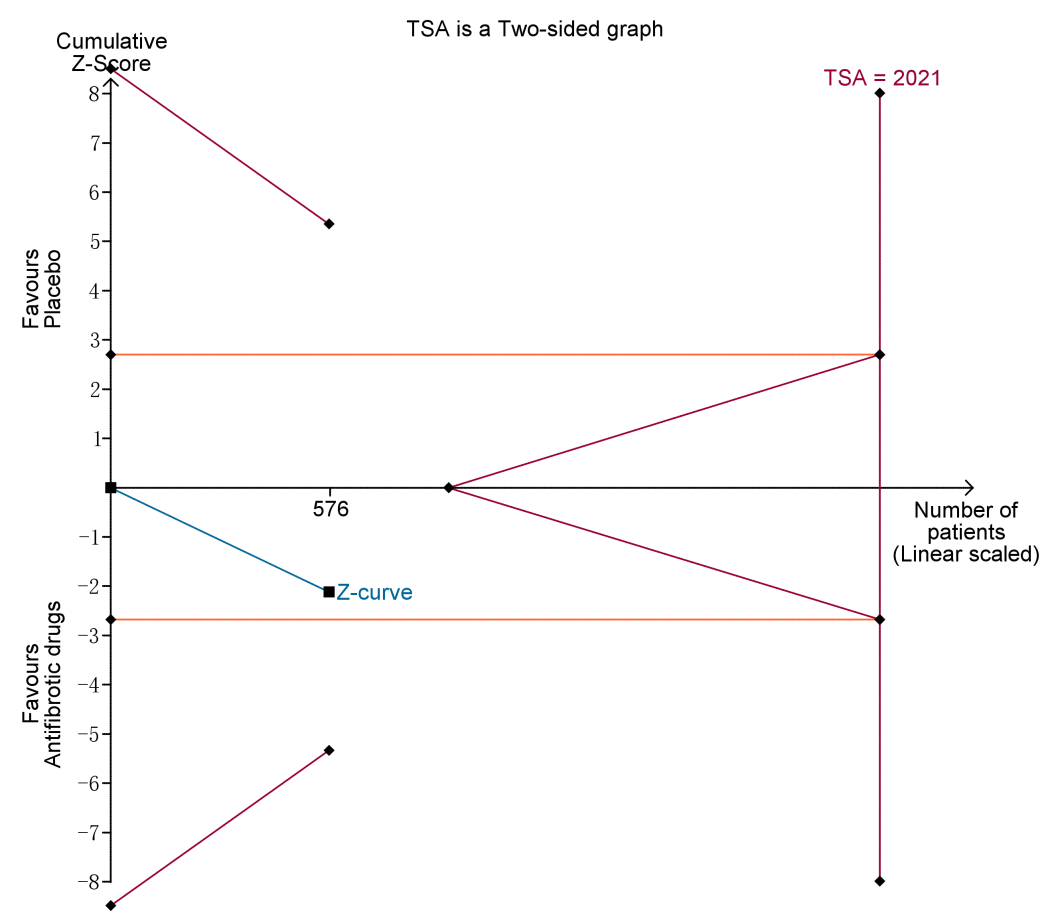
**

TSA for one trial with low RoB. The RIS was calculated based on mean difference (Empirical), variance (Empirical), α of 0.71% and β of 10%. The cumulative z curve neither crossed the conventional nor the TSA boundary for benefit, harm or futility, with 28.5% of the RIS of 2,021 patients accrued. Thus the TSA is inconclusive, with TSA-adjusted CI of -0.75 to 3.15 (fixed effect model) and a diversity D^2^ of 0%.

**5. Absolute change in DLCO% predicted, for all participants**

**5.1 Meta-analyses of absolute change in DLCO% predicted (antifibrotic drugs vs. control)**

**
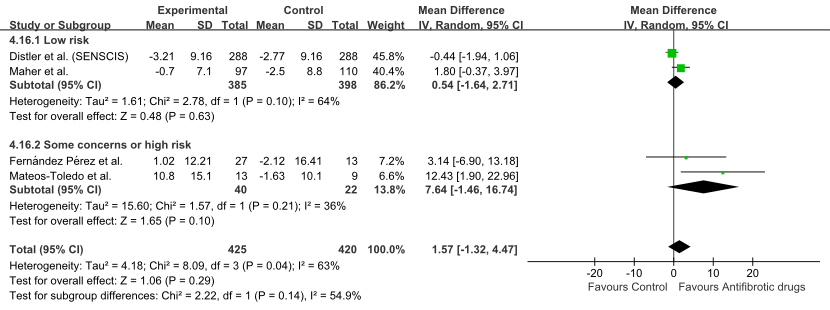
**

**5.2 TSA for absolute change in DLCO% predicted (trials with low RoB)**


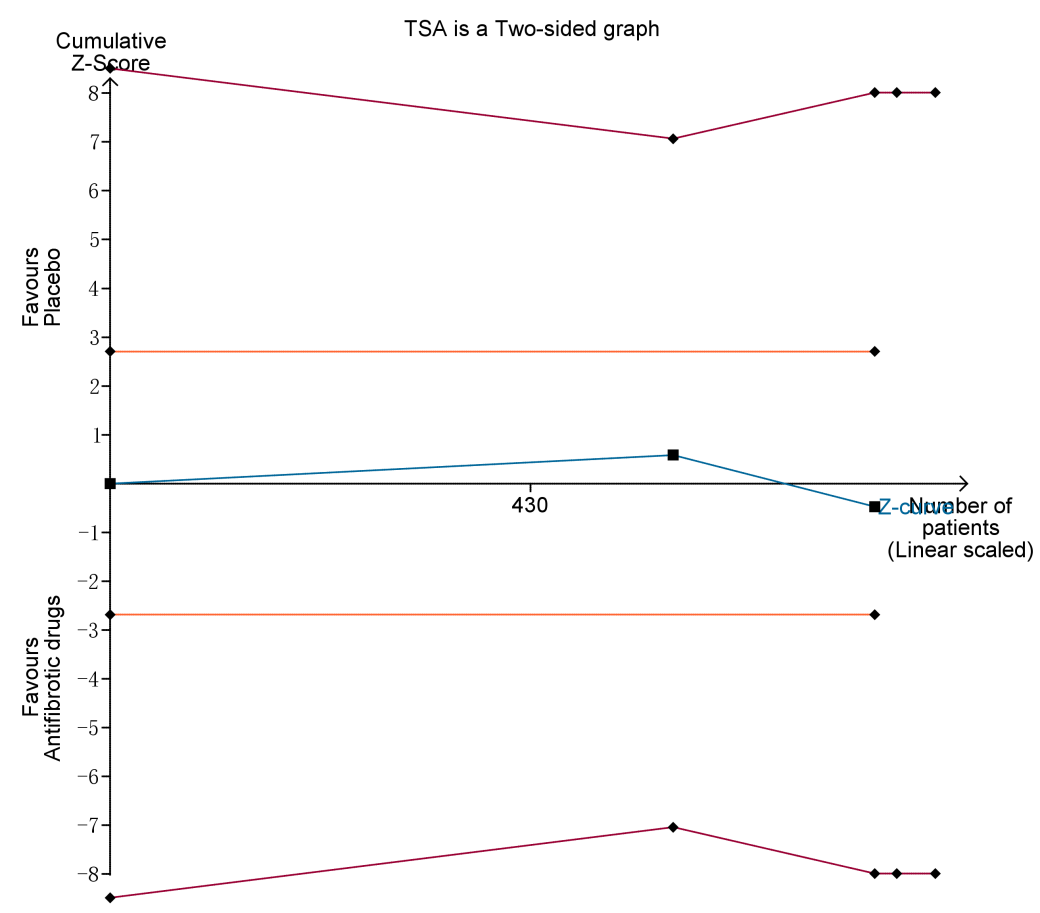


TSA for two trials with low RoB. The RIS was calculated based on mean difference (Empirical), variance (Empirical), α of 0.71% and β of 10%. The cumulative z curve neither crossed the conventional nor the TSA boundary for benefit, harm or futility, with only 1.5% of the RIS of 52,873 patients accrued. Thus the TSA is inconclusive, with a diversity D^2^ of 68%.

**6. Absolute change in 6MWD (m), for all participants**

**6.1 Meta-analysis of absolute change in 6MWD (antifibrotic drugs vs. control)**

**
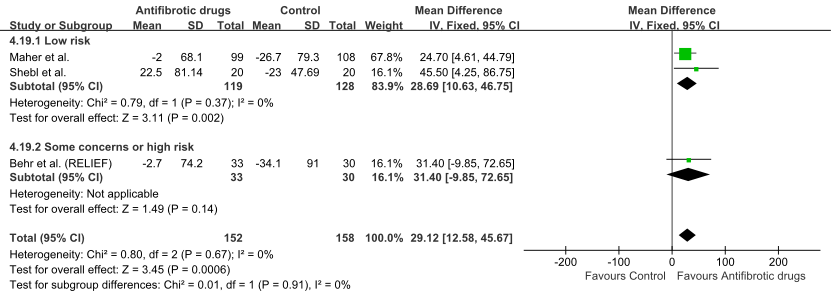
**

**6.2 TSA for absolute change in 6MWD (trials with low ROB)**


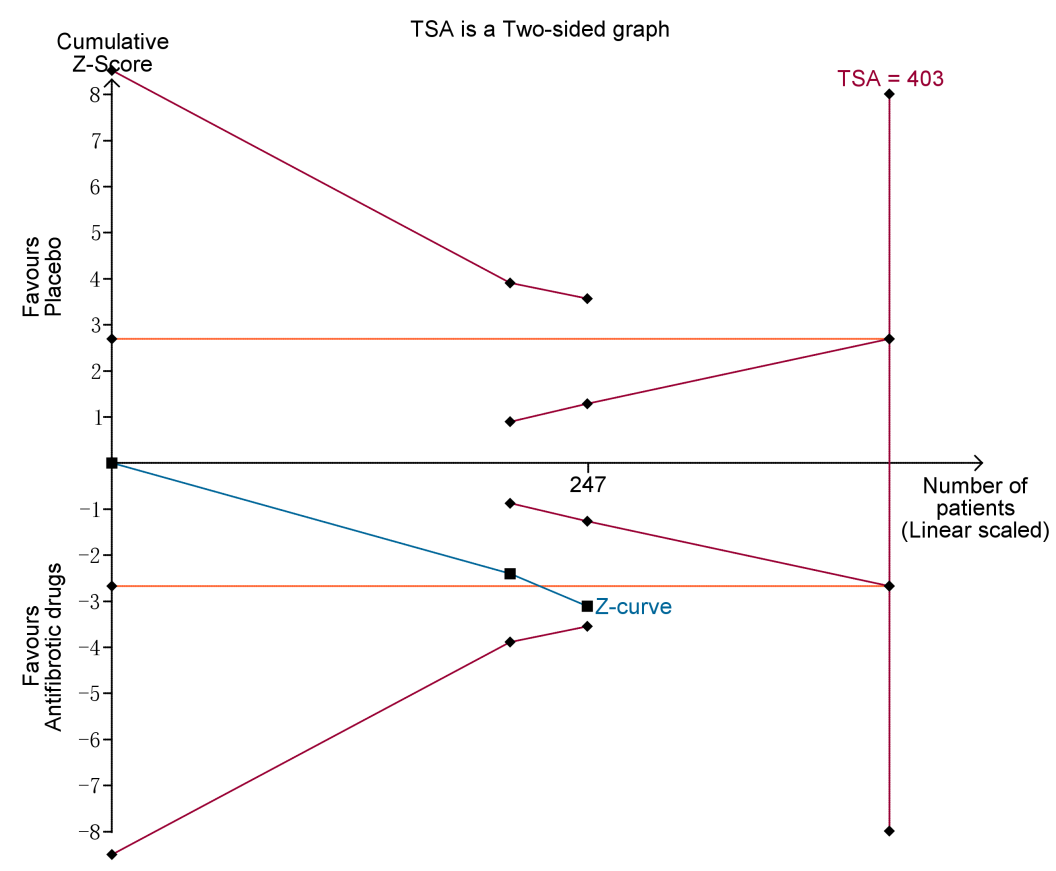


TSA for two trials with low RoB. The RIS was calculated based on mean difference (Empirical), variance (Empirical), α of 0.71% and β of 10%. The cumulative z curve crossed the conventional boundary, but not the TSA boundary for benefit, with 61.3% of the RIS of 403 patients accrued. Thus the TSA is inconclusive, with TSA-adjusted CI of -4.12 to 61.50 (fixed effect model) and a diversity D^2^ of 0%.

**7. Absolute change in SGRQ, for all participants**

**7.1 Meta-analysis of absolute change in SGRQ (antifibrotic drugs vs. control)**

**
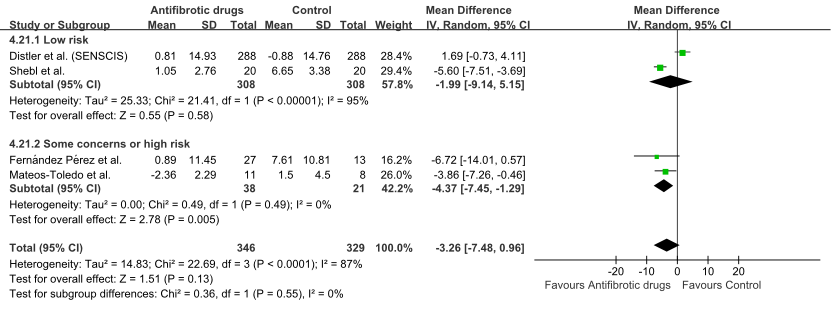
**

**7.2 TSA for absolute change in SGRQ (trials with low ROB)**


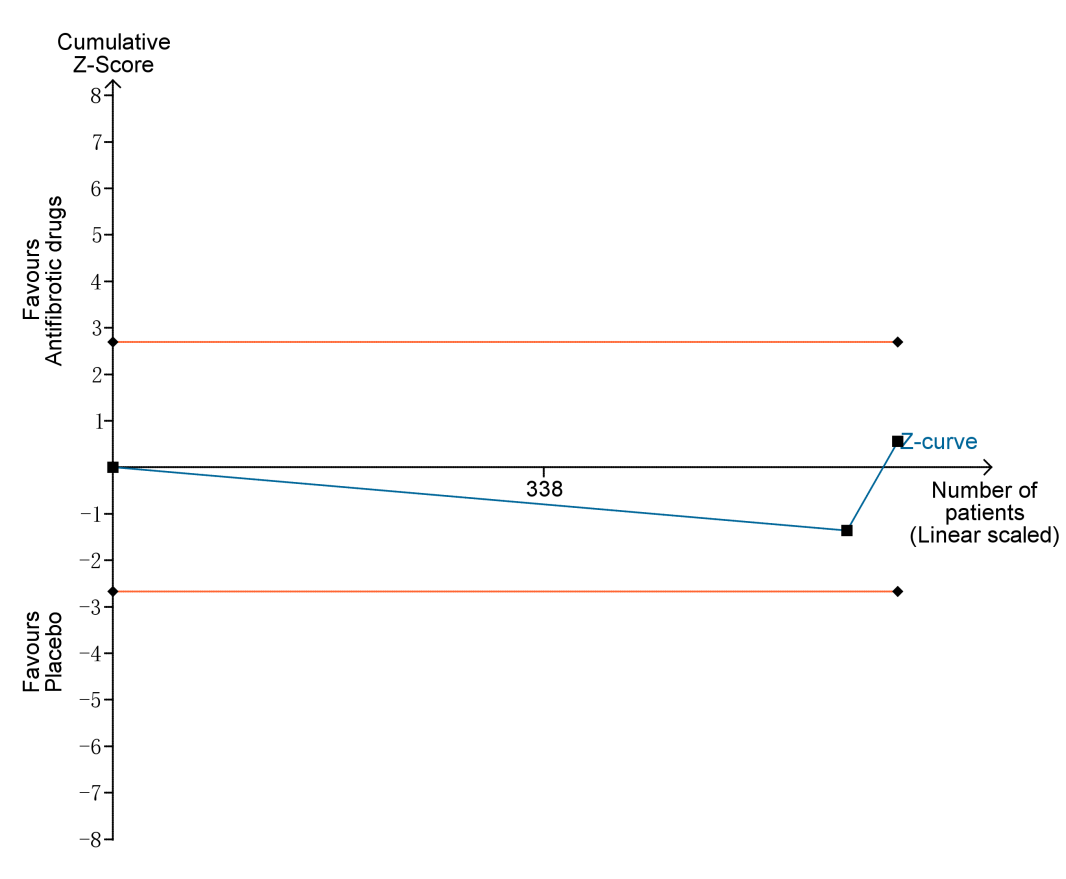


TSA for two trials with low RoB. The RIS was calculated based on mean difference (Empirical), variance (Empirical), α of 0.71% and β of 10%. The cumulative z curve neither crossed the conventional nor the TSA boundary for benefit, harm or futility, with only 1.9% of the RIS of 32,455 patients accrued. Thus the TSA is inconclusive, with a diversity D^2^ of 96%.

**8. Acute exacerbation of ILD, for all participants**

**8.1 Meta-analysis of acute exacerbation of ILD (antifibrotic drugs vs. control)**

**
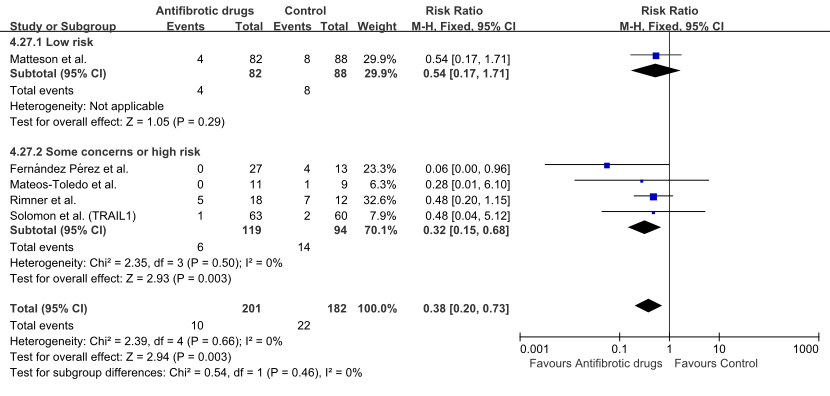
**

**8.2 TSA for acute exacerbation of ILD (trial with low RoB)**


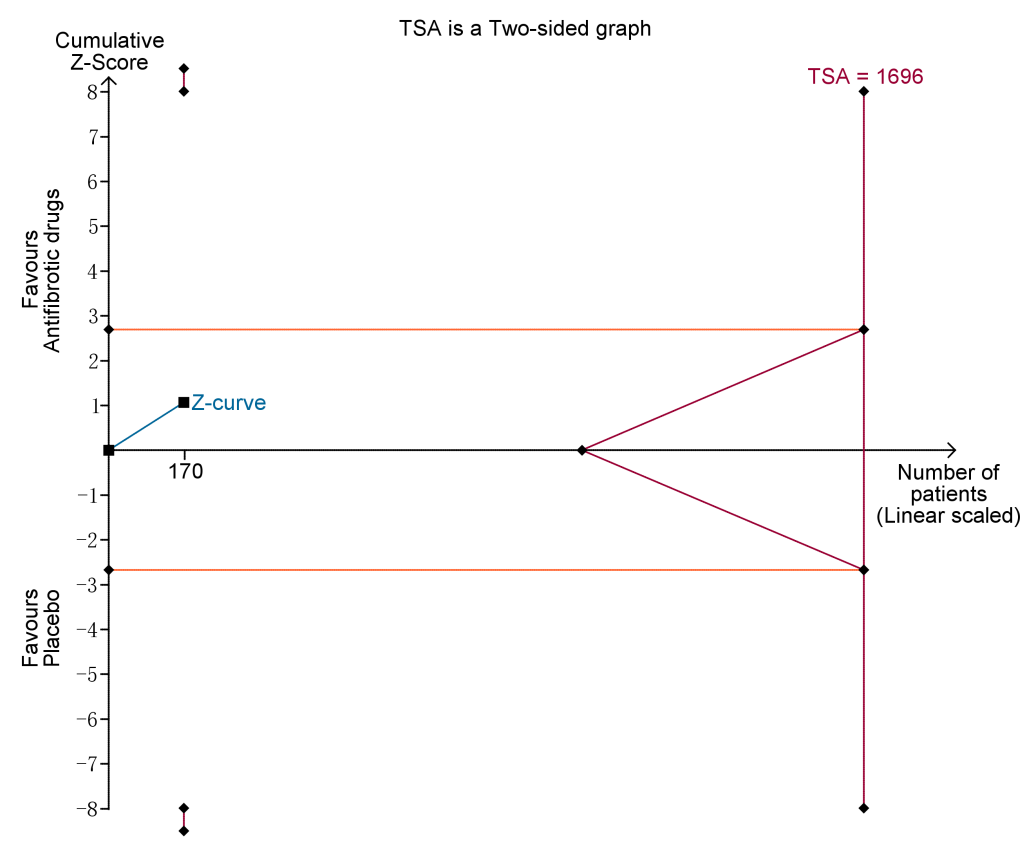


TSA for one trial with low RoB. The RIS was calculated according to α of 0.71%, β of 10%, control event rate of 12.1% (based on all included studies), RRR based on trials with low RoB, model variance-based heterogeneity adjustment. The cumulative z curve neither crossed the conventional nor the TSA boundary for benefit, harm or futility, with 10% of the RIS of 1,696 patients accrued. Thus the TSA is inconclusive, with TSA-adjusted CI of 0.00 to 61.56 (fixed effect model) and a diversity D^2^ of 0%.

**9. AE: diarrhea, for all participants**

**9.1 Meta-analysis of diarrhea (antifibrotic drugs vs. control)**

**
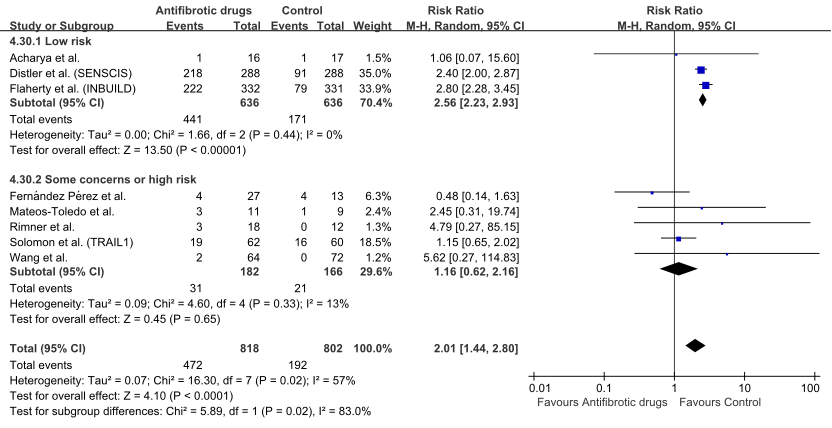
**

**9.2 TSA for diarrhea (trials with low RoB)**


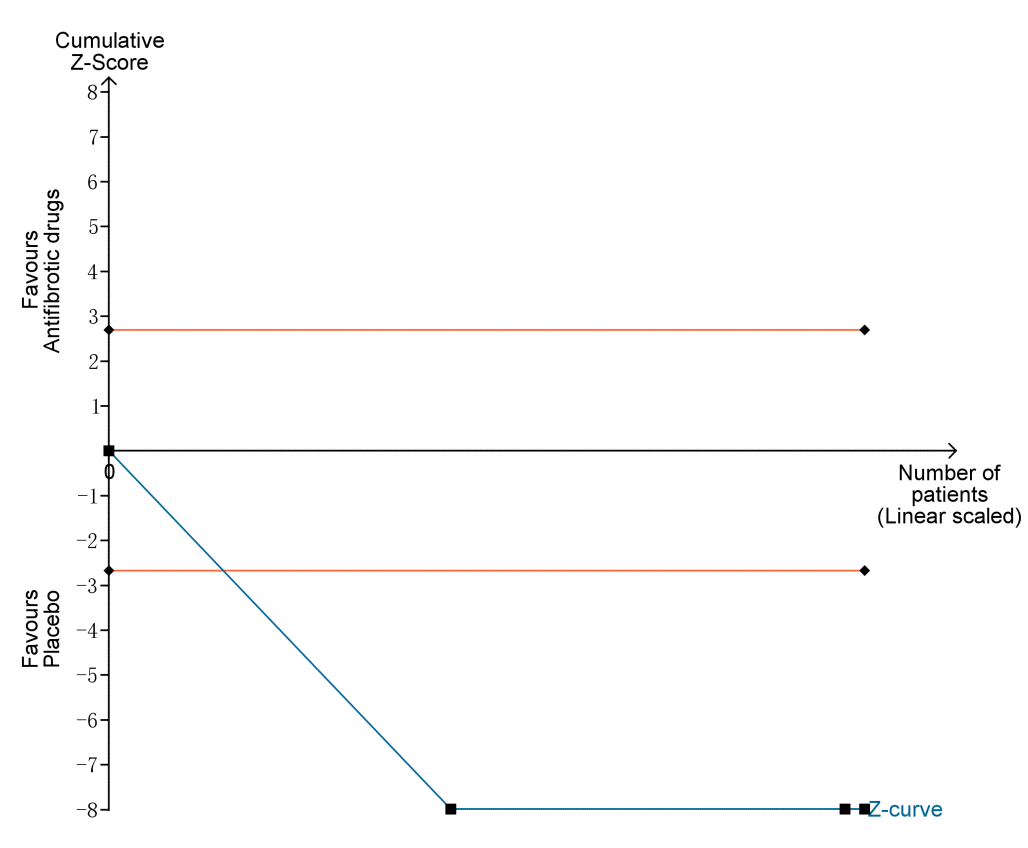


TSA for three trials with low RoB. The RIS was calculated according to α of 0.71%, β of 10%, control event rate of 23.94% (based on all included studies), RRR based on trials with low RoB, model variance-based heterogeneity adjustment. TSA suggested that the RIS of 109 patients was reached within the first trial. Thus the TSA is conclusive, with a diversity D^2^ of 0%.

**10. AE: nausea, for all participants**

**10.1 Meta-analysis of nausea (antifibrotic drugs vs. control)**

**
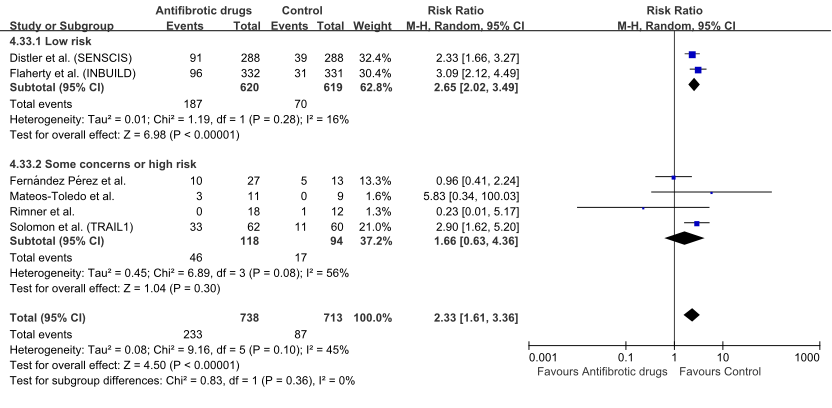
**

**10.2 TSA for nausea (trials with low RoB)**


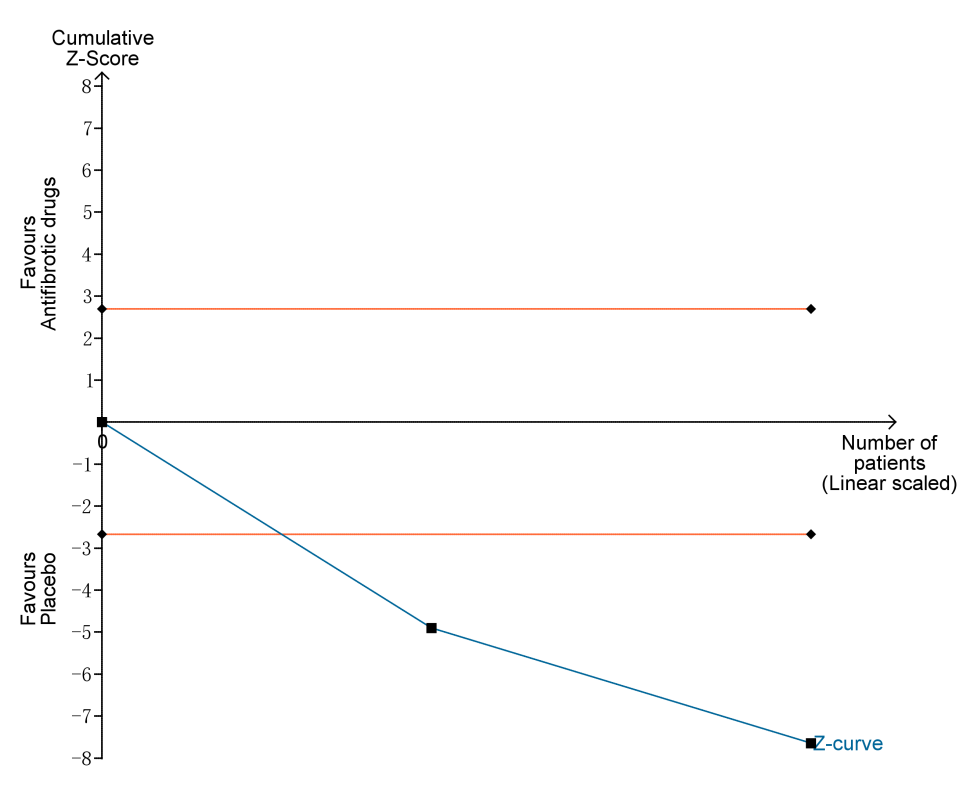


TSA for two trials with low RoB. The RIS was calculated according to α of 0.71%, β of 10%, control event rate of 12.2% (based on all included studies), RRR based on trials with low RoB, model variance-based heterogeneity adjustment. TSA suggested that the RIS of 315 patients was reached within the first trial. Thus the TSA is conclusive, with a diversity D^2^ of 16%.

**11. AE: vomiting, for all participants**

**11.1 Meta-analysis of vomiting (antifibrotic drugs vs. control)**

**
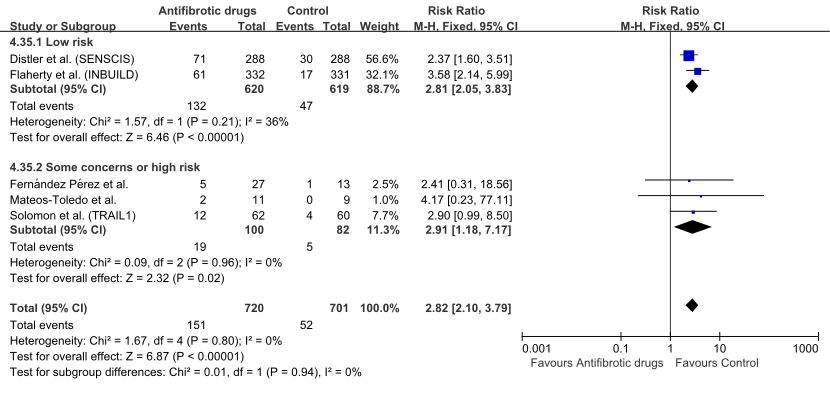
**

**11.2 TSA for vomiting (trials with low RoB)**


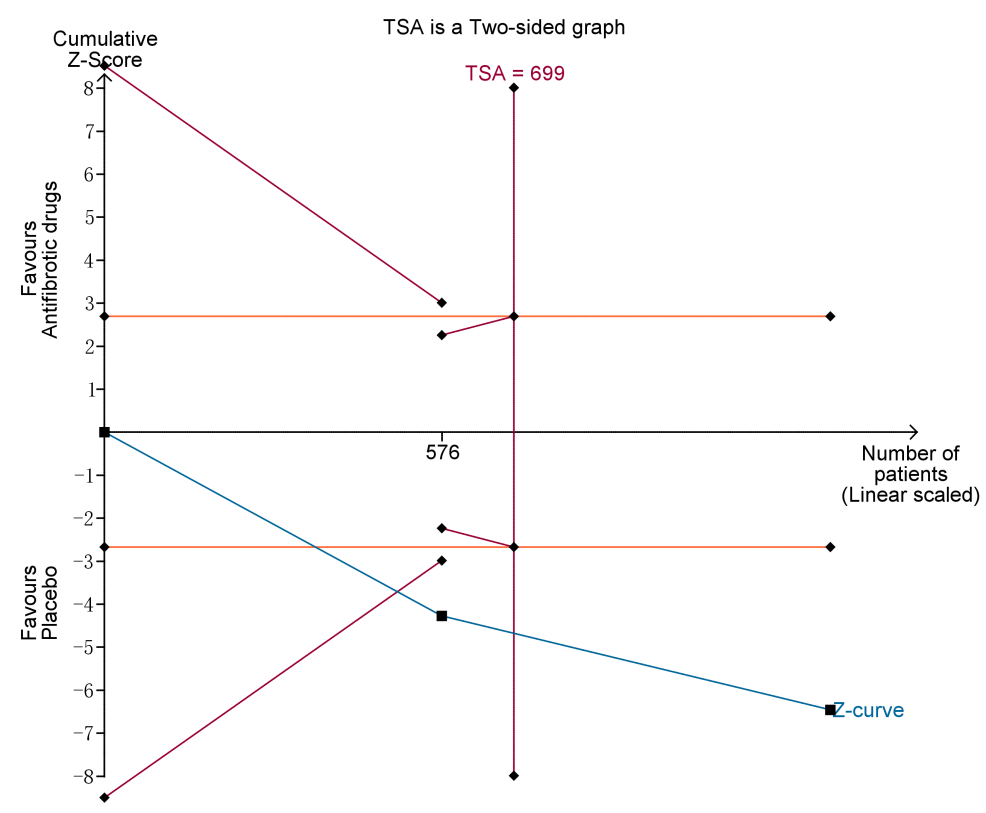


TSA for two trials with low RoB. The RIS was calculated according to α of 0.71%, β of 10%, control event rate of 7.4% (based on all included studies), RRR based on trials with low RoB, model variance-based heterogeneity adjustment. The cumulative z curve crossed the TSA boundary for harm, with more than the RIS of 699 patients accrued. Thus the TSA is conclusive, with TSA-adjusted CI of 1.74 to 4.53 (fixed effect model) and a diversity D^2^ of 39%.

**12. AE: elevation of transaminases, for all participants**

**12.1 Meta analysis of elevation of transaminases (antifibrotic drugs vs. control)**

**
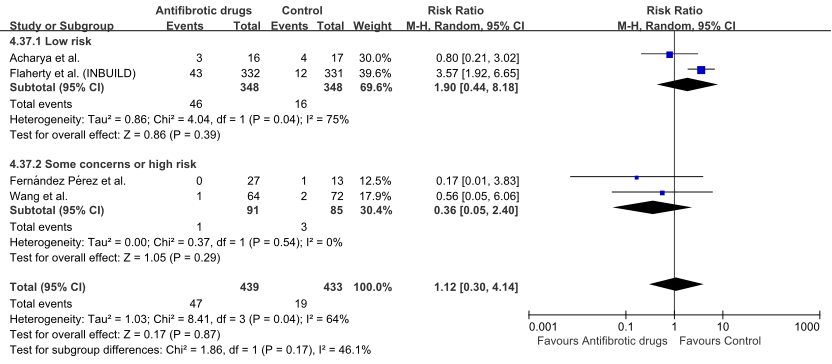
**

**12.2 TSA for elevation of transaminases (trials with low RoB)**


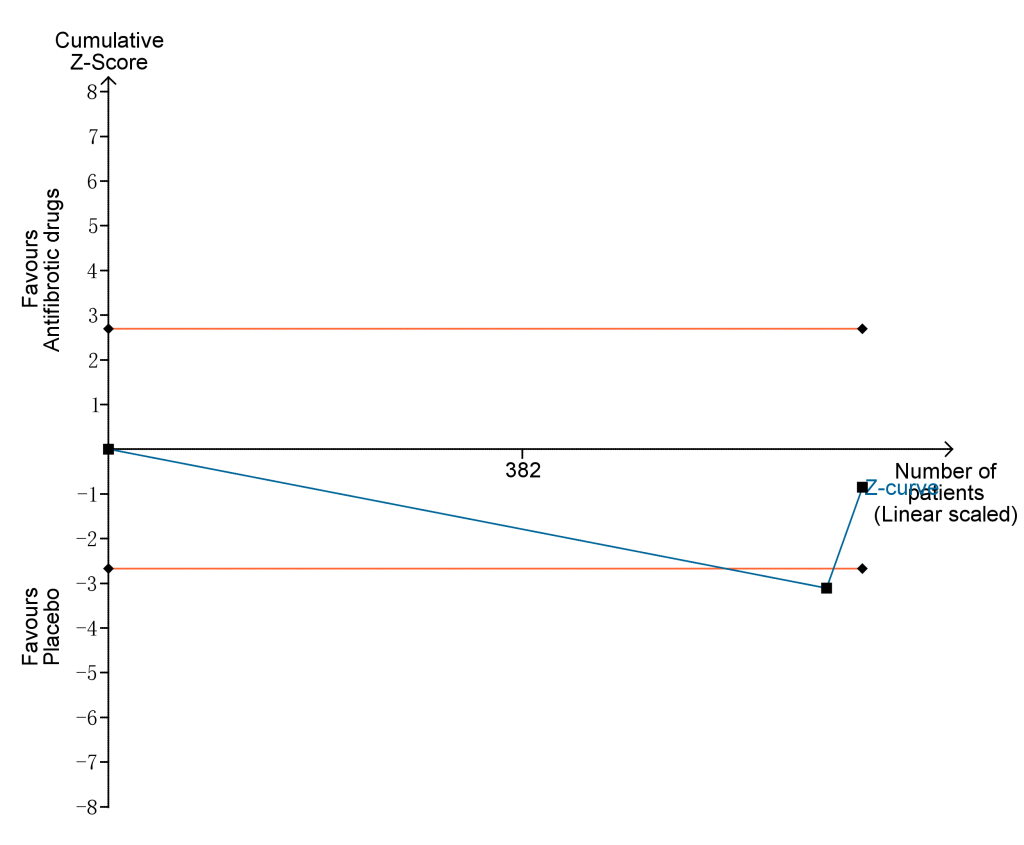


TSA for two trials with low RoB. The RIS was calculated according to α of 0.71%, β of 10%, control event rate of 4.4% (based on all included studies), RRR based on trials with low RoB, model variance-based heterogeneity adjustment. The cumulative z curve neither crossed the conventional nor the TSA boundary for benefit, harm, or futility, with only 4.3% of the RIS of 16,103 patients accrued. Thus the TSA is inconclusive, with a diversity D^2^ of 85%.

**13. AEs leading to treatment discontinuation, for all participants**

**13.1 Meta-analysis of AEs leading to treatment discontinuation (antifibrotic drugs vs. control)**

**
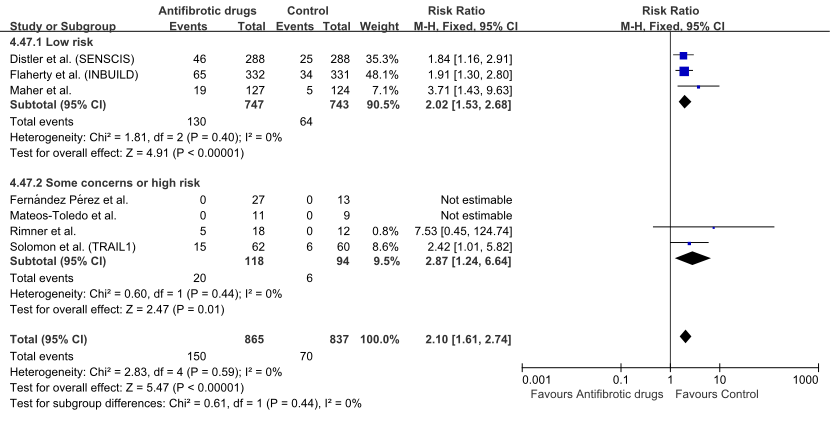
**

**13.2 TSA for AEs leading to treatment discontinuation (trials with low RoB)**

**
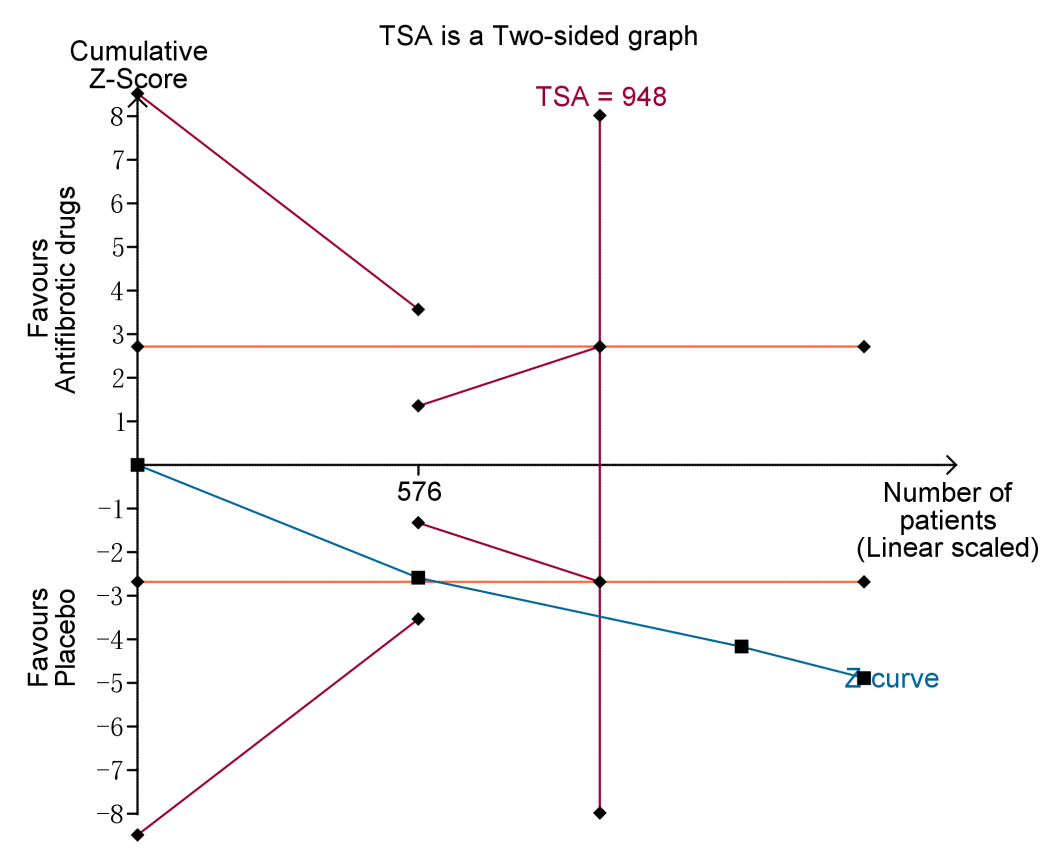
**

TSA for three trials with low RoB. The RIS was calculated according to α of 0.71%, β of 10%, control event rate of 8.4% (based on all included studies), RRR based on trials with low RoB, model variance-based heterogeneity adjustment. The cumulative z curve did not cross the TSA boundary for harm, but the accrued sample size reached the RIS of 948 participants. Thus the TSA is conclusive, with TSA-adjusted CI of 1.21 to 3.37 (fixed effect model) and a diversity D^2^ of 0%.

**S8. Analyses of exploratory outcome**

Exploratory outcome was not analyzed in patients with a progressive fibrosing phenotype due to sparse data.

**1. Meta-analysis of respiratory-related mortality, for all participants**

**
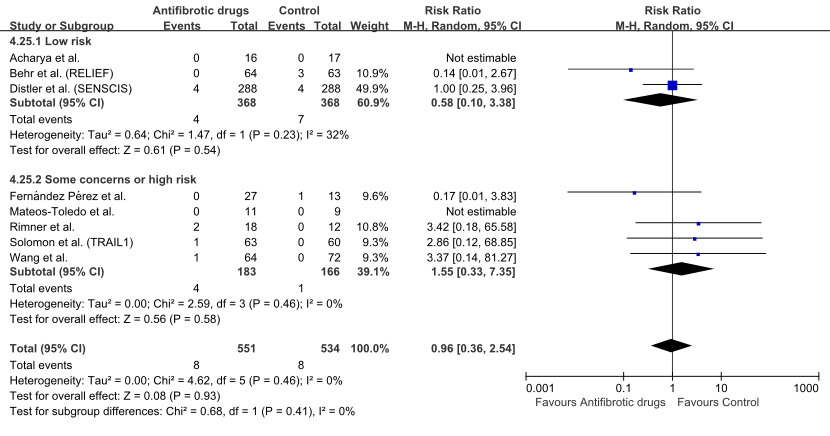
**

**2. TSA for respiratory-related mortality (trials with low RoB)**


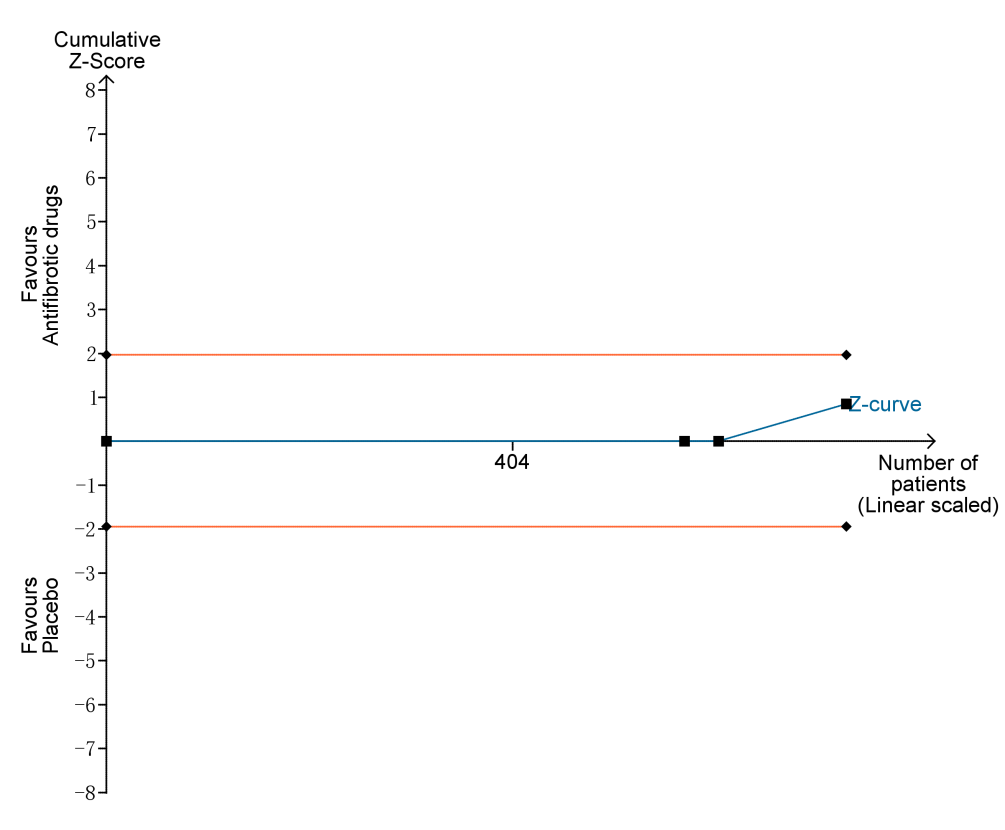


TSA for three trials with low RoB. The RIS was calculated according to α of 5%, β of 10%, control event rate of 1.5% (based on all included studies), RRR based on trials with low RoB, model variance-based heterogeneity adjustment. The cumulative z curve neither crossed the conventional nor the TSA boundary for benefit, harm, or futility, with only 4.6% of the RIS of 16,152 patients accrued. Thus the TSA is inconclusive, with a diversity D^2^ of 0%.

**S9. Subgroup analyses**

**1. Subgroup analyses in all participants**

1) Because of limited data, not all the outcome measures were available in predefined subgroups.

2) The forest plots for primary outcomes were shown below. The summary of subgroup analyses for secondary and exploratory outcomes were presented in **Table S2**.

**Forest plots of subgroup analyses for primary outcomes**

**1.1 Absolute change in FVC (ml)**

**1.1.1 Absolute change in FVC: low RoB vs some concerns or high RoB**

**
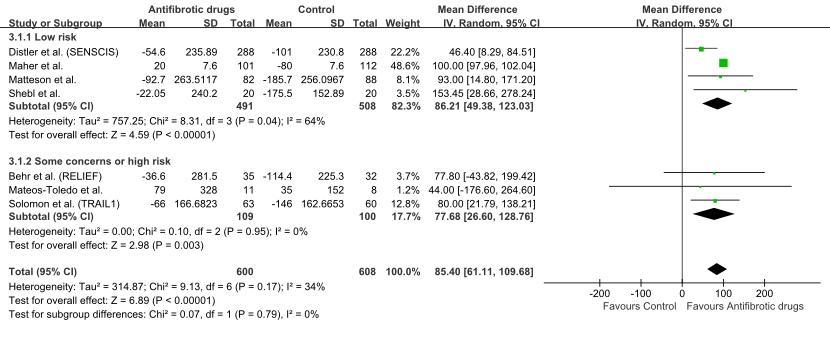
**

**1.1.2 Absolute change in FVC: pirfenidone vs nintedanib**

**
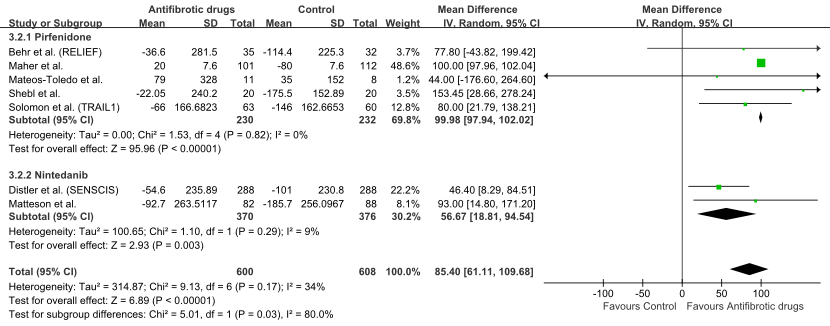
**

**1.1.3 Absolute change in FVC:＜12 months vs ≥12 months of follow up**

**
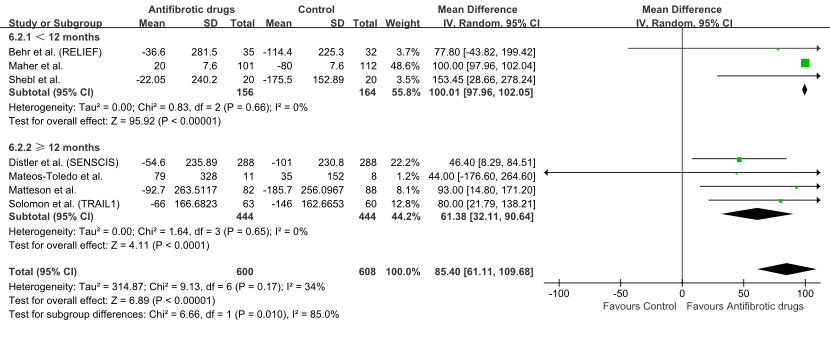
**

**1.1.4 Absolute change in FVC: AID-ILD vs hypersensitivity pneumonitis vs unclassifiable ILD**

**
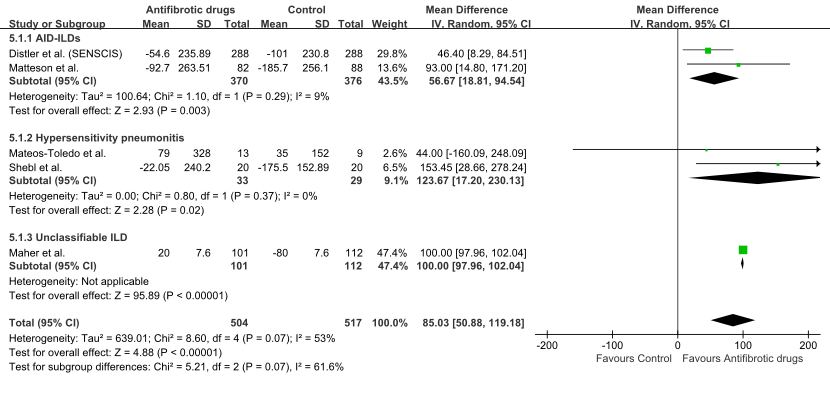
**

**1.1.5 Absolute change in FVC: taking vs not taking mycophenolate at baseline**

**
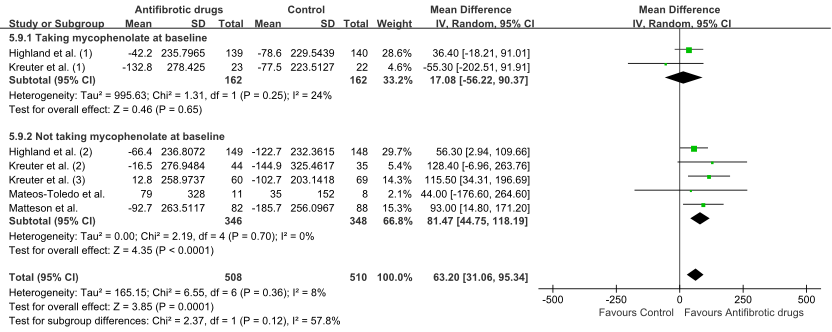
**

**1.1.6 TSA for absolute change in FVC in patients taking mycophenolate at baseline (trials with low RoB)**

**
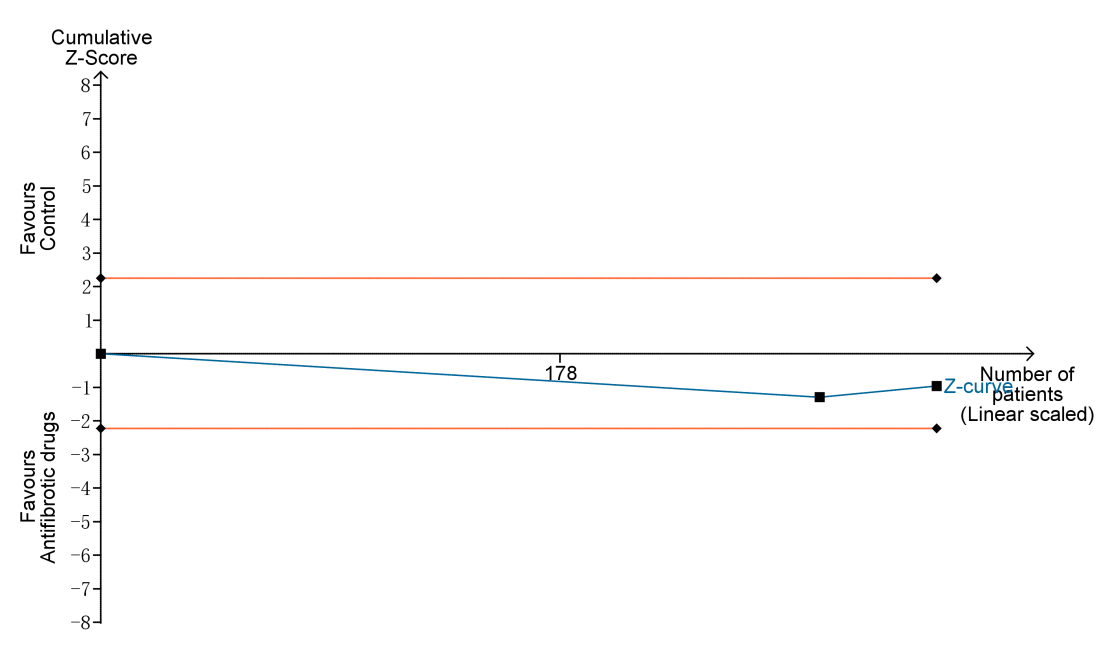
**

TSA for two trials with low RoB. The RIS was calculated based on mean difference (Empirical), variance (Empirical), α of 2.5% and β of 10%. The cumulative z curve neither crossed the conventional nor the TSA boundary for benefit, harm or futility, with only 3.7% of the RIS of 8,781 patients accrued. Thus the TSA is inconclusive, with a diversity D^2^ of 51%.

**1.2 All-cause mortality**

**1.2.1 All-cause mortality: low RoB vs some concerns or high RoB**

**
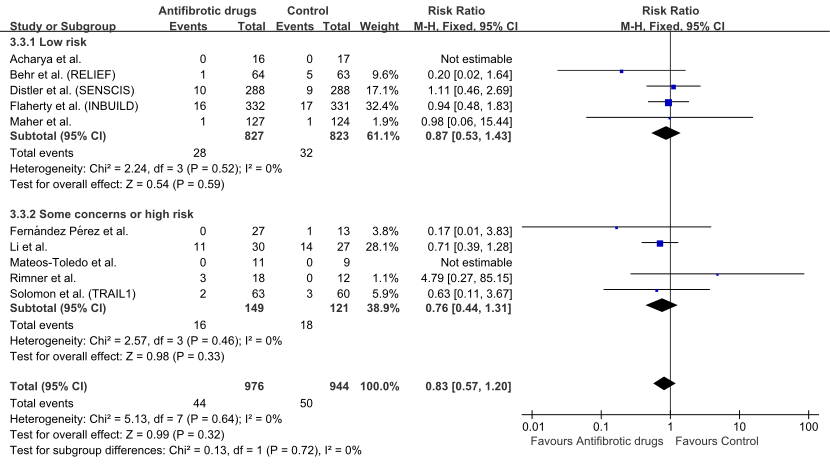
**

**1.2.2 All-cause mortality: pirfenidone vs nintedanib**

**
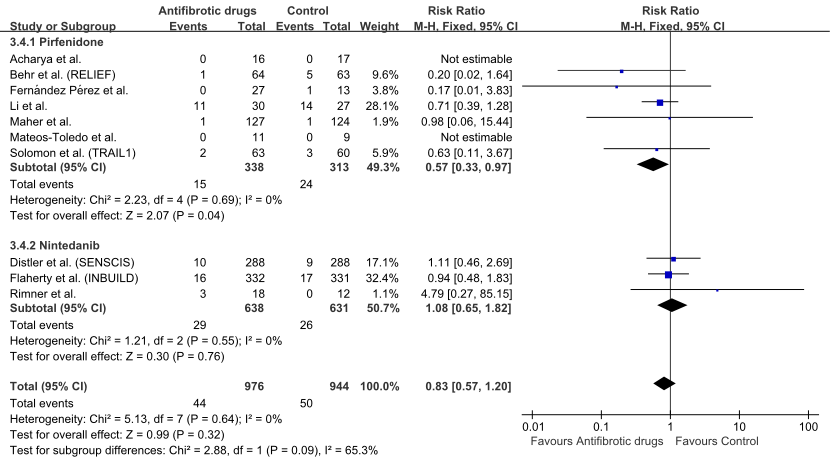
**

**1.2.3 All-cause mortality:＜12 months vs ≥ 12 months of follow-up**

**
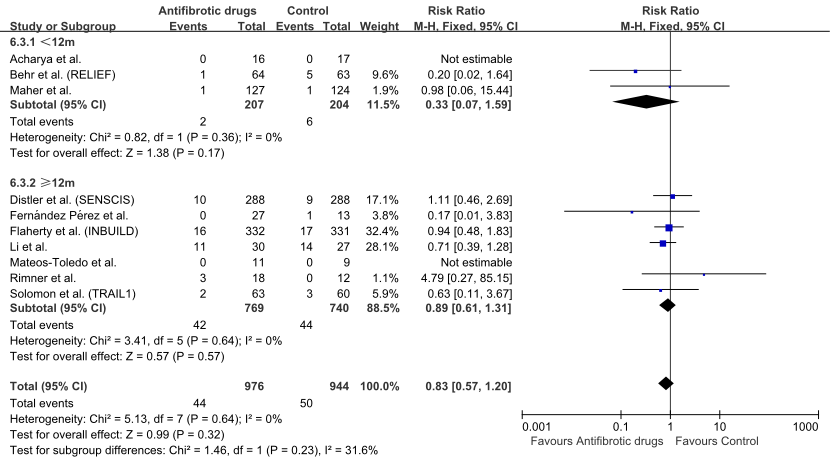
**

**1.2.4 All-cause mortality: AID-ILD vs hypersensitivity pneumonitis vs radiation pneumonitis vs unclassifiable ILD**

**
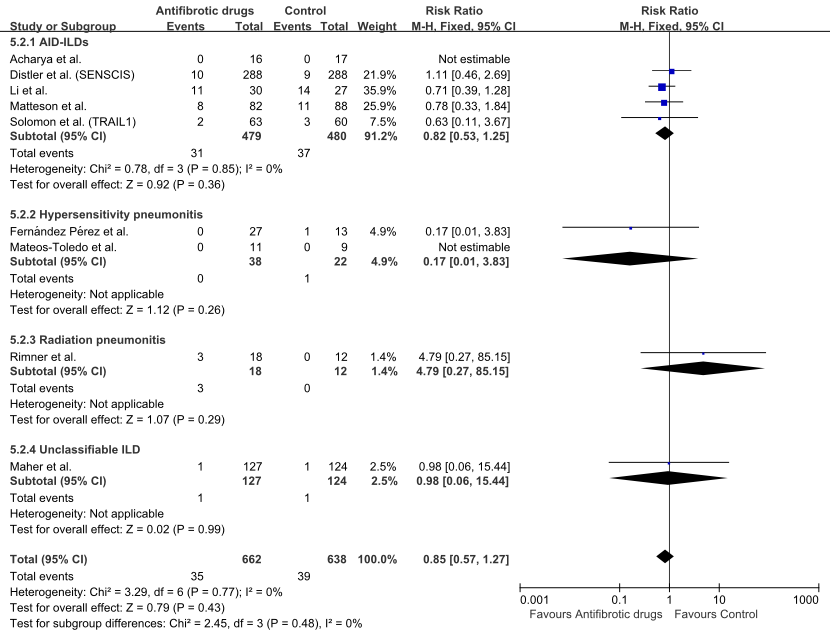
**

**1.2.5 All-cause mortality: taking vs not taking mycophenolate at baseline**

**
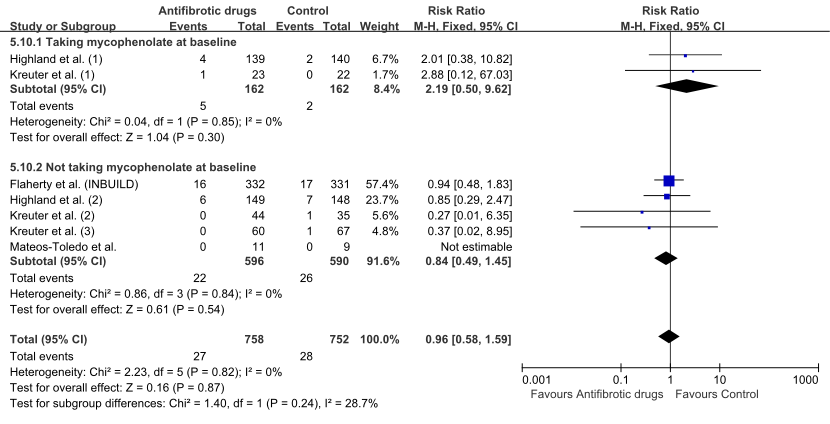
**

**1.2.6 TSA for all-cause mortality in patients taking mycophenolate at baseline**


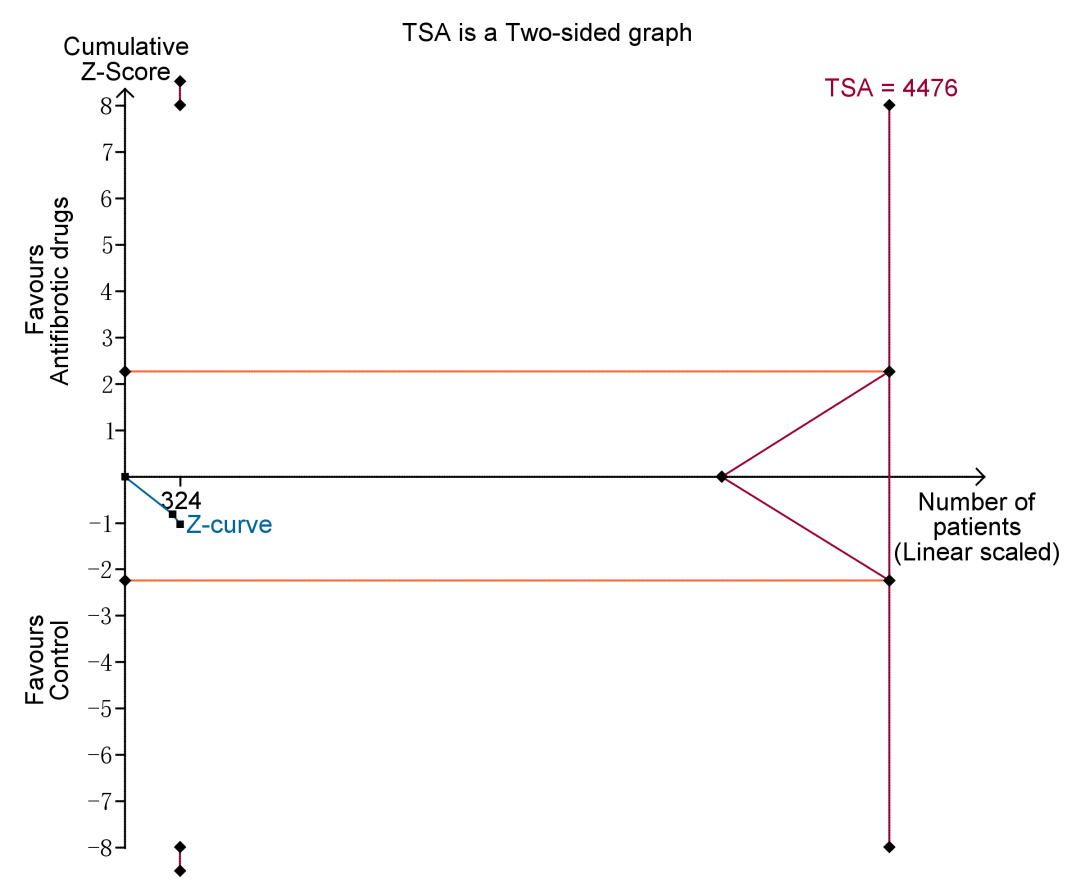


TSA for two trials with low RoB. The RIS was calculated according to α of 2.5%, β of 10%, control event rate of 1.2% (based on all included studies), RRR based on trials with low RoB, model variance-based heterogeneity adjustment. The cumulative z curve neither crossed the conventional nor the TSA boundary for benefit, harm or futility, with only 7.2% of the RIS of 4,476 patients accrued. Thus the TSA is inconclusive, with TSA-adjusted CI of 0.01 to 918.42 and a diversity D^2^ of 0%.

**1.2.7 All-cause mortality: UIP vs non-UIP**


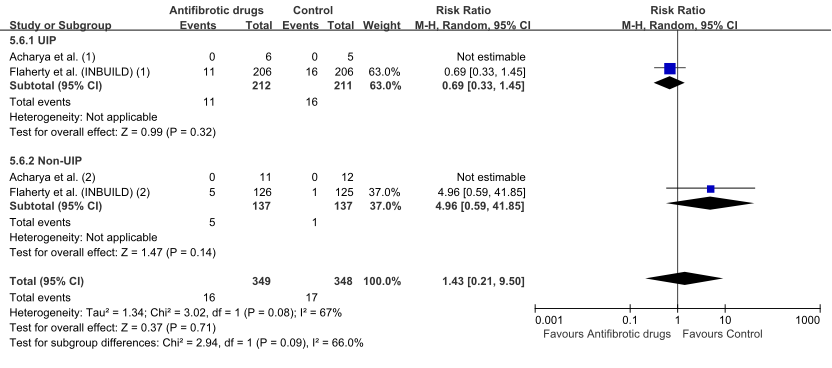


**1.3 SAEs**

**1.3.1 SAEs: low RoB vs some concerns or high RoB**

**
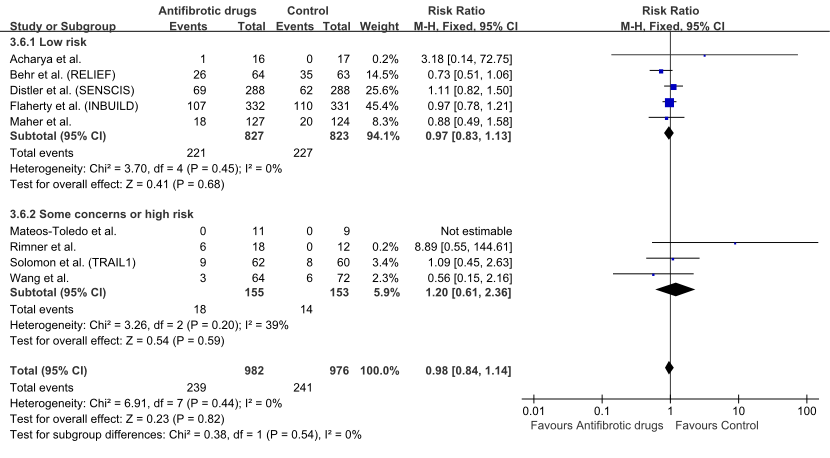
**

**1.3.2 SAEs: pirfenidone vs nintedanib**

**
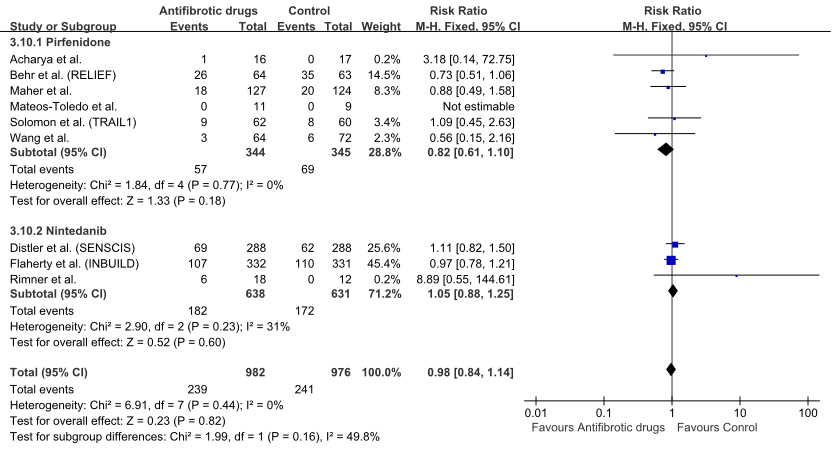
**

**1.3.3 SAEs:＜12 months vs ≥ 12 months of follow-up**


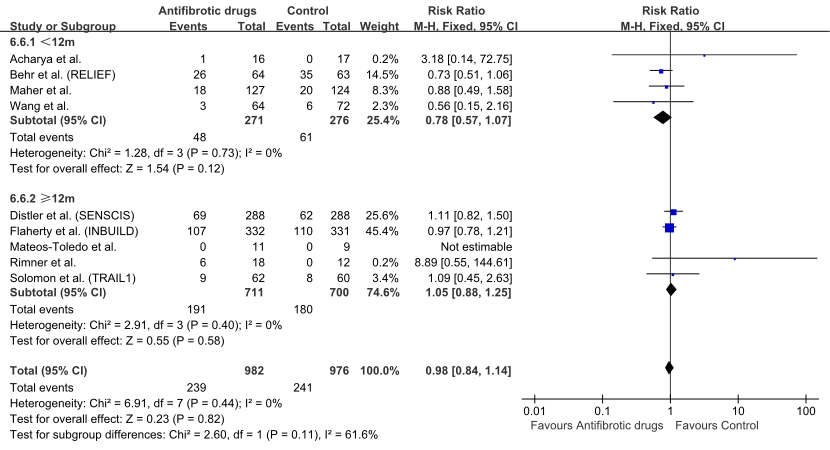


**1.3.4 SAEs: AID-ILD vs hypersensitivity pneumonitis vs unclassifiable ILD vs iNSIP vs other ILDs**

**
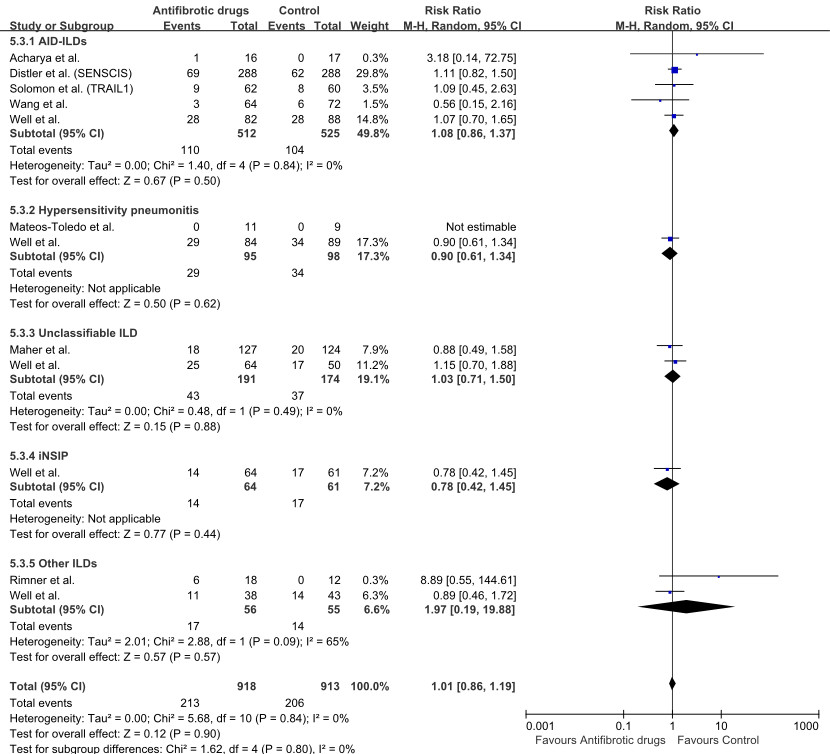
**

**1.3.5 SAEs: taking vs not taking mycophenolate at baseline**

**
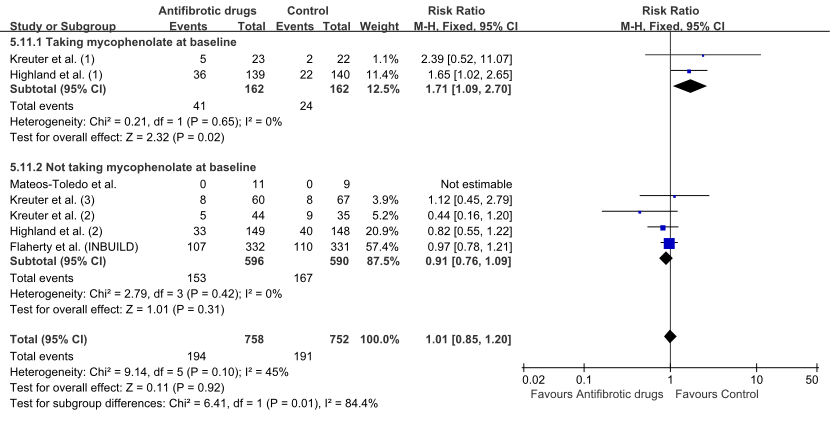
**

**1.3.6 TSA for SAEs in patients taking mycophenolate at baseline**

**
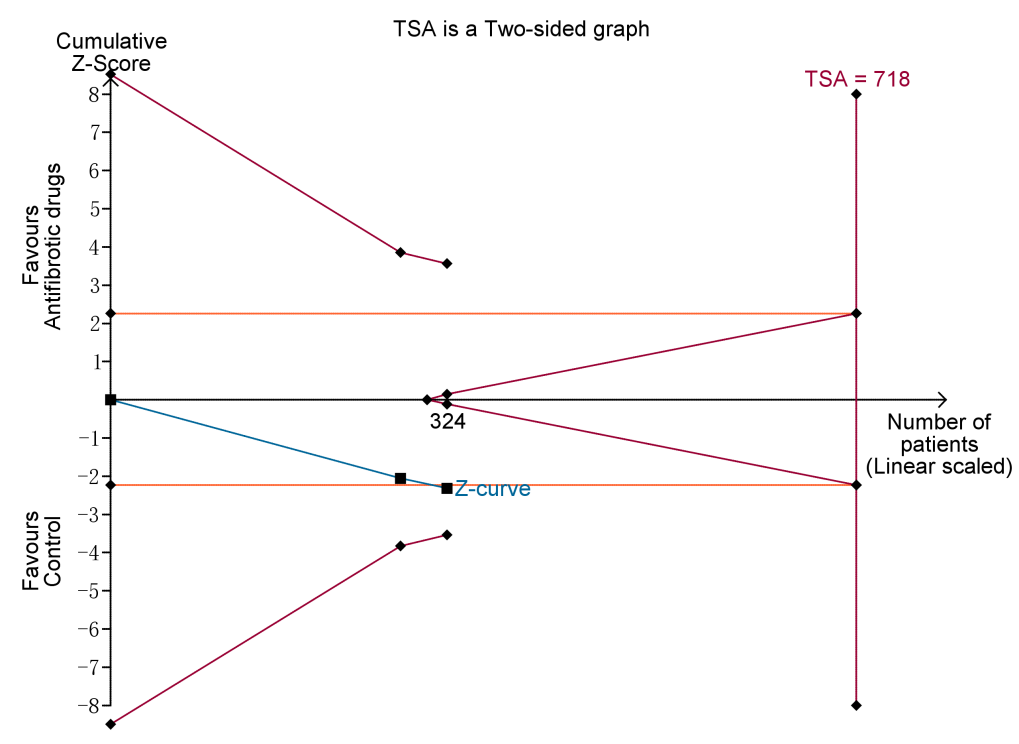
**

TSA for two trials with low RoB. The RIS was calculated according to α of 2.5%, β of 10%, control event rate of 14.8% (based on all included studies), RRR based on trials with low RoB, model variance-based heterogeneity adjustment. The cumulative z curve crossed the conventional boundary but not the TSA boundary for harm, with 45.1% of the RIS of 718 patients accrued. Thus the TSA is inconclusive, with TSA-adjusted CI of 0.75 to 3.91 and a diversity D^2^ of 0%.

**2. Subgroup analyses in patients with a progressive fibrosing phenotype**

On basis of currently available data, we could only analyze the following outcomes and subgroups in patients with progressive fibrosing phenotype.

**2.1 Absolute change in FVC (ml)**

**2.1.1 Absolute change in FVC: low RoB vs some concerns or high RoB**

**
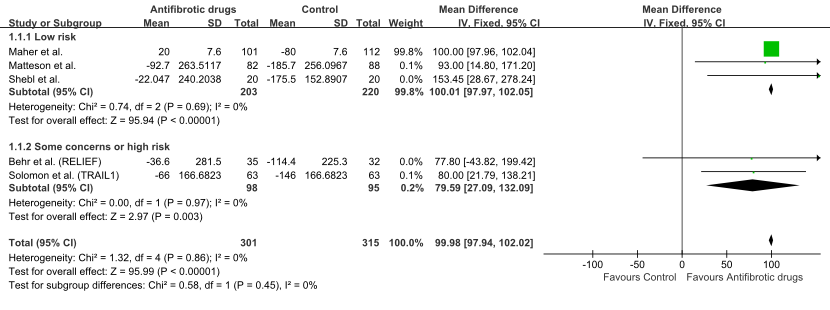
**

**2.1.2 Absolute change in FVC: pirfenidone vs nintedanib**

**
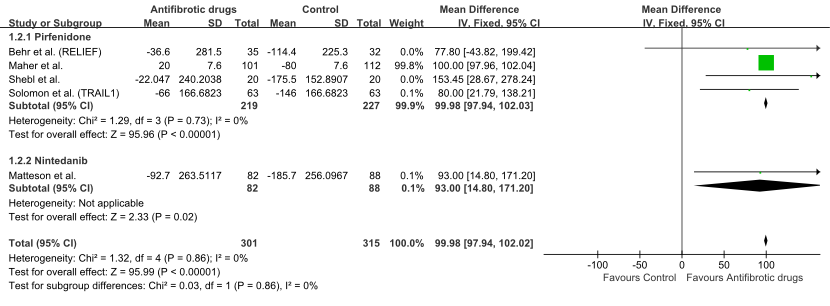
**

**2.1.3 Absolute change in FVC: ＜12 months vs ≥12 months of follow up**

**
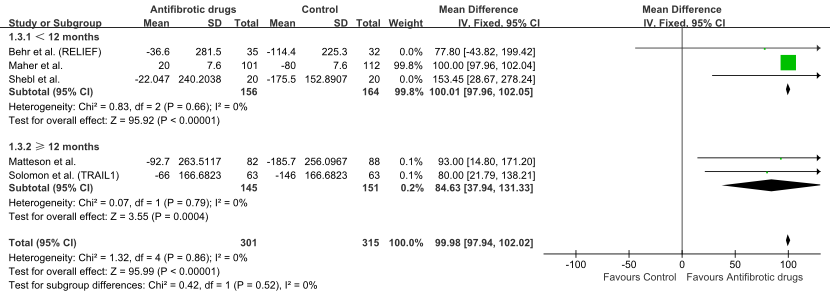
**

**2.1.4 Absolute change in FVC: taking vs not taking mycophenolate at baseline**


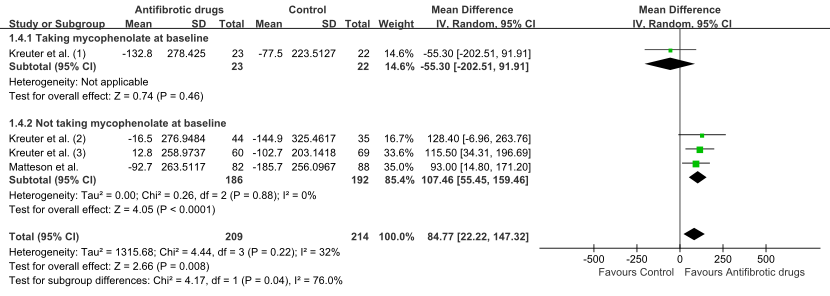


**2.2 All-cause mortality**

**2.2.1 All-cause mortality: low RoB vs some concerns or high RoB**

**
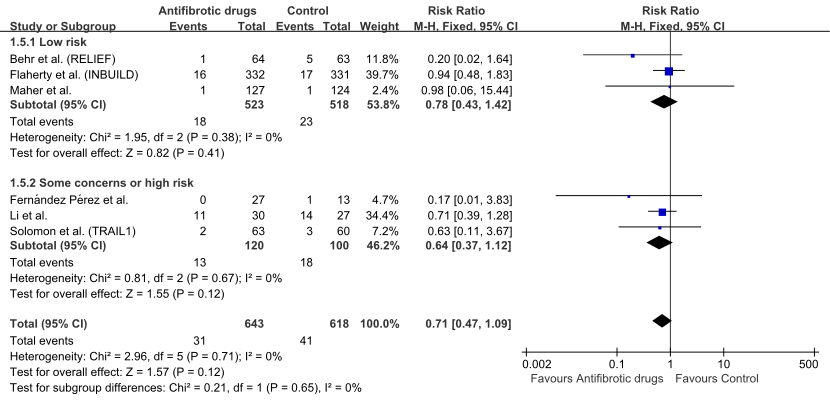
**

**2.2.2 All-cause mortality:pirfenidone vs nintedanib**

**
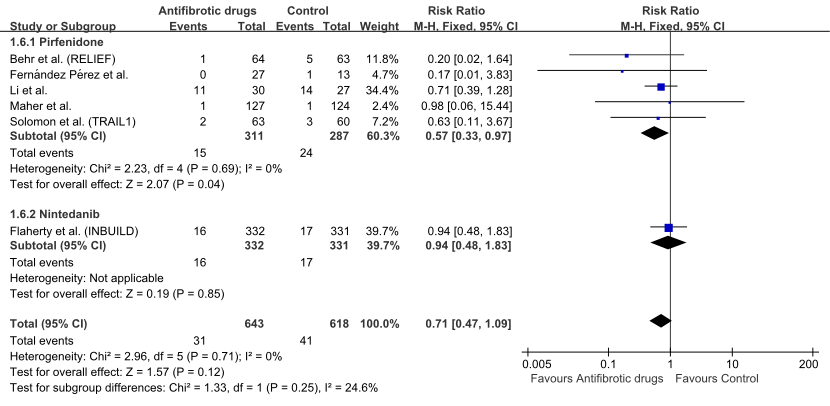
**

**2.2.3 All-cause mortality:＜12 months vs ≥ 12 months of follow up**

**
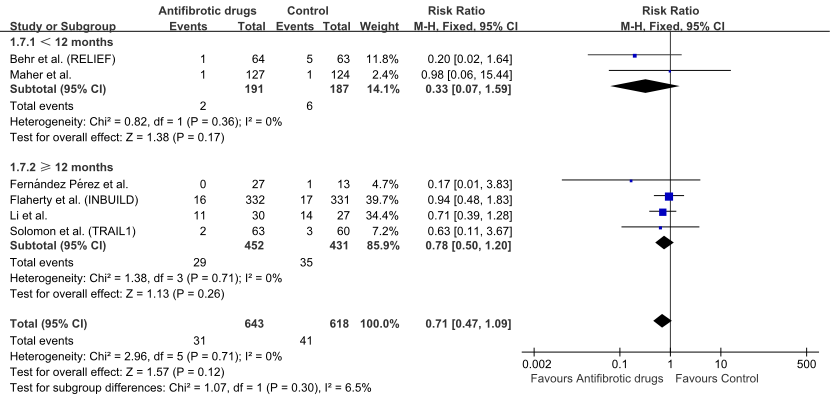
**

**2.2.4 All-cause mortality: taking vs not taking mycophenolate at baseline**

**
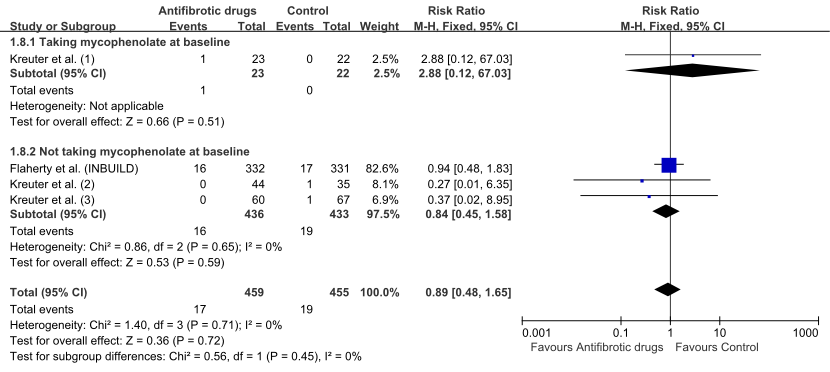
**

**2.3 SAEs**

**2.3.1 SAEs: low RoB vs some concerns of high RoB**

**
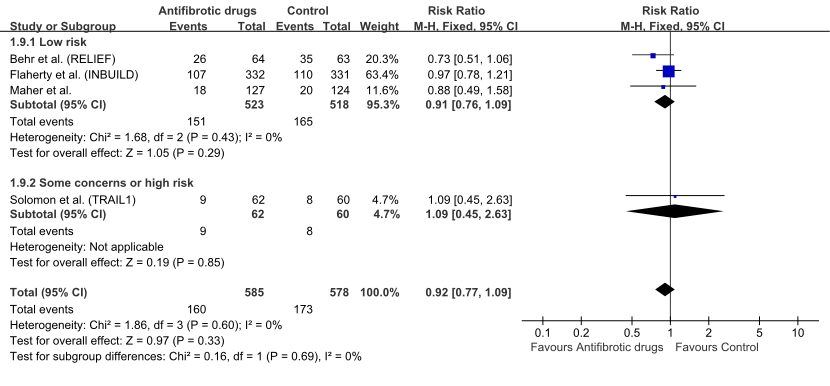
**

**2.3.2 SAEs: pirfenidone vs nintedanib**

**
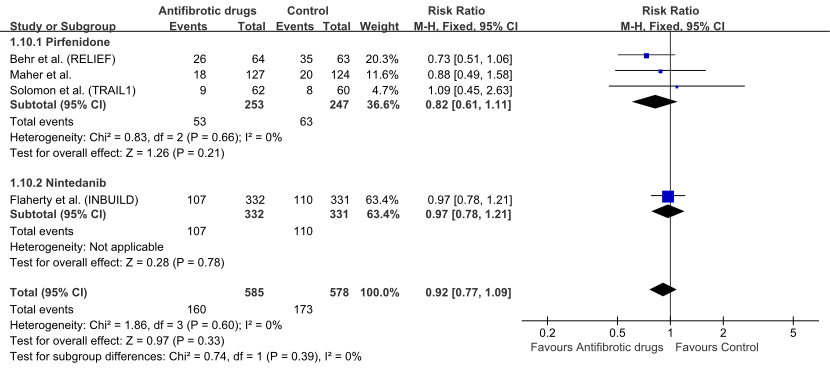
**

**2.3.3 SAEs: ＜12 months vs ≥ 12 months of follow up**

**
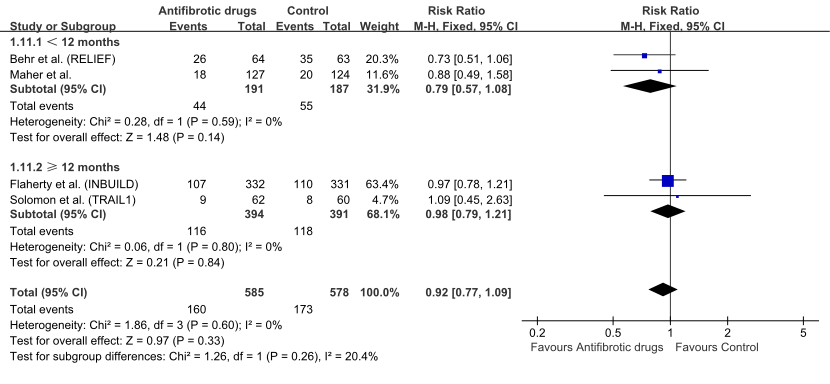
**

**2.3.4 SAEs: taking vs not taking mycophenolate at baseline**


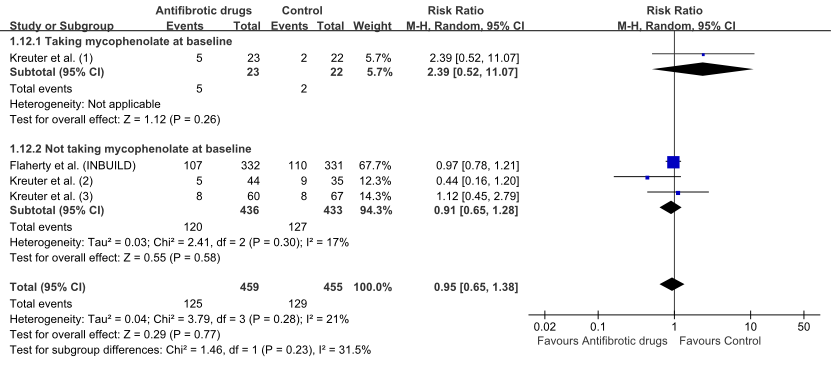


**2.4 Absolute change in FVC% predicted**

**2.4.1 Absolute change in FVC% predicted: low RoB vs some concerns or high RoB**

**
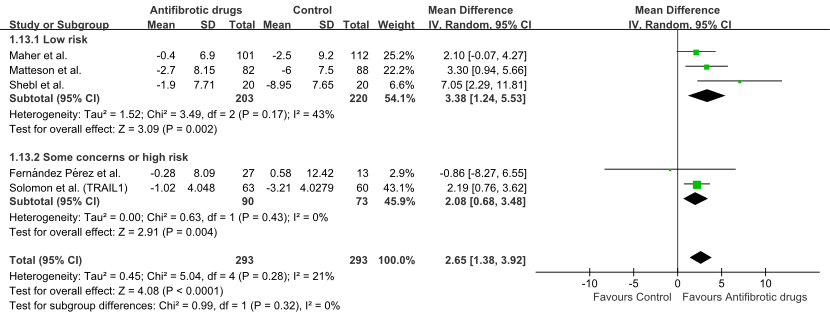
**

**2.4.2 Absolute change in FVC% predicted: pirfenidone vs nintedanib**

**
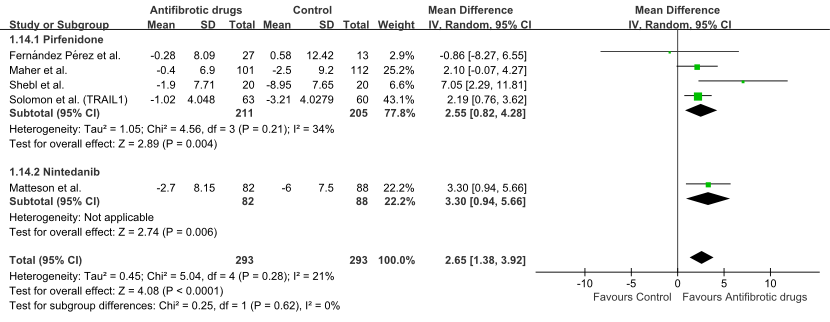
**

**2.4.3 Absolute change in FVC% predicted:＜12 months vs ≥ 12 months of follow up**


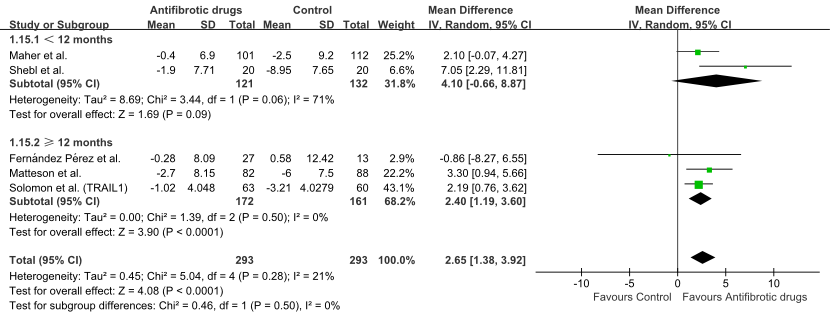


**Table 1. Risk-of-bias assessment**

**Table 1.1 NOS assessment of the observational studies**

|  | **Selection** | | | | Comparability | **Outcome** | | | Total  score |
| --- | --- | --- | --- | --- | --- | --- | --- | --- | --- |
| Study | Exposed  cohort | Nonexposed  Cohort | Ascertainment  of Exposure | Outcome  of Interest |  | Assessment  of outcome | Length of  Follow-up | Adequacy  of follow-up |  |
| Li et al^1^ | * | * | * | * | ** | * | * | * | 9 |
| Wang et al ^2^ | * | * | * | * | ... | * | * | * | 7 |

**Table 1.2 RoB2 assessment for absolute change in FVC**

| **Study** | **Randomisation**  **process** | **Deviations from intended interventions** | **Missing outcome data** | **Measurement of the outcome** | **Selection of the reported result** | **Overall risk of bias** |
| --- | --- | --- | --- | --- | --- | --- |
| Behr et al,  2021^10^ | Low | Low | Some concerns^a^ | Low | Low | **Some concerns** |
| Distler et al,  2019^4^ | Low | Low | Low | Low | Low | **Low** |
| Maher et al, 2020^8^ | Low | Low | Low | Low | Low | **Low** |
| Mateos-  Toledo et al, 2020^9^ | Low | Low | High^b^ | Low | Low | **High** |
| Matteson et al, 2022^21^ | Low | Low | Low | Low | Low | **Low** |
| Shebl et al, 2021^11^ | Low | Low | Low | Low | Low | **Low** |
| Solomon et al, 2023^14^ | Some concerns^c^ | Low | Low | Low | Low | **Some concerns** |

^a^The slow recruitment rate resulted in early termination of the trial. The result may be biased by the missing outcome data.

^b^13.6% of participants withdrew from the study, which may bias the results.

^c^There are differences in predominant HRCT patterns between groups at baseline.

Table 1.3 RoB2 assessment for all-cause mortality

| **Study** | **Randomisation**  **process** | **Deviations from intended interventions** | **Missing outcome data** | **Measurement of the outcome** | **Selection of the reported result** | **Overall risk of bias** |
| --- | --- | --- | --- | --- | --- | --- |
| Acharya et al, 2020^7^ | Low | Low | Low | Low | Low | **Low** |
| Behr et al, 2021^10^ | Low | Low | Low | Low | Low | **Low** |
| Distler et al, 2019^4^ | Low | Low | Low | Low | Low | **Low** |
| Flaherty et al, 2019^6^ | Low | Low | Low | Low | Low | **Low** |
| Maher et al, 2020^8^ | Low | Low | Low | Low | Low | **Low** |
| Mateos-Toledo et al, 2020^9^ | Low | Low | High^d^ | Low | Low | **High** |
| Fernández Pérez et al, 2023^12^ | Some concerns^e^ | Low | Low | Low | Low | **Some concerns** |
| Rimner et al, 2023^13^ | Some concerns^e^ | Low | High^f^ | Low | Low | **High** |
| Solomon et al, 2023^14^ | Some concerns^e^ | Low | Low | Low | Low | **Some concerns** |

^d^13.6% of participants withdrew from the study, which may bias the results.

^e^Inbalanced baseline characteristics.

^f^11.8% of participants withdrew from the study, which may bias the results.

**Table 1.4 RoB2 assessment for SAEs**

| **Study** | **Randomisation**  **process** | **Deviations from intended interventions** | **Missing outcome data** | **Measurement of the outcome** | **Selection of the reported result** | **Overall risk of bias** |
| --- | --- | --- | --- | --- | --- | --- |
| Acharya et al, 2020^7^ | Low | Low | Low | Low | Low | **Low** |
| Behr et al, 2021^10^ | Low | Low | Low | Low | Low | **Low** |
| Distler et al, 2019^4^ | Low | Low | Low | Low | Low | **Low** |
| Flaherty et al, 2019^6^ | Low | Low | Low | Low | Low | **Low** |
| Maher et al, 2020^8^ | Low | Low | Low | Low | Low | **Low** |
| Mateos-Toledo et al, 2020^9^ | Low | Low | High^h^ | Low | Low | **High** |
| Rimner et al, 2023^13^ | Some concerns^g^ | Low | High^h^ | Low | Low | **High** |
| Solomon et al, 2023^14^ | Some concerns^g^ | Low | Low | Low | Low | **Some concerns** |

^g^Inbalanced baseline characteristics.

^h^More than 10% of participants withdrew from the study, which may bias the results.

**Table 1.5 RoB2 assessment for absolute change in FVC% predicted**

| **Study** | **Randomisation**  **process** | **Deviations from intended interventions** | **Missing outcome data** | **Measurement of the outcome** | **Selection of the reported result** | **Overall risk of bias** |
| --- | --- | --- | --- | --- | --- | --- |
| Maher et al, 2020^8^ | Low | Low | Low | Low | Low | **Low** |
| Mateos-Toledo et al, 2020^9^ | Low | Low | High^i^ | Low | Low | **High** |
| Matteson et al, 2022^21^ | Low | Low | Low | Low | Low | **Low** |
| Fernández Pérez et al, 2023^12^ | Some concerns^j^ | Low | Low | Low | Low | **Some concerns** |
| Shebl et al, 2021^11^ | Low | Low | Low | Low | Low | **Low** |
| Solomon et al, 2023^14^ | Some concerns^j^ | Low | Low | Low | Low | **Some concerns** |

^i^13.6% of participants withdrew from the study, which may bias the results.

^j^Inbalanced baseline characteristics.

**Table 1.6 RoB2 assessment for absolute decline in FVC ≥10% predicted**

| **Study** | **Randomisation**  **process** | **Deviations from intended interventions** | **Missing outcome data** | **Measurement of the outcome** | **Selection of the reported result** | **Overall risk of bias** |
| --- | --- | --- | --- | --- | --- | --- |
| Acharya et al, 2020^7^ | Low | Low | Low | Low | Low | **Low** |
| Distler et al, 2019^4^ | Low | Low | Low | Low | Low | **Low** |
| Flaherty et al, 2022^22^ | Low | Low | Low | Low | Low | **Low** |
| Maher et al, 2020^8^ | Low | Low | Low | Low | Low | **Low** |
| Solomon et al, 2023^14^ | Some concerns^k^ | Low | Low | Low | Low | **Some concerns** |

^k^Inbalanced baseline characteristics.

**Table 1.7 RoB2 assessment for annual rate of decline in FVC**

| **Study** | **Randomisation**  **process** | **Deviations from intended interventions** | **Missing outcome data** | **Measurement of the outcome** | **Selection of the reported result** | **Overall risk of bias** |
| --- | --- | --- | --- | --- | --- | --- |
| Distler et al, 2019^4^ | Low | Low | Low | Low | Low | **Low** |
| Flaherty et al, 2019^6^ | Low | Low | Low | Low | Low | **Low** |
| Solomon et al, 2023^14^ | Some concerns^l^ | Low | Low | Low | Low | **Some concerns** |

^l^Inbalanced baseline characteristics.

**Table 1.8 RoB2 assessment for annual rate of decline in FVC% predicted**

| **Study** | **Randomisation**  **process** | **Deviations from intended interventions** | **Missing outcome data** | **Measurement of the outcome** | **Selection of the reported result** | **Overall risk of bias** |
| --- | --- | --- | --- | --- | --- | --- |
| Distler et al, 2019^4^ | Low | Low | Low | Low | Low | **Low** |
| Fernández Pérez et al, 2023^12^ | Some concerns^m^ | Low | Low | Low | Low | **Some concerns** |
| Solomon et al, 2023^14^ | Some concerns^m^ | Low | Low | Low | Low | **Some concerns** |

^m^Inbalanced baseline characteristics.

**Table 1.9 RoB2 assessment for absolute change in DLCO% predicted**

| **Study** | **Randomisation**  **process** | **Deviations from intended interventions** | **Missing outcome data** | **Measurement of the outcome** | **Selection of the reported result** | **Overall risk of bias** |
| --- | --- | --- | --- | --- | --- | --- |
| Distler et al, 2019^4^ | Low | Low | Low | Low | Low | **Low** |
| Maher et al, 2020^8^ | Low | Low | Low | Low | Low | **Low** |
| Mateos-Toledo et al, 2020^9^ | Low | Low | High^n^ | Low | Low | **High** |
| Fernández Pérez et al, 2023^12^ | Some concerns^o^ | Low | Low | Low | Low | **Some concerns** |

^n^13.6% of participants withdrew from the study, which may bias the results.

^o^Inbalanced baseline characteristics.

**Table 1.10 RoB2 assessment for absolute change in 6MWD**

| **Study** | **Randomisation**  **process** | **Deviations from intended interventions** | **Missing outcome data** | **Measurement of the outcome** | **Selection of the reported result** | **Overall risk of bias** |
| --- | --- | --- | --- | --- | --- | --- |
| Behr et al, 2021^10^ | Low | Low | Some concerns^p^ | Low | Low | **Some concerns** |
| Maher et al, 2020^8^ | Low | Low | Low | Low | Low | **Low** |
| Shebl et al, 2021^11^ | Low | Low | Low | Low | Low | **Low** |

^p^The slow recruitment rate resulted in early termination of the trial. The result may be biased by the missing outcome data.

**Table 1.11 RoB2 assessment for absolute change in SGRQ**

| **Study** | **Randomisation**  **process** | **Deviations from intended interventions** | **Missing outcome data** | **Measurement of the outcome** | **Selection of the reported result** | **Overall risk of bias** |
| --- | --- | --- | --- | --- | --- | --- |
| Distler et al, 2019^4^ | Low | Low | Low | Low | Low | **Low** |
| Shebl et al, 2021^11^ | Low | Low | Low | Low | Low | **Low** |
| Mateos-Toledo et al, 2020^9^ | Low | Low | High^q^ | Low | Low | **High** |
| Fernández Pérez et al, 2023^12^ | Some concerns^r^ | Low | Low | Low | Low | **Some concerns** |

^q^13.6% of participants withdrew from the study, which may bias the results.

^r^Inbalanced baseline characteristics.

**Table 1.12 RoB2 assessment for acute exacerbation of ILD**

| **Study** | **Randomisation**  **process** | **Deviations from intended interventions** | **Missing outcome data** | **Measurement of the outcome** | **Selection of the reported result** | **Overall risk of bias** |
| --- | --- | --- | --- | --- | --- | --- |
| Mateos-Toledo et al, 2020^9^ | Low | Low | High^s^ | Low | Low | **High** |
| Matteson et al, 2022^21^ | Low | Low | Low | Low | Low | **Low** |
| Fernández Pérez et al, 2023^12^ | Some concerns^t^ | Low | Low | Low | Low | **Some concerns** |
| Rimner et al, 2023^13^ | Some concerns^t^ | Low | High^u^ | Low | Low | **High** |
| Solomon et al, 2023^14^ | Some concerns^t^ | Low | Low | Low | Low | **Some concerns** |

^s^13.6% of participants withdrew from the study, which may bias the results.

^t^Inbalanced baseline characteristics.

^u^11.8% of participants withdrew from the study, which may bias the results.

**Table 1.13 RoB2 assessment for AE of diarrhea**

| **Study** | **Randomisation**  **process** | **Deviations from intended interventions** | **Missing outcome data** | **Measurement of the outcome** | **Selection of the reported result** | **Overall risk of bias** |
| --- | --- | --- | --- | --- | --- | --- |
| Acharya et al, 2020^7^ | Low | Low | Low | Low | Low | **Low** |
| Distler et al, 2019^4^ | Low | Low | Low | Low | Low | **Low** |
| Flaherty et al, 2019^6^ | Low | Low | Low | Low | Low | **Low** |
| Mateos-Toledo et al, 2020^9^ | Low | Low | High^v^ | Low | Low | **High** |
| Fernández Pérez et al, 2023^12^ | Some concerns^w^ | Low | Low | Low | Low | **Some concerns** |
| Rimner et al, 2023^13^ | Some concerns^w^ | Low | High^x^ | Low | Low | **High** |
| Solomon et al, 2023^14^ | Some concerns^w^ | Low | Low | Low | Low | **Some concerns** |

^v^13.6% of participants withdrew from the study, which may bias the results.

^w^Inbalanced baseline characteristics.

^x^11.8% of participants withdrew from the study, which may bias the results.

**Table 1.14 RoB2 assessment for AE of nausea**

| **Study** | **Randomisation**  **process** | **Deviations from intended interventions** | **Missing outcome data** | **Measurement of the outcome** | **Selection of the reported result** | **Overall risk of bias** |
| --- | --- | --- | --- | --- | --- | --- |
| Distler et al, 2019^4^ | Low | Low | Low | Low | Low | **Low** |
| Flaherty et al, 2019^6^ | Low | Low | Low | Low | Low | **Low** |
| Mateos-Toledo et al, 2020^9^ | Low | Low | High^y^ | Low | Low | **High** |
| Fernández Pérez et al, 2023^12^ | Some concerns^z^ | Low | Low | Low | Low | **Some concerns** |
| Rimner et al, 2023^13^ | Some concerns^z^ | Low | High^A^ | Low | Low | **High** |
| Solomon et al, 2023^14^ | Some concerns^z^ | Low | Low | Low | Low | **Some concerns** |

^y^13.6% of participants withdrew from the study, which may bias the results.

^z^Inbalanced baseline characteristics.

^A^11.8% of participants withdrew from the study, which may bias the results.

**Table 1.15 RoB2 assessment for AE of vomiting**

| **Study** | **Randomisation**  **process** | **Deviations from intended interventions** | **Missing outcome data** | **Measurement of the outcome** | **Selection of the reported result** | **Overall risk of bias** |
| --- | --- | --- | --- | --- | --- | --- |
| Distler et al, 2019^4^ | Low | Low | Low | Low | Low | **Low** |
| Flaherty et al, 2019^6^ | Low | Low | Low | Low | Low | **Low** |
| Mateos-Toledo et al, 2020^9^ | Low | Low | High^B^ | Low | Low | **High** |
| Fernández Pérez et al, 2023^12^ | Some concerns^C^ | Low | Low | Low | Low | **Some concerns** |
| Solomon et al, 2023^14^ | Some concerns^C^ | Low | Low | Low | Low | **Some concerns** |

^B^13.6% of participants withdrew from the study, which may bias the results.

^C^Inbalanced baseline characteristics.

**Table 1.16 RoB2 assessment for elevation of transaminases**

| **Study** | **Randomisation**  **process** | **Deviations from intended interventions** | **Missing outcome data** | **Measurement of the outcome** | **Selection of the reported result** | **Overall risk of bias** |
| --- | --- | --- | --- | --- | --- | --- |
| Acharya et al, 2020^7^ | Low | Low | Low | Low | Low | **Low** |
| Flaherty et al, 2019^6^ | Low | Low | Low | Low | Low | **Low** |
| Fernández Pérez et al, 2023^12^ | Some concerns^D^ | Low | Low | Low | Low | **Some concerns** |

^D^Inbalanced baseline characteristics.

**Table 1.17 RoB2 assessment for AEs leading to treatment discontinuation**

| **Study** | **Randomisation**  **process** | **Deviations from intended interventions** | **Missing outcome data** | **Measurement of the outcome** | **Selection of the reported result** | **Overall risk of bias** |
| --- | --- | --- | --- | --- | --- | --- |
| Distler et al, 2019^4^ | Low | Low | Low | Low | Low | **Low** |
| Flaherty et al, 2019^6^ | Low | Low | Low | Low | Low | **Low** |
| Maher et al, 2020^8^ | Low | Low | Low | Low | Low | **Low** |
| Mateos-Toledo et al, 2020^9^ | Low | Low | High^E^ | Low | Low | **High** |
| Fernández Pérez et al, 2023^12^ | Some concerns^F^ | Low | Low | Low | Low | **Some concerns** |
| Rimner et al, 2023^13^ | Some concerns^F^ | Low | High^G^ | Low | Low | **High** |
| Solomon et al, 2023^14^ | Some concerns^F^ | Low | Low | Low | Low | **Some concerns** |

^E^13.6% of participants withdrew from the study, which may bias the results.

^F^Inbalanced baseline characteristics.

^G^11.8% of participants withdrew from the study, which may bias the results.

**Table 1.18 RoB2 assessment for fatal AEs**

| **Study** | **Randomisation**  **process** | **Deviations from intended interventions** | **Missing outcome data** | **Measurement of the outcome** | **Selection of the reported result** | **Overall risk of bias** |
| --- | --- | --- | --- | --- | --- | --- |
| Acharya et al, 2020^7^ | Low | Low | Low | Low | Low | **Low** |
| Behr et al, 2021^10^ | Low | Low | Low | Low | Low | **Low** |
| Distler et al, 2019^4^ | Low | Low | Low | Low | Low | **Low** |
| Flaherty et al, 2019^6^ | Low | Low | Low | Low | Low | **Low** |
| Maher et al, 2020^8^ | Low | Low | Low | Low | Low | **Low** |
| Mateos-Toledo et al, 2020^9^ | Low | Low | High^H^ | Low | Low | **High** |
| Fernández Pérez et al, 2023^12^ | Some concerns^I^ | Low | Low | Low | Low | **Some concerns** |
| Rimner et al, 2023^13^ | Some concerns^I^ | Low | High^J^ | Low | Low | **High** |
| Solomon et al, 2023^14^ | Some concerns^I^ | Low | Low | Low | Low | **Some concerns** |

^H^13.6% of participants withdrew from the study, which may bias the results.

^I^Inbalanced baseline characteristics.

^J^11.8% of participants withdrew from the study, which may bias the results.

**Table 1.19 RoB2 assessment for respiratory-related mortality**

| **Study** | **Randomisation**  **process** | **Deviations from intended interventions** | **Missing outcome data** | **Measurement of the outcome** | **Selection of the reported result** | **Overall risk of bias** |
| --- | --- | --- | --- | --- | --- | --- |
| Acharya et al, 2020^7^ | Low | Low | Low | Low | Low | **Low** |
| Behr et al, 2021^10^ | Low | Low | Low | Low | Low | **Low** |
| Distler et al, 2019^4^ | Low | Low | Low | Low | Low | **Low** |
| Mateos-Toledo et al, 2020^9^ | Low | Low | High^K^ | Low | Low | **High** |
| Fernández Pérez et al, 2023^12^ | Some concerns^L^ | Low | Low | Low | Low | **Some concerns** |
| Rimner et al, 2023^13^ | Some concerns^L^ | Low | High^M^ | Low | Low | **High** |
| Solomon et al, 2023^14^ | Some concerns^L^ | Low | Low | Low | Low | **Some concerns** |

^K^13.6% of participants withdrew from the study, which may bias the results.

^L^Inbalanced baseline characteristics.

^M^11.8% of participants withdrew from the study, which may bias the results.

**Table 2. Overview of subgroup analyses in all participants**

| **Outcome measure** | **Subgroups** | **No.**  **Patients** | **MD / RR (95% CI) for antifibrotic drugs vs control** | **Analysis model, *I^2^*** | ***P* value for overall effect** | ***P* value for subgroup difference** |
| --- | --- | --- | --- | --- | --- | --- |
| **Primary outcomes** | | | | | | |
| Absolute change of FVC (ml) | | | | | | |
| 4 studies | low RoB | 999 | 86.21 (49.38, 123.03) | RE, 64% | ＜0.01 | FE, 0.40  RE, 0.79 |
| 3 studies | Some concerns or high risk | 209 | 77.68 (26.60, 128.76) | FE, 0% | ＜0.01 |
| 5 studies | Pirfenidone | 462 | 99.98 (97.94, 102.02) | FE, 0% | ＜0.01 | FE, 0.01  RE, 0.03 |
| 2 studies | Nintedanib | 746 | 56.67 (18.81, 94.54) | RE, 9% | ＜0.01 |
| 3 studies | ＜12 months | 320 | 100.01 (97.96, 102.05) | FE, 0% | ＜0.01 | FE, 0.01  RE, 0.01 |
| 4 studies | ≥ 12 months | 888 | 61.38 (32.11, 90.64) | FE, 0% | ＜0.01 |
| 2 studies | AID-ILD | 746 | 56.67 (18.81, 94.54) | RE, 9% | ＜0.01 | RE, 0.07  FE, 0.04 |
| 2 studies | Hypersensitivity pneumonitis | 62 | 123.67 (17.20, 230.13) | FE, 0% | 0.02 |
| 1 study | Unclassifiable ILD | 213 | 100.00 (97.96, 102.04) | NA | ＜0.01 |
| 2 studies | Taking mycophenolate at baseline | 324 | 17.08 (-56.22, 90.37) | RE, 24% | 0.65 | FE, 0.08  RE, 0.12 |
| 4 studies | Not taking mycophenolate at baseline | 694 | 81.47 (44.75, 118.19) | FE, 0% | ＜0.01 |
| All-cause mortality | | | | | |  |
| 5 studies | low RoB | 1650 | 0.87 (0.53, 1.43) | RE, 0% | 0.71 | FE, 0.72  RE, 0.53 |
| 5 studies | Some concerns or high risk | 270 | 0.76 (0.44, 1.31) | FE, 0% | 0.33 |
| 7 studies | Pirfenidone | 651 | 0.57 (0.33, 0.97) | FE, 0% | 0.04 | FE, 0.09  RE, 0.18 |
| 3 studies | Nintedanib | 1269 | 1.08 (0.65, 1.82) | FE, 0% | 0.76 |
| 3 studies | ＜12 months | 411 | 0.33 (0.07, 1.59) | FE, 0% | 0.17 | FE, 0.23  RE, 0.32 |
| 7 studies | ≥ 12 months | 1509 | 0.89 (0.61, 1.31) | FE, 0% | 0.57 |
| 5 studies | AID-ILD | 959 | 0.82 (0.53, 1.25) | FE, 0% | 0.36 | FE, 0.48  RE, 0.48 |
| 2 studies | Hypersensitivity pneumonitis | 60 | 0.17 (0.01, 3.83) | FE, 0% | 0.26 |
| 1 study | Radiation pneumonitis | 30 | 4.79 (0.27, 85.15) | FE, NA | 0.29 |
| 1 study | Unclassifiable ILD | 251 | 0.98 (0.06, 15.44) | FE, NA | 0.99 |
| 2 studies | Taking mycophenolate at baseline | 324 | 2.19 (0.50, 9.62) | FE, 0% | 0.30 | FE, 0.24  RE, 0.25 |
| 4 studies | Not taking mycophenolate at baseline | 1166 | 0.84 (0.49, 1.45) | FE, 0% | 0.54 |
| 2 studies^a^ | UIP | 423 | 0.69 (0.33, 1.45) | FE, NA | 0.32 | FE, 0.09  RE, 0.09 |
| 2 studies^a^ | Non-UIP | 274 | 4.96 (0.59, 41.85) | FE, NA | 0.14 |
| Serious AEs | | | | | |  |
| 5 studies | low RoB | 1650 | 0.95 (0.82, 1.11) | RE, 0% | 0.54 | FE, 0.54  RE, 0.78 |
| 4 studies | Some concerns or high risk | 308 | 1.11 (0.39, 3.17) | RE, 39% | 0.84 |
| 6 studies^b^ | Pirfenidone | 689 | 0.80 (0.60, 1.06) | RE, 0% | 0.12 | FE, 0.16  RE, 0.16 |
| 3 studies^b^ | Nintedanib | 1269 | 1.05 (0.81, 1.34) | RE, 31% | 0.73 |
| 4 studies | ＜12 months | 547 | 0.78 (0.57, 1.07) | FE, 0% | 0.12 | FE, 0.11  RE, 0.10 |
| 5 studies | ≥ 12 months | 1411 | 1.05 (0.88, 1.25) | FE, 0% | 0.58 |
| 5 studies | AID-ILDs | 1037 | 1.08 (0.86, 1.37) | RE, 0% | 0.50 | FE, 0.80  RE, 0.80 |
| 2 studies^b^ | Hypersensitivity pneumonitis | 193 | 0.90 (0.61, 1.34) | RE, NA | 0.62 |
| 2 studies | Unclassifiable ILDs | 365 | 1.03 (0.71, 1.50) | RE, 0% | 0.88 |
| 1 study | iNSIP | 125 | 0.78 (0.42, 1.45) | RE, NA | 0.44 |
| 2 studies | Other ILDs | 111 | 1.97 (0.19, 19.88) | RE, 65% | 0.57 |
| 2 studies | Taking mycophenolate at baseline | 324 | 1.71 (1.09, 2.70) | FE, 0% | 0.02 | FE, 0.01  RE, 0.01 |
| 4 studies | Not taking mycophenolate at baseline | 1186 | 0.92 (0.76, 1.10) | RE, 0% | 0.35 |
| Fatal AEs | | | | | |  |
| 5 studies | low RoB | 1650 | 0.66 (0.37, 1.19) | FE, 0% | 0.17 | FE, 0.41  RE, 0.62 |
| 5 studies | Some concerns or high risk | 348 | 1.00 (0.27, 3.69) | RE, 7% | 0.99 |
| 7 studies | Pirfenidone | 729 | 0.51 (0.19, 1.32) | FE, 0% | 0.17 | FE, 0.35  RE, 0.43 |
| 3 studies | Nintedanib | 1269 | 0.88 (0.42, 1.82) | RE, 12% | 0.72 |
| 4 studies | ＜12 months | 547 | 0.62 (0.12, 3.11) | RE, 14% | 0.56 | FE, 0.60  RE, 0.81 |
| 6 studies | ≥ 12 months | 1451 | 0.79 (0.45, 1.39) | FE, 0% | 0.41 |
| 2 studies | Taking mycophenolate at baseline | 324 | 1.79 (0.39, 8.28) | FE, 0% | 0.46 | FE, 0.22  RE, 0.24 |
| 3 studies | Not taking mycophenolate at baseline | 1166 | 0.63 (0.33, 1.22) | FE, 0% | 0.17 |
| **Secondary outcomes** | | | | | | |
| Absolute change in FVC% predicted | | | | | |  |
| 3 studies | low RoB | 423 | 3.38 (1.24, 5.53) | RE, 43% | 0.002 | FE, 0.34  RE, 0.32 |
| 3 studies | Some concerns or high risk | 185 | 2.10 (0.71, 3.48) | RE, 0% | 0.003 |
| 5 studies | Pirfenidone | 438 | 2.45 (1.08, 3.83) | RE, 12% | ＜0.001 | FE, 0.49  RE, 0.54 |
| 1 study | Nintedanib | 170 | 3.30 (0.94, 5.66) | RE, NA | 0.006 |
| 2 studies | ＜12 months | 253 | 4.10 (-0.66, 8.87) | RE, 71% | 0.090 | FE, 0.64  RE, 0.50 |
| 4 studies | ≥ 12 months | 355 | 2.40 (1.21, 3.60) | FE, 0% | ＜0.001 |
| 2 studies | AID-ILDs | 293 | 2.49 (1.27, 3.71) | RE, 0% | ＜0.001 | FE, 0.57  RE, 0.84 |
| 3 studies | Hypersensitivity pneumonitis | 102 | 3.70 (-1.22, 8.61) | RE, 39% | 0.140 |
| 1 study | Unclassifiable ILDs | 213 | 2.10 (-0.07, 4.27) | RE, NA | 0.060 |
| Absolute decline in FVC ≥ 10% predicted | | | | | |  |
| 4 studies | low RoB | 1525 | 0.69 (0.58, 0.81) | FE, 0% | ＜0.001 | FE, 0.99  RE, 0.97 |
| 1 study | Some concerns or high risk | 123 | 0.68 (0.23, 2.03) | FE, NA | 0.490 |
| 3 studies | Pirfenidone | 410 | 0.53 (0.33, 0.83) | FE, 0% | 0.005 | FE, 0.19  RE, 0.22 |
| 2 studies | Nintedanib | 1238 | 0.73 (0.61, 0.87) | FE, 0% | ＜0.001 |
| 2 study | ＜12 months | 287 | 0.50 (0.30, 0.82) | FE, 0% | 0.006 | FE, 0.16  RE, 0.18 |
| 3 studies | ≥ 12 months | 1361 | 0.72 (0.61, 0.86) | FE, 0% | ＜0.001 |
| 2 studies | Taking mycophenolate at baseline | 323 | 0.74 (0.33, 1.66) | FE, 0% | 0.47 | FE, 0.88  RE, 0.83 |
| 3 studies | Not taking mycophenolate at baseline | 1168 | 0.70 (0.59, 0.83) | FE, 2% | ＜0.01 |
| 2 studies | UIP | 423 | 0.76 (0.61, 0.95) | FE, 8% | 0.02 | FE, 0.22  RE, 0.46 |
| 2 studies | Non-UIP | 274 | 0.60 (0.43, 0.82) | FE, 0% | ＜0.01 |
| Annual rate of decline in FVC (ml/yr) | | | | | |  |
| 2 studies | low RoB | 1239 | 73.39 (8.62, 138.15) | RE, 81% | 0.03 | FE, 0.78  RE, 0.88 |
| 1 study | Some concerns or high risk | 123 | 80.00 (21.79, 138.21) | RE, NA | ＜0.01 |
| 1 study | Pirfenidone | 123 | 80.00 (21.79, 138.21) | RE, NA | ＜0.01 | FE, 0.78  RE, 0.88 |
| 2 studies | Nintedanib | 1239 | 73.39 (8.62, 138.15) | RE, 81% | 0.03 |
| 3 studies | ≥ 12 months (all studies) | 1362 | 74.88 (32.15, 117.61) | RE, 63% | ＜0.01 | NA |
| 3 studies | AID-ILDs | 869 | 59.09 (29.46, 88.72) | FE, 20% | ＜0.01 | FE, 0.14  RE, 0.18 |
| 1 study | Hypersensitivity pneumonitis | 173 | 73.10 (-8.60, 154.80) | FE, NA | 0.08 |
| 1 study | Unclassifiable ILDs | 114 | 68.30 (-31.40, 168.00) | FE, NA | 0.18 |
| 1 study | iNSIP | 125 | 141.60 (46.00, 237.20) | FE, NA | ＜0.01 |
| 1 study | Other ILDs | 81 | 197.10 (77.60, 316.60) | FE, NA | ＜0.01 |
| 1 study | Taking mycophenolate at baseline | 279 | 26.30 (-27.89, 80.49) | FE, NA | 0.34 | FE, 0.06  RE, 0.13 |
| 2 studies | Not taking mycophenolate at baseline | 960 | 83.98 (33.70, 134.25) | RE, 56% | ＜0.01 |
| 2 studies | UIP | 493 | 127.41 (81.51, 173.32) | FE, 0% | ＜0.01 | FE,＜0.01  RE, 0.05 |
| 2 studies | Non-UIP | 293 | 24.50 (-65.41, 114.40) | RE, 88% | 0.59 |
| Annual rate of decline in FVC% predicted | | | | | |  |
| 1 study | low RoB | 576 | 1.20 (0.09, 2.31) | FE, NA | 0.03 | FE, 0.28  RE, 0.28 |
| 2 studies | Some concerns or high risk | 163 | 2.18 (0.80, 3.55) | FE, 0% | ＜0.01 |
| 2 studies | Pirfenidone | 163 | 2.18 (0.80, 3.55) | FE, 0% | ＜0.01 | FE, 0.28  RE, 0.28 |
| 1 study | Nintedanib | 576 | 1.20 (0.09, 2.31) | FE, NA | 0.03 |
| 3 studies | ≥ 12 months | 739 | 1.59 (0.72, 2.45) | FE, 0% | ＜0.01 | NA |
| Absolute change in DLCO% predicted | | | | | |  |
| 2 studies | low RoB | 783 | 0.54 (-1.64, 2.71) | RE, 64% | 0.63 | FE, 0.05  RE, 0.14 |
| 2 studies | Some concerns or high risk | 62 | 7.64 (-1.46, 16.74) | RE, 36% | 0.10 |
| 3 studies | Pirfenidone | 269 | 4.21 (-1.46, 9.89) | RE, 47% | 0.15 | FE, 0.04  RE, 0.12 |
| 1 study | Nintedanib | 576 | -0.44 (-1.94, 1.06) | FE, NA | 0.56 |
| 1 study | ＜12 months | 207 | 1.80 (-0.37, 3.97) | FE, NA | 0.10 | FE, 0.15  RE, 0.63 |
| 3 studies | ≥ 12 months | 638 | 3.70 (-3.72, 11.11) | RE, 67% | 0.33 |
| Absolute change in 6MWD (m) | | | | | |  |
| 2 studies | low RoB | 247 | 28.69 (10.63, 46.75) | FE, 0% | ＜0.01 | FE, 0.91  RE, 0.91 |
| 1 study | Some concerns or high risk | 63 | 31.40 (-9.85, 72.65) | FE, NA | 0.14 |
| 3 studies | Pirfenidone (all studies) | 310 | 29.12 (12.58, 45.67) | FE, 0% | ＜0.01 | NA |
| 3 studies | ＜12 months (all studies) | 310 | 29.12 (12.58, 45.67) | FE, 0% | ＜0.01 | NA |
| Absolute change in total score of SGRQ | | | | | |  |
| 2 studies | low RoB | 616 | -1.99 (-9.14, 5.15) | RE, 95% | 0.58 | FE, 0.37  RE, 0.55 |
| 2 studies | Some concerns or high risk | 59 | -4.37 (-7.45, -1.29) | FE, 0% | ＜0.01 |
| 3 studies | Pirfenidone | 99 | -5.26 (-6.88, -3.63) | FE, 0% | ＜0.01 | FE,＜0.01  RE,＜0.01 |
| 1 study | Nintedanib | 576 | 1.69 (-0.73, 4.11) | FE, NA | 0.17 |
| 1 study | ＜12 months | 40 | -5.60 (-7.51, -3.69) | FE, NA | ＜0.01 | FE,＜0.01  RE, 0.22 |
| 3 studies | ≥ 12 months | 635 | -2.26 (-7.18, 2.66) | RE, 79% | 0.37 |
| Respiratory exacerbation within 12 months | | | | | |  |
| 1 study | low RoB | 170 | 0.54 (0.17, 1.71) | FE, NA | 0.29 | FE, 0.46  RE, 0.66 |
| 4 studies | Some concerns or high risk | 213 | 0.32 (0.15, 0.68) | FE, 0% | ＜0.01 |
| 3 studies | Pirfenidone | 183 | 0.22 (0.04, 1.04) | RE, 0% | 0.06 | FE, 0.21  RE, 0.34 |
| 2 studies | Nintedanib | 200 | 0.51 (0.24, 1.05) | FE, 0% | 0.07 |
| 5 studies | ≥ 12 months (all studies) | 383 | 0.38 (0.20, 0.73) | FE, 0% | ＜0.01 | NA |
| AE: Diarrhea | | | | | |  |
| 3 studies | low RoB | 1272 | 2.58 (2.25, 2.95) | FE, 0% | ＜0.01 | FE,＜0.01  RE, 0.02 |
| 5 studies | Some concerns or high risk | 348 | 1.16 (0.62, 2.16) | RE, 13% | 0.65 |
| 5 studies | Pirfenidone | 351 | 1.14 (0.71, 1.83) | FE, 0% | 0.58 | FE,＜0.01  RE,＜0.01 |
| 3 studies | Nintedanib | 1269 | 2.59 (2.26, 2.97) | FE, 0% | ＜0.01 |
| 2 studies | ＜12 months | 169 | 2.55 (0.39, 16.59) | FE, 0% | 0.33 | FE, 0.95  RE, 0.91 |
| 6 studies | ≥ 12 months | 1451 | 1.98 (1.38, 2.82) | RE, 68% | ＜0.01 |
| AE: Nausea | | | | | |  |
| 2 studies | low RoB (all for nintedanib) | 1239 | 2.65 (2.02, 3.49) | RE, 16% | ＜0.01 | FE, 0.36  RE, 0.36 |
| 4 studies | Some concerns or high risk | 212 | 1.66 (0.63, 4.36) | RE, 56% | 0.30 |
| 3 studies | Pirfenidone | 182 | 1.97 (0.76, 5.09) | RE, 61% | 0.16 | FE, 0.62  RE, 0.61 |
| 3 studies | Nintedanib | 1269 | 2.57 (1.73, 3.81) | RE, 43% | ＜0.01 |
| 6 studies | ≥ 12 months (all studies) | 1451 | 2.33 (1.61, 3.36) | RE, 45% | ＜0.01 | NA |
| AE: Vomiting | | | | | |  |
| 2 studies | low RoB (all for nintedanib) | 1239 | 2.81 (1.88, 4.20) | RE, 36% | ＜0.01 | FE, 0.94  RE, 0.95 |
| 3 studies | Some concerns or high risk | 182 | 2.91 (1.18, 7.17) | FE, 0% | 0.02 |
| 3 studies | Pirfenidone | 182 | 2.91 (1.18, 7.17) | FE, 0% | 0.02 | FE, 0.94  RE, 0.95 |
| 2 studies | Nintedanib | 1239 | 2.81 (1.88, 4.20) | RE, 36% | ＜0.01 |
| 5 studies | ≥ 12 months (all studies) | 1421 | 2.82 (2.10, 3.79) | FE, 0% | ＜0.01 | NA |
| AE: Elevation of transaminases | | | | | |  |
| 2 studies | low RoB | 696 | 1.90 (0.44, 8.18) | RE, 75% | 0.39 | FE, 0.03  RE, 0.17 |
| 2 studies | Some concerns or high risk | 176 | 0.36 (0.06, 2.20) | FE, 0% | 0.27 |
| 3 studies | Pirfenidone | 209 | 0.58 (0.20, 1.67) | FE, 0% | 0.31 | FE,＜0.01  RE,＜0.01 |
| 1 study | Nintedanib | 663 | 3.57 (1.92, 6.65) | FE, NA | ＜0.01 |
| 2 studies | ＜12 months | 169 | 0.72 (0.22, 2.31) | FE, 0% | 0.58 | FE, 0.03  RE, 0.78 |
| 2 studies | ≥ 12 months | 703 | 1.15 (0.06, 20.99) | RE, 72% | 0.92 |
| AEs leading to treatment discontinuation | | | | | |  |
| 3 studies | low RoB | 1490 | 2.02 (1.53, 2.68) | FE, 0% | ＜0.01 | FE, 0.44  RE, 0.51 |
| 4 studies | Some concerns or high risk | 212 | 2.87 (1.24, 6.64) | FE, 0% | 0.01 |
| 4 studies | Pirfenidone | 433 | 3.00 (1.58, 5.73) | FE, 0% | ＜0.01 | FE, 0.22  RE, 0.23 |
| 3 studies | Nintedanib | 1269 | 1.93 (1.44, 2.59) | FE, 0% | ＜0.01 |
| 1 study | ＜12 months | 251 | 3.71 (1.43, 9.63) | FE, 0% | ＜0.01 | FE, 0.22  RE, 0.21 |
| 6 studies | ≥ 12 months | 1451 | 1.98 (1.50, 2.61) | FE, 0% | ＜0.01 |
| 2 studies | Taking mycophenolate at baseline | 324 | 1.90 (0.91, 3.96) | FE, 0% | 0.09 | FE, 0.87  RE, 0.85 |
| 4 studies | Not taking mycophenolate at baseline | 1186 | 2.03 (1.50, 2.75) | FE, 0% | ＜0.01 |
| **Exploratory outcome** | | | | | | |
| Respiratory-related mortality | | | | | | |
| 3 studies | low RoB | 736 | 0.58 (0.10, 3.38) | RE, 32% | 0.54 | FE, 0.29  RE, 0.41 |
| 5 studies | Some concerns or high risk | 349 | 1.51 (0.42, 5.50) | FE, 0% | 0.53 |
| 6 studies | Pirfenidone | 479 | 0.65 (0.12, 3.58) | RE, 17% | 0.62 | FE, 0.37  RE, 0.55 |
| 2 studies | Nintedanib | 606 | 1.31 (0.39, 4.42) | FE, 0% | 0.66 |
| 3 studies | ＜12 months | 296 | 0.65 (0.03, 14.76) | RE, 52% | 0.79 | FE, 0.45  RE, 0.76 |
| 5 studies | ≥ 12 months | 789 | 1.10 (0.41, 2.98) | FE, 0% | 0.85 |

Results with statistical significance are marked in red.

AE = adverse event; AID = autoimmune disease; DLCO = diffusing capacity of the lung for carbon monoxide; FE = fixed effect; FVC = forced vital capacity; ILD = interstitial lung disease; iNSIP = idiopathic non-specific interstitial pneumonia; IPF = idiopathic pulmonary fibrosis; MD = mean difference; NA = not available; RE = random effects; RR = risk ratio; SGRQ = St. George’s Respiratory Questionnaire; 6MWD = six-minute walk distance; UIP = usual interstitial pneumonia.

**Table 3. Overview of subgroup analyses in patients with a progressive fibrosing phenotype**

| **Outcomes / subgroups** | **No. Studies** | **No.**  **Patients** | **MD / RR (95% CI) for antifibrotic drugs vs control** | **Analysis model, *I^2^*** | ***P* value for subgroup difference** |
| --- | --- | --- | --- | --- | --- |
| Absolute change of FVC (ml) | | | | | |
| All studies | 5 studies | 616 | 99.98 (97.94, 102.02) | FE, 0% | NA |
| low RoB | 3 studies | 423 | 100.01 (97.97, 102.05) | FE, 0% | FE, 0.45 RE, 0.45 |
| Some concerns or high risk | 2 studies | 193 | 79.59 (27.09, 132.09) | FE, 0% |
| Pirfenidone | 4 studies | 446 | 99.98 (97.94, 102.03) | FE, 0% | FE, 0.86 RE, 0.86 |
| Nintedanib | 1 study | 170 | 93.00 (14.80, 171.20) | NA |
| ＜12 months | 3 studies | 320 | 100.01 (97.96, 102.05) | FE, 0% | FE, 0.52 RE, 0.52 |
| ≥ 12 months | 2 studies | 296 | 84.63 (37.94, 131.33) | FE, 0% |
| Taking mycophenolate at baseline | 1 study | 45 | -55.30 (-202.51, 91.91) | NA | FE, 0.04 RE, 0.04 |
| Not taking mycophenolate at baseline | 2 studies | 378 | 107.46 (55.45, 159.46) | FE, 0% |
| All-cause mortality | | | | | |
| All studies | 6 studies | 1261 | 0.71 (0.47, 1.09) | FE, 0% | NA |
| low RoB | 3 studies | 1041 | 0.78 (0.43, 1.42) | FE, 0% | FE, 0.65 RE, 0.62 |
| Some concerns or high risk | 3 studies | 220 | 0.64 (0.37, 1.12) | FE, 0% |
| Pirfenidone | 5 studies | 598 | 0.57 (0.33, 0.97) | FE, 0% | FE, 0.25 RE, 0.35 |
| Nintedanib | 1 study | 663 | 0.94 (0.48, 1.83) | NA |
| ＜12 months | 2 studies | 378 | 0.33 (0.07, 1.59) | FE, 0% | FE, 0.30 RE, 0.39 |
| ≥ 12 months | 4 studies | 883 | 0.78 (0.50, 1.20) | FE, 0% |
| Taking mycophenolate at baseline | 1 studies | 45 | 2.88 (0.12, 67.03) | NA | FE, 0.45 RE, 0.46 |
| Not taking mycophenolate at baseline | 2 studies | 869 | 0.84 (0.45, 1.58) | FE, 0% |
| Serious adverse events | | | | | |
| All studies | 4 studies | 1163 | 0.92 (0.77, 1.09) | FE, 0% | NA |
| low RoB | 3 studies | 1041 | 0.91 (0.76, 1.09) | FE, 0% | FE, 0.69 RE, 0.68 |
| Some concerns or high risk | 1 study | 122 | 1.09 (0.45, 2.63) | NA |
| Pirfenidone | 3 studies | 500 | 0.82 (0.61, 1.11) | FE, 0% | FE, 0.39 RE, 0.31 |
| Nintedanib | 1 study | 663 | 0.97 (0.78, 1.21) | NA |
| ＜12 months | 2 studies | 378 | 0.79 (0.57, 1.08) | FE, 0% | FE, 0.26 RE, 0.22 |
| ≥ 12 months | 2 studies | 785 | 0.98 (0.79, 1.21) | FE, 0% |
| Taking mycophenolate at baseline | 1 study | 45 | 2.39 (0.52, 11.07) | NA | FE, 0.23 RE, 0.23 |
| Not taking mycophenolate at baseline | 2 studies | 869 | 0.91 (0.65, 1.28) | RE, 17% |
| Absolute change in FVC% predicted | | | | | |
| All studies | 5 studies | 586 | 2.65 (1.38, 3.92) | RE, 21% | NA |
| low RoB | 3 studies | 423 | 3.38 (1.24, 5.53) | RE, 43% | FE, 0.34 RE, 0.32 |
| Some concerns or high risk | 2 studies | 163 | 2.08 (0.68, 3.48) | FE, 0% |
| Pirfenidone | 4 studies | 416 | 2.55 (0.82, 4.28) | RE, 34% | FE, 0.49 RE, 0.62 |
| Nintedanib | 1 study | 170 | 3.30 (0.94, 5.66) | NA |
| ＜12 months | 2 studies | 253 | 4.10 (-0.66, 8.87) | RE, 71% | FE, 0.64 RE, 0.50 |
| ≥ 12 months | 3 studies | 333 | 2.40 (1.19, 3.60) | FE, 0% |

Results with statistical significance are marked in red.

FE = fixed effect; FVC = forced vital capacity; IPF = idiopathic pulmonary fibrosis; MD = mean difference; NA = not available; RE = random effects; RR = risk ratio.

Table 4. Sensitivity analyses for all outcomes

| **Outcome measure** | **No. Studies** | **Random effects model,**  **MD/RR, 95% CI** | **Fixed effect model,**  **MD/RR, 95% CI** | **Heterogeneity, *I^2^*** |
| --- | --- | --- | --- | --- |
| **Primary outcomes** | | | | |
| ***Absolute change in FVC (ml)*** | | | | |
| low RoB | 4 | 86.21 (49.38, 123.03) P<0.01 | 99.86 (97.82, 101.90) P<0.01 | 64% |
| All studies | 7 | 85.40 (61.11, 109.68) P<0.01 | 99.82 (97.78, 101.86) P<0.01 | 34% |
| ***All-cause mortality*** | | | | |
| low RoB | 5 | 0.91 (0.55, 1.51) P=0.71 | 0.87 (0.53, 1.43) P=0.59 | 0% |
| All studies | 10 | 0.81 (0.56, 1.18) P=0.28 | 0.83 (0.57, 1.20) P=0.32 | 0% |
| ***Serious adverse events*** | | | | |
| low RoB | 5 | 0.95 (0.82, 1.11) P=0.54 | 0.97 (0.83, 1.13) P=0.68 | 0% |
| All studies | 9 | 0.96 (0.82, 1.11) P=0.57 | 0.98 (0.84, 1.14) P=0.82 | 0% |
| ***Fatal adverse events*** | | | | |
| low RoB | 5 | 0.69 (0.38, 1.26) P=0.22 | 0.66 (0.37, 1.19) P=0.17 | 0% |
| All studies | 10 | 0.74 (0.43, 1.27) P=0.27 | 0.74 (0.45, 1.24) P=0.26 | 0% |
| **Secondary outcomes** | | | | |
| ***Absolute change in FVC% predicted*** | | | | |
| low RoB | 3 | 3.38 (1.24, 5.53) P<0.01 | 3.10 (1.58, 4.61) P<0.01 | 43% |
| All studies | 6 | 2.56 (1.52, 3.59) P<0.01 | 2.55 (1.53, 3.57) P<0.01 | 1% |
| ***Absolute decline in FVC ≥ 10% predicted*** | | | | |
| low RoB | 4 | 0.69 (0.59, 0.82) P<0.01 | 0.69 (0.58, 0.81) P<0.01 | 0% |
| All studies | 5 | 0.69 (0.59, 0.82) P<0.01 | 0.69 (0.58, 0.81) P<0.01 | 0% |
| ***Annual rate of decline in FVC (ml/yr)*** | | | | |
| low RoB | 2 | 73.39 (8.62, 138.15) P=0.03 | 70.95 (43.01, 98.89) P<0.01 | 81% |
| All studies | 3 | 74.88 (32.15, 117.61) P<0.01 | 72.65 (47.46, 97.84) P<0.01 | 63% |
| ***Annual rate of decline in FVC% predicted*** | | | | |
| low RoB | 1 | 1.20 (0.09, 2.31) P=0.03 | 1.20 (0.09, 2.31) P=0.03 | NA |
| All studies | 3 | 1.59 (0.72, 2.45) P<0.01 | 1.59 (0.72, 2.45) P<0.01 | 0% |
| ***Absolute change in DLCO% predicted*** | | | | |
| low RoB | 2 | 0.54 (-1.64, 2.71) P=0.63 | 0.28 (-0.95, 1.51) P=0.65 | 64% |
| All studies | 4 | 1.57 (-1.32, 4.47) P=0.29 | 0.49 (-0.73, 1.70) P=0.43 | 63% |
| ***Absolute change in 6MWD (m)*** | | | | |
| low RoB | 2 | 28.69 (10.63, 46.75) P<0.01 | 28.69 (10.63, 46.75) P<0.01 | 0% |
| All studies | 3 | 29.12 (12.58, 45.67) P<0.01 | 29.12 (12.58, 45.67) P<0.01 | 0% |
| ***Absolute change in total score of SGRQ*** | | | | |
| low RoB | 2 | -1.99 (-9.14, 5.15) P=0.58 | -2.80 (-4.31, -1.30) P<0.01 | 95% |
| All studies | 4 | -3.26 (-7.48, 0.96) P=0.13 | -3.10 (-4.45, -1.75) P<0.01 | 87% |
| ***Acute exacerbation of ILDs within 12 months*** | | | | |
| low RoB | 1 | 0.54 (0.17, 1.71) P=0.29 | 0.54 (0.17, 1.71) P=0.29 | NA |
| All studies | 5 | 0.43 (0.23, 0.82) P=0.01 | 0.38 (0.20, 0.73) P<0.01 | 0% |
| ***Adverse event: diarrhea*** | | | | |
| low RoB | 3 | 2.56 (2.23, 2.93) P<0.01 | 2.58 [2.25, 2.95] P<0.01 | 0% |
| All studies | 8 | 2.01 (1.44, 2.80) P<0.01 | 2.41 [2.12, 2.75] P<0.01 | 57% |
| ***Adverse event: nausea*** | | | | |
| low RoB | 2 | 2.65 (2.02, 3.49) P<0.01 | 2.67 (2.08, 3.43) P<0.01 | 16% |
| All studies | 6 | 2.33 (1.61, 3.36) P<0.01 | 2.54 (2.04, 3.16) P<0.01 | 45% |
| ***Adverse event: vomiting*** | | | | |
| low RoB | 2 | 2.81 (1.88, 4.20) P<0.01 | 2.81 (2.05, 3.83) P<0.01 | 36% |
| All studies | 5 | 2.77 (2.06, 3.73) P<0.01 | 2.82 (2.10, 3.79) P<0.01 | 0% |
| ***Adverse event: elevation of transaminases*** | | | | |
| low RoB | 2 | 1.90 (0.44, 8.18) P=0.39 | 2.90 (1.67, 5.01) P<0.01 | 75% |
| All studies | 4 | 1.12 (0.30, 4.14) P=0.87 | 2.40 (1.45, 3.97) P<0.01 | 64% |
| ***Adverse events leading to treatment discontinuation*** | | | | |
| low RoB | 3 | 1.99 (1.50, 2.64) P<0.01 | 2.02 (1.53, 2.68) P<0.01 | 0% |
| All studies | 7 | 2.05 (1.57, 2.68) P<0.01 | 2.10 (1.61, 2.74) P<0.01 | 0% |
| **Exploratory outcome** | | | | |
| ***Respiratory-related mortality*** | | | | |
| low RoB | 3 | 0.58 (0.10, 3.38) P=0.54 | 0.60 (0.19, 1.90) P=0.38 | 32% |
| All studies | 8 | 0.96 (0.36, 2.54) P=0.93 | 0.89 (0.39, 2.07) P=0.79 | 0% |

DLCO = diffusing capacity of the lung for carbon monoxide; FVC = forced vital capacity; ILD = interstitial lung disease; MD = mean difference; NA = not available; RR = risk ratio; SGRQ = St. George’s Respiratory Questionnaire; 6MWD = six-minute walk distance.

**Table 5. GRADE evaluation of evidence in patients with a progressive fibrosing phenotype**

| Certainty Assessment | | | | | | No. of Patients | | Effect | | Certainty | Importance |
| --- | --- | --- | --- | --- | --- | --- | --- | --- | --- | --- | --- |
| No. of Studies | RoB | Inconsistency | Indirectness | Imprecision | Other considerations | Antifibrotic drugs | Placebo | RR  (95% CI) | Absolute (95% CI) |
| Absolute change in FVC (ml) | | |  |  |  |  |  |  |  |  |  |
| 3 | Not serious | Not serious | Not serious | Not serious | None | 203 | 220 | ... | MD 100.01 higher (97.97 higher to 102.05 higher) | High | Critical |
| All-cause mortality | | |  |  |  |  |  |  |  |  |  |
| 3 | Not serious | Not serious | Not serious | Serious^a^ | None | 18/523 (3.4%) | 23/518 (4.4%) | 0.78 (0.43-  1.42) | 10 fewer per 1000 (from 25 fewer to 18 more) | Moderate | Critical |
| SAEs | | |  |  |  |  |  |  |  |  |  |
| 3 | Not serious | Not serious | Not serious | Serious^a^ | None | 151/523 (28.9%) | 165/518 (31.9%) | 0.91 (0.76-1.09) | 24 fewer per 1000 (from 66 fewer to 23 more) | Moderate | Critical |
| Absolute change in FVC% predicted | | |  |  |  |  |  |  |  |  |  |
| 3 | Not serious | Serious^b^ | Not serious | Not serious | None | 203 | 220 | ... | MD 3.38 higher (1.24 higher to 5.53 higher) | Moderate | Important |
|  | | | | | | | | | | | |

^a^TSA suggested inconclusive results.

^b^The *I^2^* across studies was 43% and TSA indicated a diversity D^2^ of 50%.

FVC = forced vital capacity; GRADE = Grading of Recommendations, Assessment, Development and Evaluations; IPF = idiopathic pulmonary fibrosis; MD = mean difference; RCT = randomized controlled trial; RoB = risk of bias; RR = risk ratio; SAEs = serious adverse events.

**Figure 1. Funnel plot for all-cause mortality**

The funnel plot was conducted when a meta-analysis including at least 10 studies.


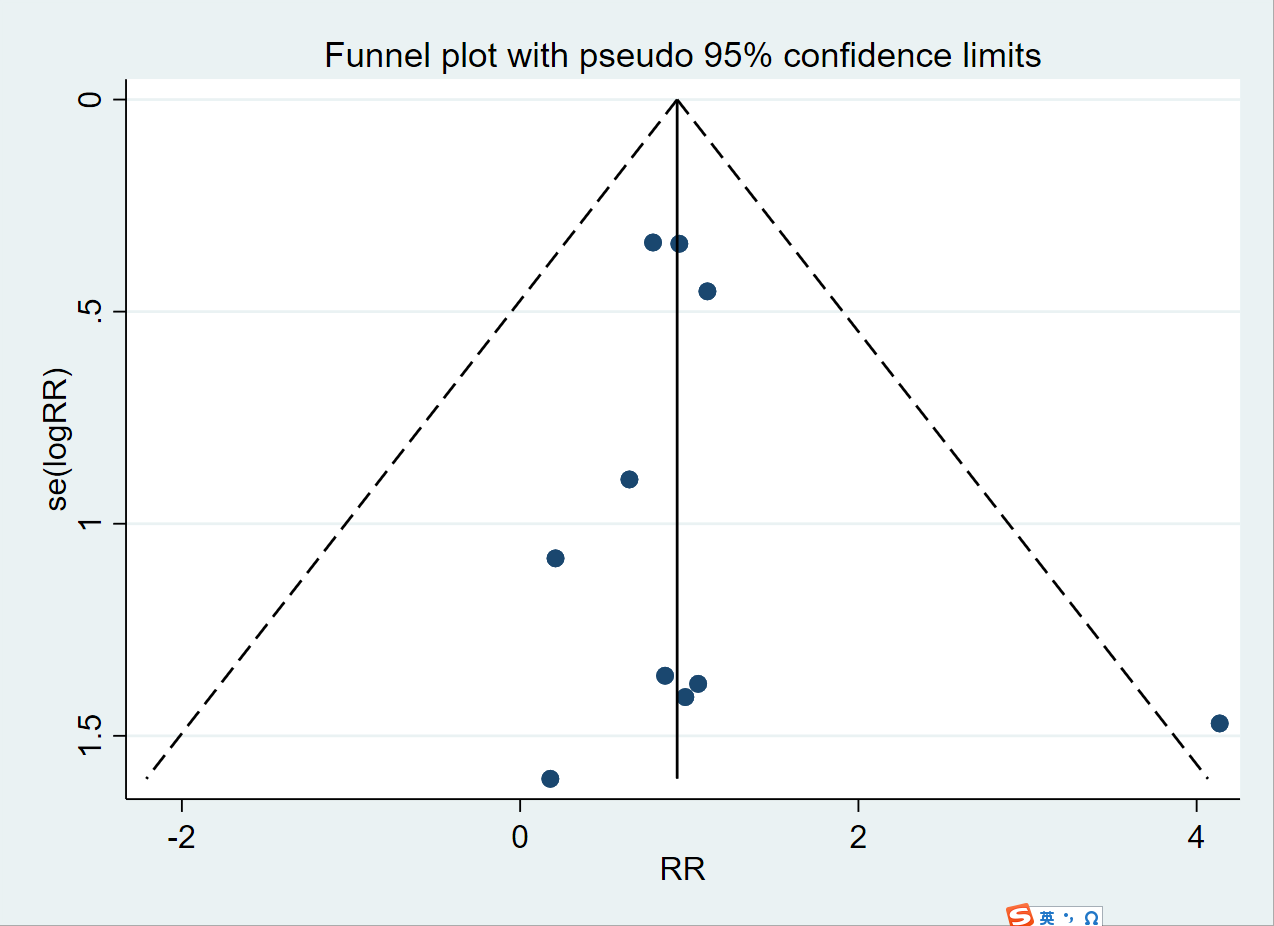


**Figure 1.** Funnel plot for all-cause mortality (10 studies)

**References**

1. Li T, Guo L, Chen Z, Gu L, Sun F, Tan X, et al. Pirfenidone in patients with rapidly progressive interstitial lung disease associated with clinically amyopathic dermatomyositis. Sci Rep. 2016;6:33226.

2. Wang J, Wang X, Qi X, Sun Z, Zhang T, Cui Y, et al. The Efficacy and Safety of Pirfenidone Combined With Immunosuppressant Therapy in Connective Tissue Disease-Associated Interstitial Lung Disease: A 24-Week Prospective Controlled Cohort Study. Front Med (Lausanne). 2022;9:871861.

3. Jakobsen JC, Wetterslev J, Winkel P, Lange T, Gluud C. Thresholds for statistical and clinical significance in systematic reviews with meta-analytic methods. BMC Med Res Methodol. 2014;14:120.

4. Distler O, Highland KB, Gahlemann M, Azuma A, Fischer A, Mayes MD, et al. Nintedanib for Systemic Sclerosis-Associated Interstitial Lung Disease. N Engl J Med. 2019;380(26):2518-28.

5. van den Hoogen F, Khanna D, Fransen J, Johnson SR, Baron M, Tyndall A, et al. 2013 classification criteria for systemic sclerosis: an American college of rheumatology/European league against rheumatism collaborative initiative. Ann Rheum Dis. 2013;72(11):1747-55.

6. Flaherty KR, Wells AU, Cottin V, Devaraj A, Walsh SLF, Inoue Y, et al. Nintedanib in Progressive Fibrosing Interstitial Lung Diseases. N Engl J Med. 2019;381(18):1718-27.

7. Acharya N, Sharma SK, Mishra D, Dhooria S, Dhir V, Jain S. Efficacy and safety of pirfenidone in systemic sclerosis-related interstitial lung disease-a randomised controlled trial. Rheumatol Int. 2020;40(5):703-10.

8. Maher TM, Corte TJ, Fischer A, Kreuter M, Lederer DJ, Molina-Molina M, et al. Pirfenidone in patients with unclassifiable progressive fibrosing interstitial lung disease: a double-blind, randomised, placebo-controlled, phase 2 trial. Lancet Respir Med. 2020;8(2):147-57.

9. Mateos-Toledo H, Mejía-Ávila M, Rodríguez-Barreto Ó, Mejía-Hurtado JG, Rojas-Serrano J, Estrada A, et al. An Open-label Study With Pirfenidone on Chronic Hypersensitivity Pneumonitis. Arch Bronconeumol (Engl Ed). 2020;56(3):163-9.

10. Behr J, Prasse A, Kreuter M, Johow J, Rabe KF, Bonella F, et al. Pirfenidone in patients with progressive fibrotic interstitial lung diseases other than idiopathic pulmonary fibrosis (RELIEF): a double-blind, randomised, placebo-controlled, phase 2b trial. Lancet Respir Med. 2021;9(5):476-86.

11. Shebl E HT. Evaluation of the efficacy of pirfenidone in progressive chronic hypersensitivity pneumonitis. The Egyptian Journal of Bronchology. 2021;15(1):1-7.

12. Fernández Pérez ER, Crooks JL, Lynch DA, Humphries SM, Koelsch TL, Swigris JJ, et al. Pirfenidone in fibrotic hypersensitivity pneumonitis: a double-blind, randomised clinical trial of efficacy and safety. Thorax. 2023;78(11):1097-104.

13. Rimner A, Moore ZR, Lobaugh S, Geyer A, Gelblum DY, Abdulnour RE, et al. Randomized Phase 2 Placebo-Controlled Trial of Nintedanib for the Treatment of Radiation Pneumonitis. Int J Radiat Oncol Biol Phys. 2023;116(5):1091-9.

14. Solomon JJ, Danoff SK, Woodhead FA, Hurwitz S, Maurer R, Glaspole I, et al. Safety, tolerability, and efficacy of pirfenidone in patients with rheumatoid arthritis-associated interstitial lung disease: a randomised, double-blind, placebo-controlled, phase 2 study. Lancet Respir Med. 2023;11(1):87-96.

15. Aletaha D, Neogi T, Silman AJ, Funovits J, Felson DT, Bingham CO, 3rd, et al. 2010 Rheumatoid arthritis classification criteria: an American College of Rheumatology/European League Against Rheumatism collaborative initiative. Arthritis Rheum. 2010;62(9):2569-81.

16. Sontheimer RD. Would a new name hasten the acceptance of amyopathic dermatomyositis (dermatomyositis siné myositis) as a distinctive subset within the idiopathic inflammatory dermatomyopathies spectrum of clinical illness? J Am Acad Dermatol. 2002;46(4):626-36.

17. Lundberg IE, Tjärnlund A, Bottai M, Werth VP, Pilkington C, de Visser M, et al. 2017 European League Against Rheumatism/American College of Rheumatology Classification Criteria for Adult and Juvenile Idiopathic Inflammatory Myopathies and Their Major Subgroups. Arthritis Rheumatol. 2017;69(12):2271-82.

18. Shiboski CH, Shiboski SC, Seror R, Criswell LA, Labetoulle M, Lietman TM, et al. 2016 American College of Rheumatology/European League Against Rheumatism classification criteria for primary Sjögren's syndrome: A consensus and data-driven methodology involving three international patient cohorts. Ann Rheum Dis. 2017;76(1):9-16.

19. Fraenkel L, Bathon JM, England BR, St Clair EW, Arayssi T, Carandang K, et al. 2021 American College of Rheumatology Guideline for the Treatment of Rheumatoid Arthritis. Arthritis Rheumatol. 2021;73(7):1108-23.

20. Sverzellati N, Lynch DA, Hansell DM, Johkoh T, King TE, Jr., Travis WD. American Thoracic Society-European Respiratory Society Classification of the Idiopathic Interstitial Pneumonias: Advances in Knowledge since 2002. Radiographics. 2015;35(7):1849-71.

21. Matteson EL, Kelly C, Distler JHW, Hoffmann-Vold AM, Seibold JR, Mittoo S, et al. Nintedanib in Patients With Autoimmune Disease-Related Progressive Fibrosing Interstitial Lung Diseases: Subgroup Analysis of the INBUILD Trial. Arthritis Rheumatol. 2022;74(6):1039-47.

22. Flaherty KR, Wells AU, Cottin V, Devaraj A, Inoue Y, Richeldi L, et al. Nintedanib in progressive interstitial lung diseases: data from the whole INBUILD trial. Eur Respir J. 2022;59(3).
